# Unusual Water Oxidation Mechanism via a Redox-Active Copper Polypyridyl Complex

**DOI:** 10.1021/acs.inorgchem.3c00477

**Published:** 2023-03-29

**Authors:** Daan den Boer, Andrey I. Konovalov, Maxime A. Siegler, Dennis G. H. Hetterscheid

**Affiliations:** † Leiden Institute of Chemistry, Leiden University, Einsteinweg 55, 2300 RA, Leiden, The Netherlands; ‡ Department of Chemistry, Johns Hopkins University, 3400 North Charles Street, Baltimore, Maryland 21218, United States

## Abstract

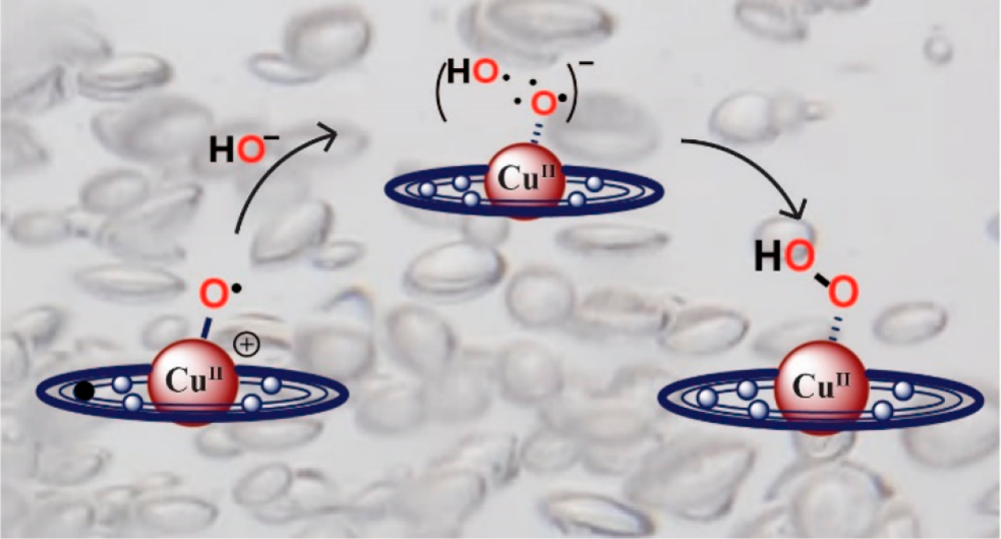

To improve Cu-based water oxidation (WO) catalysts, a proper mechanistic understanding of these systems is required. In contrast to other metals, high-oxidation-state metal–oxo species are unlikely intermediates in Cu-catalyzed WO because π donation from the oxo ligand to the Cu center is difficult due to the high number of d electrons of Cu^II^ and Cu^III^. As a consequence, an alternative WO mechanism must take place instead of the typical water nucleophilic attack and the inter- or intramolecular radical–oxo coupling pathways, which were previously proposed for Ru-based catalysts. [Cu^II^(H**L**)(OTf)_2_] [H**L** = Hbbpya = *N*,*N*-bis(2,2′-bipyrid-6-yl)amine)] was investigated as a WO catalyst bearing the redox-active H**L** ligand. The Cu catalyst was found to be active as a WO catalyst at pH 11.5, at which the deprotonated complex [Cu^II^(**L**^–^)(H_2_O)]^+^ is the predominant species in solution. The overall WO mechanism was found to be initiated by two proton-coupled electron-transfer steps. Kinetically, a first-order dependence in the catalyst, a zeroth-order dependence in the phosphate buffer, a kinetic isotope effect of 1.0, a Δ*H*^⧧^ value of 4.49 kcal·mol^–1^, a Δ*S*^⧧^ value of −42.6 cal·mol^–1^·K^–1^, and a Δ*G*^⧧^ value of 17.2 kcal·mol^–1^ were found. A computational study supported the formation of a Cu–oxyl intermediate, [Cu^II^(**L**^•^)(O^•^)(H_2_O)]^+^. From this intermediate onward, formation of the O–O bond proceeds via a single-electron transfer from an approaching hydroxide ion to the ligand. Throughout the mechanism, the Cu^II^ center is proposed to be redox-inactive.

## Abstract 2

The Cu-bbpya complex [Hbbpya = *N*,*N*-bis(2,2′-bipyrid-6-yl)amine)] is an active catalyst for the water oxidation reaction. Formation of the O−O bond occurs via an attack of a hydroxide on a Cu−oxyl species and umpolung of the transition state, leading to a quadruplet CuOOH species with an unpaired electron at Cu and two unpaired electrons at the peroxide moiety. During the catalytic reaction, the Cu site retains its II+ oxidation state, while all redox chemistry occurs on the organic and oxide ligands.

## Introduction

**Figure 1:**
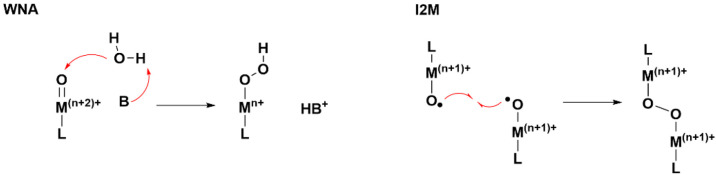
Two types of O–O bond formation mechanisms.

The global energy crisis requires the utilization of sustainable energy to replace fossil fuels and stop global warming.^1−7^ One promising sustainable energy carrier is dihydrogen, which can be produced by water splitting using renewable energy sources such as solar energy. However, the activation of water, a rather inert molecule, is a great challenge and still remains one of the most important tasks of modern chemistry. Water oxidation (WO) forming dioxygen, in which four protons and four electrons (2 H_2_O → O_2_ + 4H^+^ + 4e^–^) are produced, is the bottleneck reaction in the water-splitting process. The utilization of an efficient and cheap water oxidation catalyst (WOC) is required to enable the production of dihydrogen as an energy carrier on a large scale. Molecular Ru- and Ir-based electrocatalysts have been reported as WOCs with low overpotentials and high turnover numbers.^8−11^ However, in the past decade considerable progress has been made in the utilization of first-row transition metals Mn, Fe, Co, Ni, and Cu as cheaper and earth-abundant alternatives for the expensive Ru- and Ir-based WOCs.^12−18^ Since the first reported homogeneous Cu-based catalyst in 2012,^19^ Cu complexes have attracted increasing attention as catalysts for the oxidation of water.^20,21^ Mononuclear Cu-based WOCs are reported with bipyridine-type,^19,22−25^ alkylamine-type,^26−29^ pyridine/amine-type,^30−46^ peptide-type^47−50^ and porhyrin-type^51^ ligands. In addition, dinuclear,^44,52−55^ trinuclear,^56,57^ and tetranuclear^58−60^ Cu-based WOCs have been reported. Despite all of these publications, reports on Cu-catalyzed WO often lack detailed mechanistic information, especially compared to the mechanistically well-studied Ru-based systems. For the latter systems, it has been well-established that O–O bond formation occurs via water nucleophilic attack (WNA) or the inter- or intramolecular coupling between two metal–oxo or metal–oxyl units (I2M) (Figure 1).^61−63^ An important element herein is the formation of an electrophilic oxo group through π donation from the oxo ligand to the empty d orbitals of a high-valent Ru species.

Whereas Mn- and Fe-based WOCs are likely to follow reaction paths similar to that of Ru,^64,65^ for Cu-based WOCs, these mechanisms are quite unlikely. The formation of a high-valence Cu–oxo species is in disagreement with the oxo wall principle.^66−72^ The oxo wall is an imaginary border between the group 8 and 9 transition metals in the periodic table. The oxo wall principle describes that transition metal–oxo complexes in *C*_4*v*_ symmetry on the left side of the oxo wall can form metal–oxo species with double-bond character, M=O. On the right side of the oxo wall, for high-oxidation-state complexes (d^*n*^, where *n* ≥ 5) with the same *C*_4*v*_ symmetry, a metal–oxo double bond cannot be formed due to occupation of the π* orbitals of the metal center. Because Cu lies far beyond the oxo wall, the formation of a Cu–oxyl radical species with single-bond character in the form of M–O^•^ is expected. It is therefore doubtful that the oxyl radical is sufficiently electrophilic to allow a WNA or I2M mechanism to occur. The formation of a Cu^IV^ intermediate is rather unlikely. On the other hand, Cu^III^ complexes have been reported multiple times.^73−80^ However, the existence of d^8^ Cu^III^ complexes is questionable. In a thorough study, the Lancaster group has spectroscopically and computationally investigated 17 Cu complexes with formal oxidation states ranging from Cu^I^ to Cu^III^ without finding any diagnostic evidence for the presence of Cu^III^, suggesting that most of these species should probably be reformulated as Cu^II^ species.^81^ Therefore, the formation of Cu^*n*+^=O (*n* = III or IV) is rather unlikely, and the true active species for WO is expected to have Cu^*n*+^–O^•^ (*n* = II or III) character.^72,82,83^ Two protons and one electron need to be removed from an initial Cu^II^–OH_2_ species to produce a Cu^II^–O^•^ intermediate. The utilization of redox-active ligands allows for the accumulation of sufficient redox equivalents while avoiding the buildup of a high oxidation state on the metal center. Examples of redox-active ligands used in WO catalysis have been reported for Ru-,^84−86^ Co-,^87,88^ Ni-,^89,90^ and Cu-based^23,35,41−43,91^ catalysts. The utilization of redox-active ligands in combination with Cu sites has led to the formulation of a variety of alternative mechanistic pathways via which WO is expected to occur.^92^ In all of these pathways, single-electron transfer (SET) from an incoming hydroxide ion to the oxidized catalytic intermediate takes a central role. In the literature, this reaction step is often indicated as SET-WNA but thus far has predominantly been shown to occur upon attack of a hydroxide ion; hence, we prefer a SET-HA (hydroxide attack) terminology.^93^ In this mechanism, O–O bond formation proceeds via two consecutive SET steps. After the first SET from the hydroxide ion to the oxidized Cu complex, an intermediate is formed with a two-center three-electron (2c3e, symbolized as ∴) bond between the two O atoms with a formal oxidation state of 1.5– for each O atom.^93−96^ The formation of 2c3e bonds is unusual in WO chemistry; therefore, a brief description of this bond is given. The 2c3e bond is based on the valence bond theory by Pauling, which describes that stability arises due to resonance between the two Lewis structures by charge transfer.^96−99^ Recent studies based on the Pauling valence bond theory lead to formulation of the charge-shift bond, a new type of bonding besides the covalent and ionic bonds.^96,100−104^ The total bond energy of the 2c3e charge-shift bond is obtained from the resonance of the charge shift between the valence bond structures. Here none of the valence bond structures themselves have any bonding, and in each valence bond, the three electrons maintain Pauli repulsion. The molecular orbital (MO) scheme of a species with a 2c3e bond contains two electrons in the bonding MO and one in the antibonding MO, leading to a bond order of 0.5.^95^

**Figure 2:**
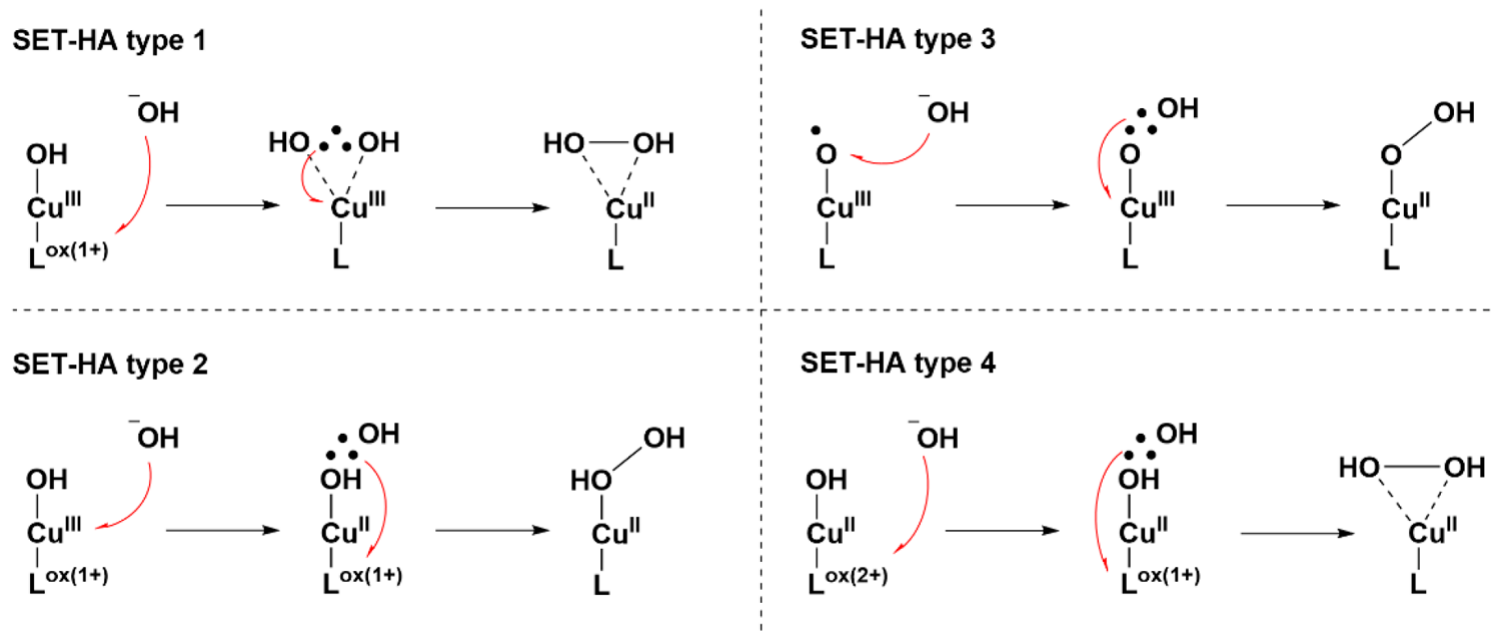
Four types of SET-HA O–O bond formation mechanisms.

Four variations on the SET-HA mechanism have been postulated in the literature, which we have classified as type 1, 2, 3, or 4 (Figure 2).^46^ A SET-HA type 1 mechanism has been proposed for WO catalyzed by [Cu^II^(*N*1,*N*1′-(1,2-phenylene)bis(*N*2-methyloxalamide))]^2–^ (Figure 2).^41,93,105,106^ A SET from a hydroxide ion to the oxidized ligand of a L^ox(+)^–Cu^III^–OH intermediate is proposed. The ligand is reduced, and a (HO∴OH)^−^ moiety is formed. In the second step, intramolecular SET from the (HO∴OH)^−^ moiety to the Cu^III^ intermediate occurs, reducing the Cu center to a II+ oxidation state. This results in the formation of a Cu^II^–(HO–OH) intermediate. The SET-HA type 1 reported by the research groups of Llobet and Maseras is the first example in the literature.^41^ Computational research by these groups on two previously reported catalysts shows that WO mediated by these species occurs via the SET-HA type 2 and 3 pathways. A SET-HA type 2 is proposed for WO catalyzed by [Cu(2,2′-bipyridine-6,6′-bis(olate))(OH_2_)_2_].^23^ In this mechanism, a SET from a hydroxide ion to the Cu^III^ center of the L^ox(+)^–Cu^III^–OH intermediate is proposed. The Cu center is reduced to a II+ oxidation state, and a (HO∴OH)^−^ bond is formed (Figure 2).^93^ A subsequent SET from the (HO∴OH)^−^ bond to the oxidized ligand, reduces the ligand and a Cu^II^–(HO–OH) intermediate is formed. A SET-HA type 3 is proposed for WO catalyzed by [Cu(2,2′-bipyridine)(OH)_2_].^19^ Because the 2,2′-bipyridine ligand is considered to be redox-inactive, L–Cu^III^–O^•^ is proposed as the active intermediate (Figure 2). In this mechanism, a SET from a hydroxide ion to the oxyl ligand is proposed to form a (O∴OH)^2–^ bond.^93^ A second SET from this 2c3e bond to the Cu^III^ ion reduces the Cu center to a II+ oxidation state and results in the formation of a Cu^II^–(O–OH)^−^ intermediate. Although the computational study suggests that no redox-active ligand is required for a SET-HA mechanism, this catalyst requires a +750 mV overpotential to form the active species.^19^ A SET-HA type 4 mechanism was proposed for WO catalyzed by a Cu-based catalyst with a π-extended tetraamidate macrocyclic ligand^42^ and [2,2′-bipyridine]-6,6′-dicarboxamide ligands substituted with phenyl or naphthyl groups.^43^ In this proposed mechanism, the ligand is oxidized twice and the Cu center remains in the II+ oxidation state. A SET from a hydroxide ion to the redox-active ligand of the L^ox(2+)^–Cu^II^–OH intermediate results in the formation of a (HO∴OH)^−^ bond (Figure 2). A subsequent SET from the (HO∴OH)^−^ bond to the ligand results in the formation of a Cu^II^–(HO–OH) intermediate. A mechanism similar to SET-HA type 4 is also proposed for WO catalyzed by [Cu^II^(1,3-bis(2′-pyridylimino)isoindoline)]^+^.^46^ However, the mechanistic study for this catalyst was performed in a water (2.0 M)/acetonitrile (MeCN) solution, which makes a thorough mechanistic comparison problematic.

For Cu-based catalysts that contain redox-active ligands, the SET-HA mechanisms appear to be a more realistic pathway than the classical WNA- and I2M-type mechanisms. The ligand *N*,*N*-bis(2,2′-bipyrid-6-yl)amine (H**L**) seems to be an ideal candidate for applications in WO chemistry because H**L** contains a conjugated π system and therefore can be easily oxidized. Moreover, in the case of the Cu-based complex [Cu^II^(H**L**)(OTf)_2_], deprotonation of the amine function occurs at a relatively mild pH of 9.5. Both properties are beneficial for a SET-HA mechanism. On top of that, H**L** has already been successfully utilized in a WOC in combination with Co and Fe (i.e., [(MeOH)Fe(H**L**)-μ-O-(H**L**)Fe(MeOH)](OTf)_4_) (MeOH = methanol).^107,108^ In this paper, [Cu^II^(H**L**)](OTf)_2_ is investigated mechanistically as a WOC in a combined experimental and theoretical study.

## Results and Discussion

### Synthesis and Characterization

**Figure 3:**
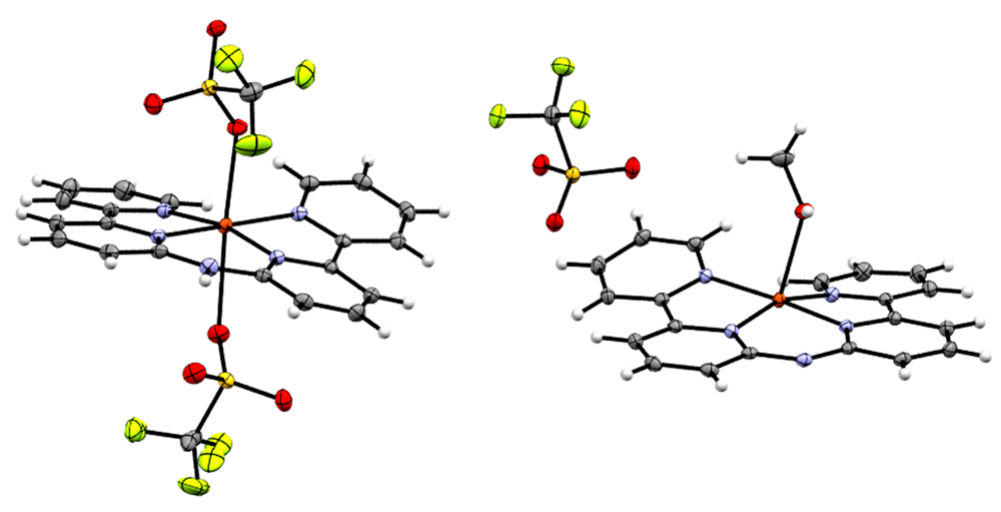
Displacement ellipsoid plot (50% probability level) of [Cu(H**L**)(OTf)_2_] (left) and [Cu(**L**)(MeOH)](OTf) (right) at 110(2) K. Disorder has been removed for clarity.

[Cu(H**L**)(OTf)_2_] was synthesized according to modified synthetic protocols (see the experimental section),^109−112^ while the synthesis of the analogous [Zn(H**L**)(OTf)_2_] was reported previously.^108^ An elemental analysis was obtained and shows that the composition of the crystalline material is in good agreement with the chemical composition and thus assignment of [Cu(H**L**)(OTf)_2_]. Crystal structures were obtained for [Cu(H**L**)(OTf)_2_], as well as for the compound [Cu(**L**)(MeOH)](OTf), which was obtained via deprotonation of [Cu(H**L**)(OTf)_2_] with NaH (Figures 3 and S1 and Tables S1 and S2). The removal of the proton on the amine moiety does not lead to any major structural changes because only minor differences in the bond lengths are obtained (Table S3). However, a significant change is observed in the bond angle around the amine moiety (C10–N3–C11), which is 131.19(14)° for [Cu(H**L**)(OTf)_2_] and 126.23(14)° for [Cu(**L**)(MeOH)](OTf). In the crystal structure of [Cu(**L**)(MeOH)](OTf), a dimer is formed via two intermolecular O–H···N hydrogen bonds [O1···N2 = 2.6726(19) Å; O1–H1O–N2 = 176(2)°] between the deprotonated amine and OH proton of the coordinated MeOH of a second [Cu(**L**)(MeOH)](OTf) unit (Figure S2). Thereby, a weak/distant π–π-stacking interaction of approximately 3.320 Å between two pyridine planes is observed within the dimer.

For solutions of [Cu(H**L**)(OTf)_2_], a square-pyramidal geometry is expected in which the two axial triflate ions are substituted for a solvent molecule (e.g., H_2_O, MeOH, or MeCN). In situations where the coordinated solvent ligand is not specifically known, the nomenclature Cu(H**L**) will be used. Cu(H**L**) was found to be stable in Milli-Q water for at least 6 days because no changes were observed in the UV–vis spectra (Figure S3). The color of the solution changed visibly from green to yellow upon the addition of a base (NaOH) to an aqueous solution of Cu(H**L**), resulting in Cu(**L**) (Figure S4). In the UV–vis spectrum, the absorbance band at 346 nm disappears and two new bands are formed at 331 and 403 nm upon deprotonation of the ligand amine (Figure S6). By a UV–vis-monitored titration with NaOH, a p*K*_a_ of 9.5 was determined for the secondary amine in Cu(H**L**) (Figure S7).

Electron paramagnetic resonance (EPR) spectra of Cu(H**L**) in MeOH were recorded at room temperature. The structurally related complex [Cu^II^(*N*,*N*′-di(pyrid-2-yl)-2,2′-bipyridine-6,6′-diamine)(H_2_O)]^2+^ has been reported under these conditions to have an isotropic *g* value of 2.11.^113^ For Cu(H**L**) in MeOH at room temperature, we found an EPR spectrum that we could simulate with *g*_iso_ = 2.11 (Figure S9; for simulation data, see Table S4). EPR spectra of Cu(H**L**) recorded in water at 130 K show an isotropic EPR signal with a *g* value of 2.06 (Figure S10). However, in a MeOH solution at 130 K, the EPR is rhombic with three *g* values of 2.200, 2.055, and 2.030 (Figure S9). No significant changes in the *g* values were found upon deprotonation with NaOMe.

### Cyclic Voltammetry in an Organic Solvent

**Figure 4:**
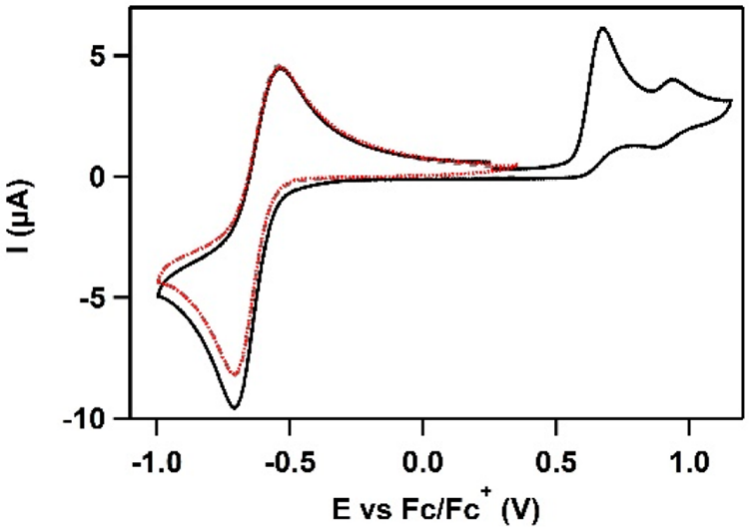
Three CVs of Cu(H**L**) in 0.1 M NBu_4_PF_6_ in MeCN at a scan rate of 100 mV·s^–1^ in varying potential windows. Cycle 1 (gray) and 3 (red) overlap exactly and were recorded between −1.0 and +0.35 V vs Fc/Fc^+^. Cycle 2 (black) was recorded between −1.0 and +1.2 V vs Fc/Fc^+^. Boron-doped diamond (BDD, 0.07 cm^2^), Au, and Ag/AgCl were used as the working electrode (WE), counter electrode (CE), and reference electrode (RE), respectively. Reference potentials were converted to Fc/Fc^+^.

A cyclic voltammogram (CV) of Cu(H**L**) was recorded in a MeCN solution under noncatalytic conditions (Figure 4). A reversible redox event assigned to the Cu^I/II^ redox couple was found at −0.64 V vs ferrocene/ferrocenium (Fc/Fc^+^). Furthermore, an irreversible oxidative wave at 0.68 V and another reversible redox event at 0.91 V vs Fc/Fc^+^ were found. The peak currents of the Cu^I/II^ redox couple and the irreversible oxidative wave were linearly dependent on the square root of the scan rate, which is in good agreement with a freely diffusive species (Figures S11 and S12). The chemistry of the intermediate formed upon irreversible oxidation was evaluated by measuring three CVs in different potential windows (Figure 4). The first CV was recorded in the potential range between −1.0 and 0.35 V vs Fc/Fc^+^, and only the Cu^I^/Cu^II^ redox couple is found (gray line, fully overlapped by the red line). In the second cycle, the potential window is increased up to 1.2 V vs Fc/Fc^+^, the irreversible oxidative wave and the second reversible redox couple are observed (black solid line). In this cycle, an enhanced current is found for the reductive wave of Cu^I/II^ at −0.64 V vs Fc/Fc^+^. In the third cycle, the potential window of −1.0 to +0.35 V vs Fc/Fc^+^ was again applied, and the current of the Cu^II^ reduction was similar to the current obtained in cycle 1 (red dotted line). The reductive current in the second scan is thus enhanced, indicating that the species that is obtained by irreversible oxidation of the Cu^II^ compound, is stable under these conditions, and is immediately reduced to the Cu^I^ species.

### Cyclic Voltammetry under Catalytic Conditions

**Figure 5:**
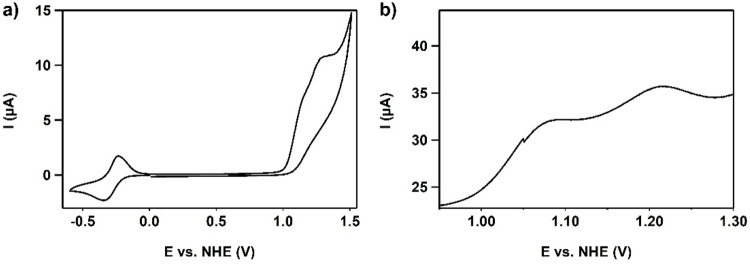
(a) CV of 0.3 mM Cu(**L**) in a 100 mM pH 11.5 phosphate buffer at a scan rate of 100 mV·s^–1^. BDD (0.07 cm^2^), Au, and reversible hydrogen electrode (RHE) were used as the WE, CE, and RE, respectively. Reference potentials were converted to NHE. (b) Differential-pulse voltammogram of the catalytic wave, visualizing two oxidative waves [recorded on a glassy carbon (GC) WE under the same conditions as the CV].

A CV of Cu(L) was recorded in an aqueous phosphate solution of pH 11.5. A quasi-reversible wave with a relatively broad reductive peak and a sharper oxidative peak (Δ*E* = 100 mV) is found at −0.29 V vs normal hydrogen electrode (NHE) and associated with the Cu^I^/Cu^II^ redox couple with *E*_pc_ at −0.34 V and *E*_pa_ at −0.24 V (Figure 5a). These potentials are in good agreement with the reversible redox event observed in an organic solvent.^114^ A linear correlation on the square root of the scan rate was found for both the oxidation of Cu^I^ and the reduction of Cu^II^, indicative of a freely diffusive process (Figure S13). Furthermore, a catalytic wave arises from 1.0 V vs NHE onward. Additional studies of the catalytic wave with differential-pulse voltammetry revealed two oxidative waves underneath the catalytic waves at 1.08 and 1.22 V vs NHE (Figure 5b). The catalytic current was also found to linearly correlate on the square root of the scan rate, again indicative for a free diffusive process (Figure S14).

Cyclic voltammetry experiments with the analogous Zn complex [Zn(H**L**)(OTf)_2_] were performed, showing a single irreversible oxidative wave at 1.0 V vs NHE (Figure S15). Because oxidation of Zn^II^ to Zn^III^ is very unlikely, this irreversible oxidative wave is assigned to oxidation of the ligand, illustrating that **L** is a redox-active ligand.^115^ Because the electrochemical oxidation is irreversible, a chemical process to a more stable intermediate via an EC (EC = electron-transfer step followed by chemical reaction) mechanism is expected.^116,117^ It must be noted that the p*K*_a_ of Zn(H**L**) is around 11.5, which is two pH units higher than that of Cu(H**L**) (Figure S8). We can therefore conclude that the irreversible oxidation waves observed in both MeCN (Figure 4) and aqueous solutions (Figure 5) should be assigned to a ligand-centered oxidation reaction.

To show that Cu(**L**) is indeed able to catalyze the oxidation of water to produce dioxygen, online electrochemistry mass spectrometry (OLEMS) was applied to detect the formation of oxygen.^118^ With OLEMS, gases formed at the electrode surface can be detected. For Cu(H**L**), the mass signal (*m*/*z* = 32) for dioxygen increased simultaneously with the increasing catalytic wave in the CV from the onset potential of 1.1 V vs NHE onward (Figure S16).

### Pourbaix Diagram

**Figure 6:**
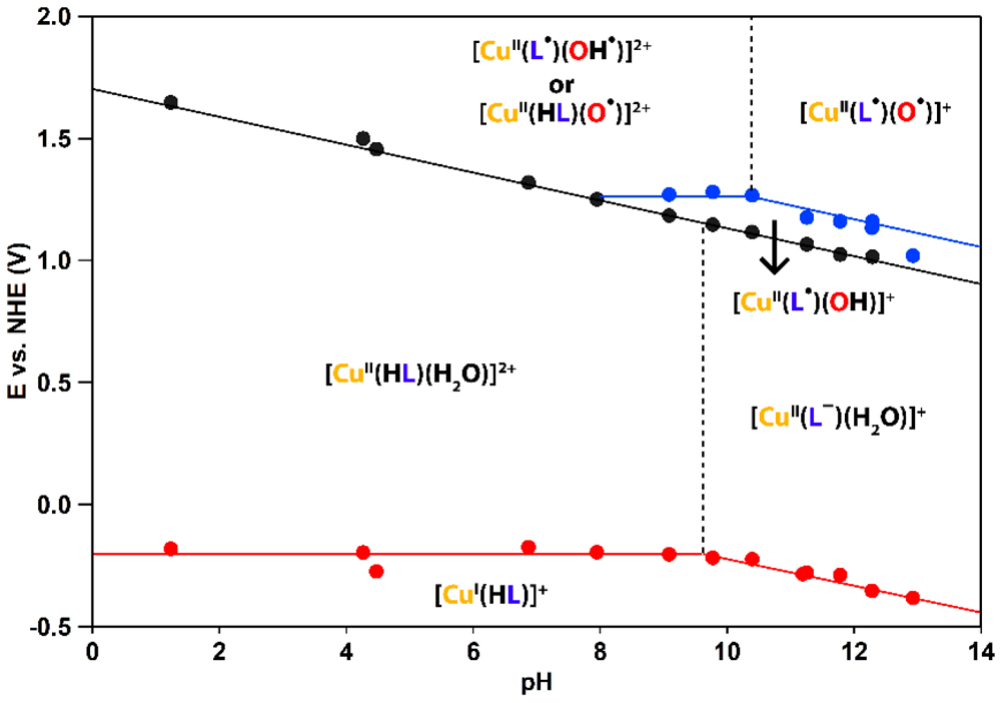
Pourbaix diagram of Cu(H**L**)/Cu(**L**) including assignments of intermediates. Data points were obtained from CVs and differential pulse voltammograms recorded in a 100 mM phosphate electrolyte solution, except for data recorded at pH 1 and 13, for which 0.1 M solutions of H_2_SO_4_ and NaOH were used, respectively.

The potentials of the redox events of Cu(H**L**) were determined as a function of the pH by cyclic and differential-pulse voltammetry (Figure 6). From the pH dependence of a given redox couple, the type of electron transfer (ET) can be determined following the Nernst equation.^116^ Slopes of 0 and −59 mV/pH units correspond to an ET and a proton-coupled electron transfer (PCET) step, respectively. [Cu^II^(**L**)(H_2_O)]^+^ can be found on the right side of the Pourbaix diagram, between pH 9.5 and 13. A pH dependence of −60 mV/pH is found for its reduction to [Cu^I^(H**L**)]^+^, indicating that this proceeds via PCET. This step is expected to occur with dissociation of a H_2_O ligand, which would lead to a stable 18-electron complex for [Cu^I^(H**L**)]^+^. Two subsequent oxidation reactions of [Cu^II^(**L**)(H_2_O)]^+^ with pH dependences of −60 and −66 mV/pH are found, which are assigned to two PCET events, respectively. These two PCET steps would lead to a formal “Cu^IV^” intermediate, which is a very unlikely species.^72,82^ Given that **L**^**–**^ is a redox-active ligand, the first oxidation step is assigned to oxidation of the ligand. Given that also the Cu^III^ oxidation state is questionable, the second oxidation reaction is assigned to the oxidation of Cu^II^–OH to Cu^II^–O^•^, leading to formation of the key oxidative species [Cu^II^(**L**^•^)(O^•^)]^+^.^81^ We anticipate that the same species is formed in an organic solvent (Figure 4), albeit in small concentrations due to the low concentration of water in MeCN, which is illustrated by the small reversible redox couple at high potential.

[Cu^II^(H**L**)(H_2_O)]^2+^ is found on the left side of the Pourbaix diagram, between pH 0 and 9.5. In this window, other pH dependences are found for the different redox events. This shift in ET types between acidic and alkaline parts of the Pourbaix diagram is correlated to the p*K*_a_ of Cu(H**L**) at pH 9.5. The reduction of [Cu^II^(H**L**)(H_2_O)]^2+^ to [Cu^I^(H**L**)]^+^ proceeds via an ET reaction, given that a dependence of 0 mV/pH is found. In contrast to the high pH window, only a single oxidative event was found with a pH dependence of −60 mV/pH. This single line in the Pourbaix diagram is expected to be the result of two redox events that at more alkaline conditions become separated and can be observed in the differential-pulse voltammogram (Figure 5b). Oxidation of [Cu^II^(H**L**)(H_2_O)]^2+^ via two PCET steps would then lead to the formation of either [Cu^II^(**L**^•^)(OH^•^)]^2+^ or [Cu^II^(H**L**)(O^•^)]^2+^, which are protonated forms of [Cu^II^(**L**^•^)(O^•^)]^+^.

Because the highest catalytic activity in cyclic voltammetry experiments can be observed between pH 10 and 13, [Cu^II^(**L**^•^)(O^•^)]^+^ is expected to be the intermediate species that is involved in O–O bond formation. Therefore, the WO mechanism of Cu(**L**) was studied in more detail in this pH window.

### Homogeneity Study

Several experiments were performed to investigate whether Cu(**L**) is a molecular catalyst.^119^ A dipping test was employed, *to rule out the formation of catalytically active heterogeneous species on the electrode surface.* After scanning 20 cycles between −0.68 and 1.31 V vs NHE in a 0.3 mM Cu(**L**) pH 11.3 phosphate solution, the electrode was rinsed to remove any remaining droplets containing Cu(**L**). Subsequently, a CV was recorded in a blank pH 11.3 phosphate buffer solution (Figure S17). In the first scan of the postcatalysis blank, a slightly higher current of 2.0 μA is observed around 1.3 V vs NHE than the current of 0.8 μA that was recorded in the initial blank before catalysis. However, this increased current of the blank is significantly lower than the catalytic current of 13.4 μA in the presence of complex Cu(**L**) in solution. The increased background current may be ascribed to roughening and oxidation of the carbon electrode surface rather than adsorption of the Cu(**L**) complex to the electrode.

To investigate the homogeneity of Cu(**L**) in more detail, electrochemical quartz crystal microbalance (EQCM) experiments were performed. EQCM is an *in situ* technique that enables the detection of mass changes on the electrode surface by changes in the oscillation frequency (Δ*f*). A negative Δ*f* corresponds to mass deposition on the electrode surface.^120^ The Cu^I/II^ redox couple showed a negative Δ*f* upon the reduction of Cu^II^(**L**) to Cu^I^(H**L**) (Figure S18). This indicates that Cu^I^(H**L**) precipitates from the solution and deposits on the electrode. Upon reoxidation to Cu^II^(**L**), a positive frequency change suggests that the deposit is redissolved. This may be linked to the expected H_2_O dissociation upon reduction of [Cu^II^(**L**)(H_2_O)]^+^ to [Cu^I^(H**L**)]^+^. Overall, there is a net frequency change of zero, pointing to all of the deposited Cu^I^(H**L**) being redissolved in the solution upon oxidation. This reversible deposition process may explain the broad reductive and sharp oxidative wave of the Cu^I/II^ redox couple in cyclic voltammetry experiments.

Subsequently, EQCM experiments were recorded of Cu(**L**) under catalytic conditions, which were compared with those of the blank phosphate buffer solution (pH 11.5). At first, cyclic voltammetry measurements were performed by scanning 50 cycles between 0.82 and 1.32 V vs NHE (Figure S19). In both cases, the same order in Δ*f* was observed, indicating a mass increase on the electrode surface. A significant change of Δ*f* is also found in the blank, which can be assigned to the interaction between gold β-oxide (formed on the electrode upon oxidation) and phosphate ions.^121^ Because Δ*f* appears to be on the same order of magnitude for the blank and Cu(**L**), deposition of Cu(**L**) seems to be limited.

**Figure 7:**
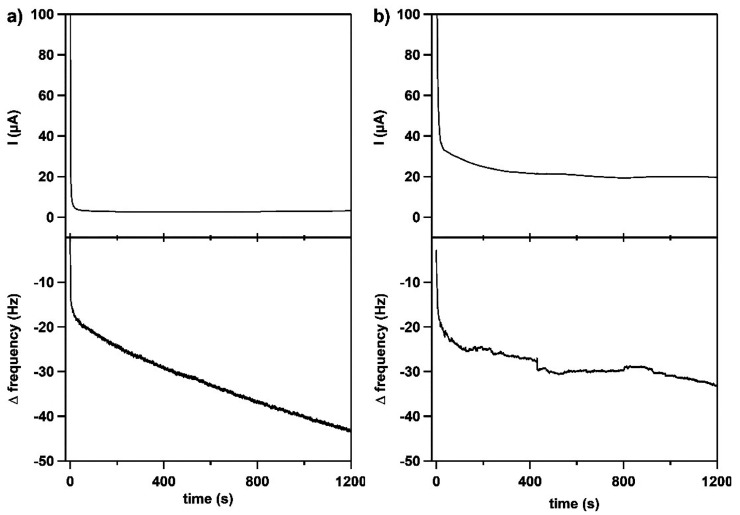
Chronoamperometry in combination with EQCM without (a) and in the presence of 0.3 mM Cu(**L**) (b). Top: Chronoamperogram at a potential of 1.22 V vs NHE in a 100 mM pH 11.5 phosphate buffer. Bottom: Δ frequency response. Au, Au, and RHE were used as the WE, CE, and RE, respectively. Reference potentials were converted to NHE.

The homogeneity of Cu(**L**) was further evaluated by performing EQCM measurements combined with chronoamperometry at 1.22 V vs NHE. Cu(**L**) was again compared to the blank phosphate buffer (Figure 7). A change in Δ*f* was observed both for a solution containing Cu(**L**) and for a blank phosphate solution devoid of any Cu(**L**). This points to an adsorption process of either hydroxide or phosphate ions on to the Au surface of the electrode. The Δ*f* of Cu(**L**) is once more on the same order of magnitude as that of the blank solution. However, in this case, the frequency profile is different in the presence of Cu(**L**), for which Δ*f* was shown to stabilize around −30 Hz, while in the blank solution, Δ*f* continuously decreases over a period of 20 min. Moreover, the Δ*f* signal obtained in the presence of Cu(**L**) is more noisy than that of the blank, which might be the result of bubble formation on the electrode surface (Figure S20). Bubble formation was not observed for the blank solution. Typical Δ*f* values that are obtained if deposition of a catalytic material is observed on the electrode typically exceed values of −100 Hz rapidly during considerably shorter chronoamperometry experiments.^108,122−124^

The postcatalysis Au EQCM electrode surface was investigated with scanning electron microscopy in combination with energy-dispersive X-ray spectroscopy (SEM-EDX) after 20 min of constant potential electrolysis (CPE) at 1.22 V vs NHE in the presence or absence of Cu(**L**). After the electrochemical experiment, the electrodes were carefully rinsed to remove the remaining catalyst and buffer solution. The electrodes were dried at 40 °C under reduced pressure for 1–3 h to remove the remaining traces of water. After chronoamperometry, no particles were found on the electrode surface in both the absence and presence Cu(**L**) (Figures S21 and S22). The EDX spectra of the postcatalysis Au electrodes show mainly the signals of Au and Si, corresponding to the electrode material and the quartz glass of the EQCM electrode, respectively (Figures S23 and S24). The absence of any Cu peaks in the EDX spectrum and no observation of particle formation on the electrode surface with SEM point toward Cu(**L**) likely being a molecular catalyst.

A bulk electrolysis experiment in the presence of Cu(**L**) was carried out in a two-compartment cell in which the WE and CE were separated by a membrane to prevent the possible cross-mixing of (by)products and reduction of oxidized byproducts at the CE. A large surface GC electrode of 0.79 cm^2^ was used to increase the conversion. Both sides of the cell were equipped with a magnetic stir bar to facilitate mass transport. Chronoamperometry at 1.20 V vs NHE was performed for 5 h (Figure S25). During the chronoamperometry, gas bubbles were formed on the electrode surface, causing signal spikes and noise in the current response. Apart from bubble-related issues, the chronoamperogram showed no depletion or increase of the overall current, indicating no major changes in the catalytic activity over time. After chronoamperometry for 5 h, only minor changes in the UV–vis and mass spectrometry spectra were observed, indicating that Cu(**L**) is still the major species present in solution (Figures S25 and S26).

### Kinetic Analysis

Kinetic experiments were performed to elucidate the mechanism of the Cu(**L**)-catalyzed WO reaction. The rate order in the catalyst was determined by measuring CVs at different concentrations of Cu(**L**) (Figure S27). A plot of the logarithm of the (baseline-corrected) current, obtained at 1.22 V vs NHE, against the logarithm of the concentration of the Cu complex results a linear regression, with a slope of 1.0 indicating a first-order dependence (Figure S27). This makes it unlikely that the WO occurs via an I2M mechanism for which a second-order dependence in the catalyst is expected.

The rate law of the reaction with respect to phosphate ions was determined in a similar fashion. CVs were recorded with varying concentrations of phosphate buffer and a constant concentration of Cu(**L**). Two sets of experiments were performed at pH values of 11.2 and 11.6. At both pH values, CVs were recorded with varying concentrations of 10–100 mM phosphate buffer. In these experiments, Na_2_SO_4_ was added in order to keep the ionic strength constant. An additional experiment was performed in 0.1 M Na_2_SO_4_ and NaOH at pH 11.6 in the absence of phosphate ions. A plot of the concentration of phosphate ions versus the measured *k*_obs_ in the CV resulted in a horizontal line, indicating zeroth-order dependence in phosphate (Figure S29). This zeroth-order dependence indicates that specific acid–base catalysis is involved in the mechanism and that the reaction rate depends on the concentration of hydroxide ions and not the concentration of phosphate ions.^125^ In the case of a WNA mechanism (Figure 1), a base (phosphate ion) is expected to activate and eventually subtract a proton from the attacking water molecule. As a consequence, the rate constant of the WO reaction via a WNA mechanism is typically dependent on the concentration of buffer.^126^ In our case, the involvement of hydroxide ions in the rate-determining step (RDS) is therefore more likely because, under the experimental conditions of pH 11.5, the concentration of hydroxide ions is an order of magnitude higher than the catalyst concentration. For the previously proposed SET-HA mechanisms (Figure 2), hydroxide ions are proposed to be involved in O–O bond formation because these catalysts operate under basic conditions.

**Figure 8:**
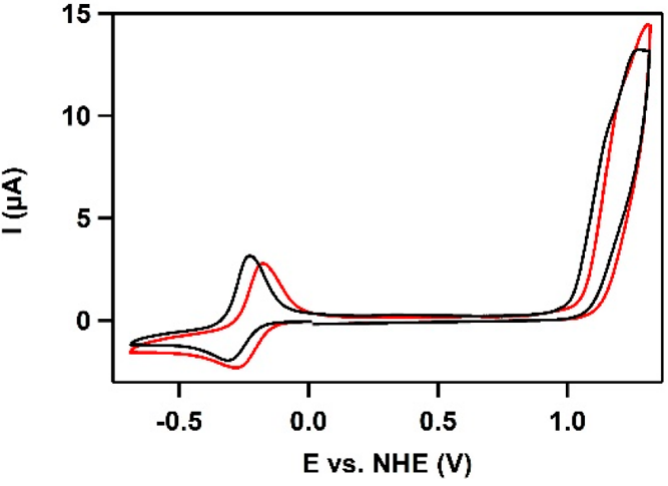
CVs of 0.3 mM Cu(**L**) in H_2_O (black) and D_2_O (red) in a 100 mM pH/pD 11.6 phosphate buffer at a scan rate of 100 mV·s^–1^. BDD (0.07 cm^2^), Au, and RHE were used as the WE, CE, and RE, respectively. Reference potentials were converted to NHE.

To further exclude WNA as the RDS, a kinetic isotope effect (KIE) was determined. The KIE is the ratio of the rate constant obtained in H_2_O and the rate constant obtained in D_2_O:
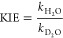


In a WNA mechanism, a proton is removed from the nucleophilic water molecule (Figure 1). The rate of this proton subtraction by a base is lower in D_2_O, due to the higher bond energy of the O–D bond than that of the O–H bond.^127^ When an O–H bond is broken during the RDS via WNA, a KIE of 2 or higher is expected.^126^ Because Cu(**L**) operates at a relatively high pH, the RDS could potentially involve a nucleophilic attack of an OH^–^ ion instead of water. If this were to be true, no O–H bond may need to be broken during the RDS, which would result in a KIE of 1.0. However, KIEs between 2 and 20 are regularly observed for Cu-based WOCs operating at pH 11.5 or higher.^34,44,45,55^ The CVs of Cu(**L**) were recorded in H_2_O and D_2_O (Figure 8). A positive potential shift in the Cu^I/II^ redox couple and onset potential is observed for Cu(**L**) in D_2_O. This potential shift is assigned to a shift in the RHE reference potential where H_2_ was bubbled through a saturated D_2_O blank solution.^128^ However, both CVs show identical current profiles, suggesting that no significant change in the WO activity upon H_2_O replacement with D_2_O takes place. Logically a KIE of 1.0 was found for Cu(**L**). The absence of a KIE is fully in line with the observed zeroth-order in phosphate ions. Based on these kinetic results, both the I2M and WNA mechanisms can be ruled out for the WO reaction mediated by Cu(**L**).

The WO reaction mediated by Cu(**L**) was further investigated by obtaining the activation enthalpy and entropy of the catalytic reaction via temperature-dependent electrochemistry in the range of 10–40 °C (Figure S30).^129^ From the Eyring equation
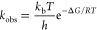
Δ*H*^⧧^ and Δ*S*^⧧^ can be determined by splitting the Δ*G* into Δ*H* and Δ*S*, resulting in a linear rewritten Eyring equation
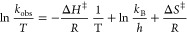
where *k*_obs_ is the rate constant in s^–1^, *R* is the universal gas constant of 8.314 J·mol^–1^·K^–1^, *T* is the absolute temperature in K, *k*_B_ is the Boltzmann constant of 1.38 × 10^–23^ J·K^–1^, *h* is the Planck constant of 6.62 × 10^–34^ J·s^–1^, Δ*H*^⧧^ is the enthalpy of activation in J·mol^–1^, and Δ*S*^‡^ is the entropy of activation in J·K^–1^·mol^–1^. The energies of Δ*H*^⧧^ and Δ*S*^⧧^ were converted from joule to calorie units.

For the WO reaction mediated by Cu(**L**), Δ*H*^⧧^ of 4.49 kcal·mol^–1^ and Δ*S*^⧧^ of −42.6 cal·mol^–1^·K^–1^ were found. From the enthalpy and entropy, the Gibbs free energy of the system was calculated to be 17.2 kcal·mol^–1^ at 298 K. To better understand the reaction mechanisms, the contribution by enthalpy and entropy is of importance.^130^ So far, Δ*H*^⧧^ and Δ*S*^⧧^ have only been obtained for WO catalyzed by Fe and Ir complexes in the presence of sacrificial oxidants.^131−134^ In these examples, Δ*H*^⧧^ and Δ*S*^⧧^ were found to vary from 10.5 to 17 kcal·mol^–1^ and from −41 to −1 cal·mol^–1^·K^–1^, respectively. Comparing the electrocatalytic WO by Cu(**L**) with chemically driven WO at Fe and Ir complexes, the enthalpy found is significantly lower and the entropy is in the high range. Mechanistically, these relatively low enthalpy and high entropy values are in agreement with a complex transition state in which two (or more) molecules need to be arranged close to each other.

### Proposed Mechanistic Cycle

The mechanistic cycle of the Cu(**L**)-catalyzed WO reaction can partly be elucidated using the obtained experimental data. Based upon these data, a WO mechanism is proposed that is initiated by two PCET steps:
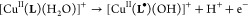
1
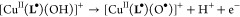
2

The first oxidation is assigned to oxidation of the ligand, based on the observation that a similar irreversible oxidation was observed for the analogous Zn complex. Based upon the kinetic analysis, first- and zeroth-order dependences were found for Cu(H**L**) and phosphate ions, respectively. Therefore, a mononuclear mechanism is expected in which phosphate does not act as a base during the RDS. Furthermore, a KIE of 1.0 is observed, indicating that the breaking or weakening of O–H bonds is not part of the RDS. Both observations exclude a typical WNA step. Because the catalytic activity is obtained only in high pH solutions, we expect OH^–^ to participate in the O–O bond formation step. The relatively low enthalpy and high entropy found for the catalytic oxidation of water by Cu(**L**) points to a RDS in which several molecules are involved.

**Figure 9:**
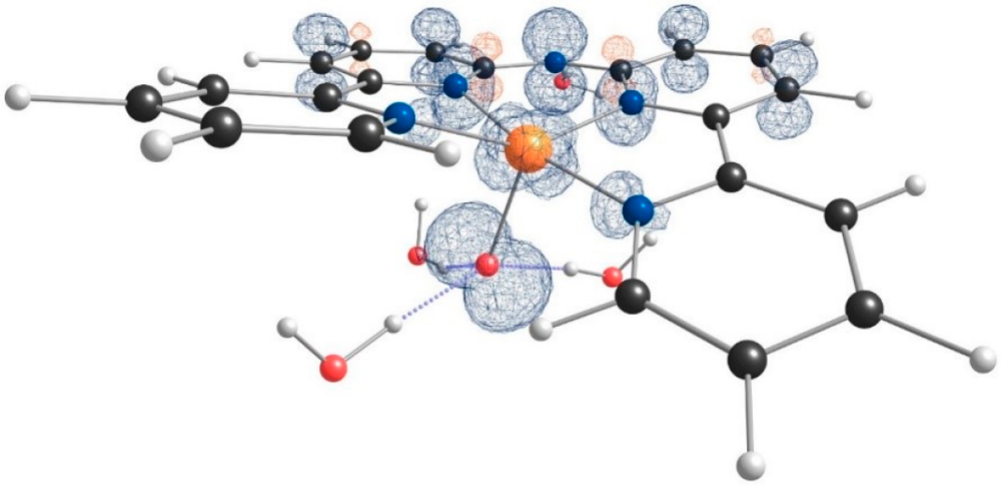
Spin-density distribution in [Cu^II^(**L**^•^)(O^•^)]^+^·4H_2_O (surface isovalue = 0.01 au) with three unpaired spins located at the Cu^II^ ion, the N bridge of the ligand, and the oxyl ligand.

To complete the missing parts of the mechanistic cycle, which were impossible to solve experimentally, computational chemistry was utilized. Spin-density calculations show that [Cu^II^(**L**^•^)(O^•^)]^+^, the proposed active intermediate for WO, has a quadruplet spin state with three unpaired electrons (Figure 9). According to density functional theory calculations, one of these three electrons is located on the d_*x*^2^–*y*^2^_ orbital of the Cu center, one is delocalized throughout the π system of the two pyridine rings and the N^–^ in the ligand, and one is present on a p orbital of the oxyl group. The redox-active ligand delocalizes the remaining radical over the ligand, retaining the Cu ion in a II+ oxidation state, even after two consecutive PCET steps. The formation of [Cu^II^(**L**^•^)(O^•^)]^+^ is in line with the oxo wall theory because a Cu^II^–O^•^ intermediate is formed rather than a Cu^III^=O species.^72,82^

**Figure 10:**
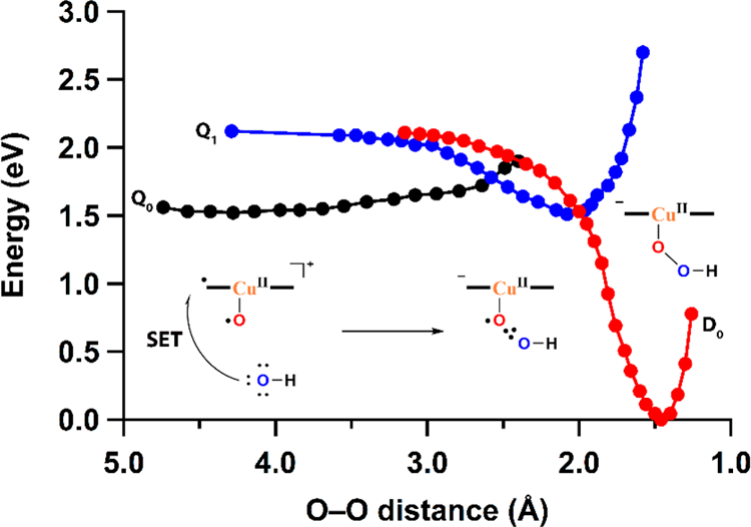
Free energy scheme of the O–O bond formation via a SET-HA mechanism starting from [Cu(L^•^)(O^•^)]^+^ and OH^–^. The black, blue, and red lines correspond to the quartet (Q_0_ and Q_1_) and doublet (D_0_) spin states of the intermediates given in the graph from left to right.

Formation of the O–O bond was computed based on the combination of [Cu^II^(**L**^•^)(O^•^)]^+^ in the quadruplet state with an OH^–^ ion. For this combination, a SET-HA mechanism is proposed, starting with a SET from the approaching hydroxide ion to the ligand (Figure 10). This returns the negative charge to the ligand and results in the formation of a 2c3e (^•^O∴OH)^−^ bond when the oxyl radical and the incoming hydroxyl radical combine (Figure S31). Next, intersystem crossing occurs wherein the species returns from the quadruplet state to a doublet state and a Cu^II^–(O–OH)^−^ intermediate is formed. The description of a charge-shift bond for the (^•^O∴OH)^−^ bond is suitable for this mechanism because a covalent bond would be repulsive.^96,100−104^

**Figure 11:**
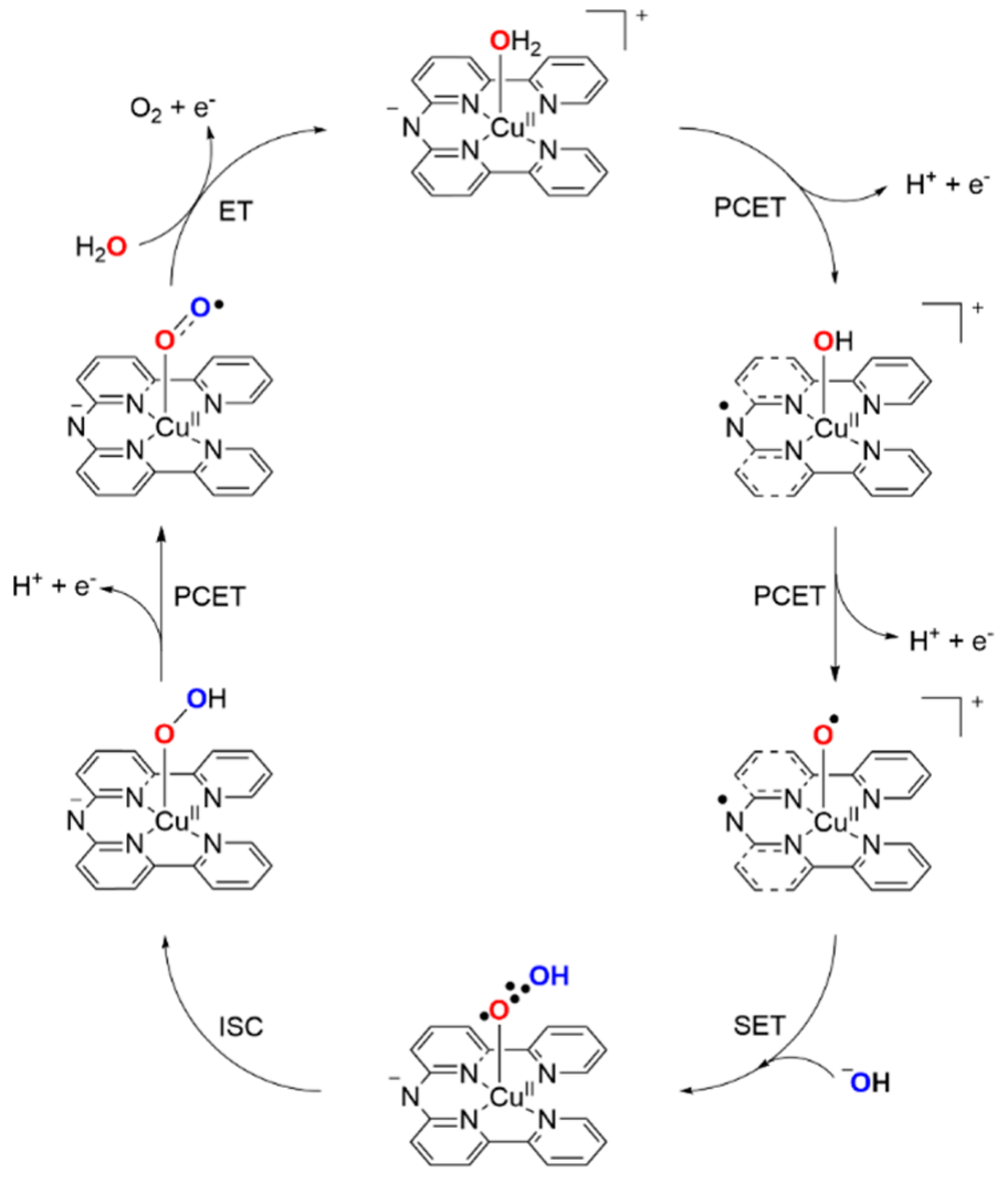
Proposed mechanistic cycle for the Cu(**L**)-catalyzed WO.

By combining experimental and computational research, a mechanistic cycle is proposed in which [Cu^II^(**L**^–^)(H_2_O)]^+^ is oxidized in two PCET steps to [Cu^II^(**L**^•^)(O^•^)]^+^ (Figures 11 and S32). Formation of the O–O bond is then expected to occur via a SET-HA mechanism. To complete the cycle, another PCET step must occur, followed by an ET step in combination with the release of O_2_. The addition of a H_2_O molecule to the free coordination site would then result in the initial species.

## Conclusion

Cu(**L**) was synthesized, characterized, and established as a molecular WOC. The ligand **L**^–^ was found to be redox-active and most likely directly participates in the reaction mechanism. Experimental observations and supporting theoretical calculations point to a mechanism that is different from WNA and I2M, which are usually proposed for Ru-based WOCs. In line with other proposed SET-HA mechanisms, we suggest a reaction path where O–O bond formation proceeds via SET from a hydroxide ion to the key L^•^–Cu^II^–O^•^ intermediate, resulting in a 2c3e bond between both O atoms, (^•^O∴OH)^−^. The utilization of redox-active ligands facilitates the ability to delocalize an electron in the π system of the ligand, which circumvents increasing the oxidation state of the Cu^II^ center to higher oxidation states.^81^ Overall, the Cu^II^ center is redox-innocent throughout the catalytic cycle. This type of SET-HA seems to be a reasonable mechanism for metal ions that are unlikely to reach high oxidation states, such as Cu.

## Supporting Information Available

Experimental, synthetic protocols, and additional figures and tables as noted in the text (PDF)
