# Supplementary material for: Unusual Water Oxidation Mechanism via a Redox-Active Copper Polypyridyl Complex
Source: Inorg Chem. 2023 Mar 29;62(14):5303–14. doi: 10.1021/acs.inorgchem.3c00477 (PMC10091478; doi:10.1021/acs.inorgchem.3c00477)
Supplement: Supplementary file 1 — ic3c00477_si_001.pdf [file ic3c00477_si_001.pdf]

Supporting information

An unusual water oxidation mechanism via a redox-active Cu polypyridyl complex

Daan den Boer,[a] Andrey I. Konovalov,[a] Maxime A. Siegler,[b] and Dennis G. H. Hetterscheid[*,a]*

[a] Leiden Institute of Chemistry, Leiden University, Einsteinweg 55, 2300 RA, Leiden, The Netherlands

[b] Department of Chemistry, Johns Hopkins University, 3400 North Charles St., Baltimore, Maryland 21218, United States

**Corresponding author:* d.g.h.hetterscheid@chem.leidenuniv.nl

Contents

I. Experimental ... 3
General information ... 3
Electrochemistry ... 3
Synthetic protocols ... 5
II. Single X-ray crystallography ... 7
III. UV-vis ... 11
IV. Electron paramagnetic resonance ... 14
V. Cyclic voltammetry ... 15
VI. On-Line Electrochemistry Mass Spectrometry ... 18
VII. Homogeneity study ... 19
VIII. Kinetic Analysis ... 24
Calculation of k_{obs} ... 24
Concentration dependence ... 25
IX. Computational study ... 27
X. NMR spectra ... 31
XI. References ... 46

I. Experimental

General information

Chemicals were purchased from Acros Organics (*tert*-butylamine), Sigma Aldrich (H_2O_2 (35% aq.), 6-Bromo-2,2'-bipyridine, $PhCF_3$, KO*t*Bu, Ts_2O, NaH (60% disp. in mineral oil), $Zn(OTf)_2$ (98%), $Pd(dba)_2$), Alfa Aesar (2,2'-bipyridine, $MgSO_4$, $Cu(OTf)_2$ (99.99%), Trifluoracetic acid), Brunswig Chemie ((*S*)-BINAP), Fluka (ferrocene, 98%), and Carl Roth GMBH (NaOH). (*S*)-BINAP and KO*t*Bu were stored under argon in a glovebox. All other compounds were used as received without any further purification.

All solvents used in the synthesis were acquired from Alfa Aesar (*tert*-butyl methyl ether), VWR (dichloromethane, Et_2O, toluene), Honeywell (MeOH, $CHCl_3$), Biosolve (MeCN, HPLC grade) and were used as received without additional purification. Dried solvents were stored in flame-dried glassware over activated 3 Å molecular sieves (Alfa Aesar). Column chromatography was performed on silica (70-230 µm mesh, 60 Å pore size) using the eluent systems described below. TLC was carried out on Merck silica gel F-254 plates. The synthesis of [Zn(H**L**)$(OTf)_2$] has been reported.[1]

NMR spectra (^{1}H, ^{13}C APT, COSY, HSQC, and HMBC) were recorded on a Bruker Avance 400 MHz Ultrashield NMR Spectrometer. Chemical shifts (δ) are reported in parts per million (ppm) using the residual solvent as internal standard. Coupling constants (*J*) are reported in Hertz (Hz). Mass spectra were obtained by LC-MS (Thermo Finnigan AQA ESI-MS). UV-Vis spectra were measured on a Varian Cary 50 Spectrophotometer in a quartz cuvette (path length 1 cm). EPR spectra were acquired on a Bruker EMXplus X-band Spectrometer. EPR spectra were simulated using the W95EPR program developed by Prof. F. Neese (MPI Muelheim). Elemental analysis was performed by Mikroanalytisches Laboratorium Kolbe in Oberhausen, Germany.

Electrochemistry

Milli-Q ultrapure water was used (resistivity >18.2 MΩ·cm) to clean glassware and to prepare all aqueous solutions. The pH of the electrolyte was determined with a Radiometer PHM220, which was calibrated using IUPAC standard buffers. Throughout the entire studies the ionic strength of all electrolytes was maintained at minimal 0.3 M.

Buffers and aqueous electrolyte solutions were prepared using high-purity (Suprapur® grade, Merck) chemicals: Na_2SO_4 (99.99%), NaH_2PO_4 (99.99%), Na_2HPO_4 (99.99%), $NaOH{\cdot}H_2O$ (99.99%), H_2SO_4 (98% aq.), HNO_3 (65% aq.), and $HClO_4$ (70% aq.).

All electrochemical experiments with the exception of the EQCM and bulk electrochemistry measurements were performed in custom made single-compartment glass cells with a three-electrode setup. All glassware was routinely cleaned from any organic residues by submerging the glassware in $KMnO_4$ solution (1 g/L $KMnO_4$ in 0.5 M H_2SO_4) followed by rinsing with water and subsequent addition of H_2O_2 solution (35% aq.) with a few drops of concentrated H_2SO_4 to remove the traces of manganese. Afterwards, the glassware was rinsed thoroughly with water. Prior to experiments, the electrochemical cells were cleaned by submerging the glassware in boiling water at least once.

Autolab PGSTAT204 or PGSTAT128N potentiostats in combination with NOVA 2.0 software was used to perform all electrochemical measurements except for the OLEMS studies (see below), for which an IVIUM Potentiostat operated by IviumSoft software was employed.

Either a glassy carbon (GC, Autolab) rod or a boron doped diamond (BDD, Windsor Scientific Ltd) electrode was used as a working electrode (WE) in the electrochemistry experiments. The respective geometric surface areas were 0.07 cm^2 (GC and BDD) and 0.79 cm^2 (BDD). For EQCM and OLEMS experiments, Au electrodes were used (see below). The GC electrode was prepared by polishing the electrode on a microcloth with 1.0, 0.3 and 0.05 micron sizes of alumina suspensions (Buehler). After polishing the GC electrode, the excess alumina was removed by sonication of the electrode in Milli-Q water for at least 10 minutes. The BDD electrode was first sonicated in Milli-Q water for 10 minutes, followed by electropolishing in 0.1 M H_2SO_4 or 0.1 M HNO_3 solution (200 cycles between –1.0 and 2.25 V vs. RHE at 1000 mV/s).

A large surface area gold wire (99.9%, MaTeck) was used as a counter electrode (CE) in all experiments. Prior to each experiment the electrode was rinsed with water and flame-annealed.

The reference electrode (RE) was either a reversible hydrogen electrode (RHE) or a Ag/AgCl (3 M KCl, Metrohm) electrode. The RHE electrode was made up of a platinum mesh (MaTeck) in H_2-saturated (Linde, H_2 5.0) blank solution with the same pH. The cell and the reference electrode were connected via a Luggin capillary. The Ag/AgCl electrode was rinsed with water before each experiment. The exact potential shift between the Ag/AgCl and RHE electrodes in a specific electrolyte was determined by measuring the corresponding open-circuit potential. The measured potentials were converted and reported against the normal hydrogen electrode (NHE).

Before all measurements, the electrolyte solution was purged of air by bubbling argon (Linde, Ar 5.0) for at least 15 minutes. During the experiment, the cell was continuously kept under a flow of argon to prevent air from entering the electrochemical cell.

For electrochemical experiments in MeCN all glassware and electrodes were prepared as mentioned above with the following alterations. The electrochemical cell was dried at 80 °C prior to an experiment to remove the traces of water. The argon flow through the electrochemical cell was saturated with MeCN by bubbling the gas through the solvent in order to prevent evaporation of the electrolyte solution. The electrolyte solution consisted of 0.1 M $N(Bu)_4PF_6$ (Sigma-Aldrich, ≥99.0%,) in MeCN (Biosolve, HPLC grade). Ag/AgCl (3 M KCl) was the reference electrode in the experiments. After the experiment ferrocene (Fluka, 98%) was added to the electrolyte solution and potentials were reported versus the ferrocene redox couple, Fc/Fc^+.

EQCM experiments were performed in an Autolab 3 mL polyether ether ketone (PEEK) EQCM cell. A Au working electrode (Autolab) with a surface area of 0.35 cm^2 was used, consisting of a 200 nm gold layer deposited on a quartz crystal. A more detailed description of the EQCM setup is reported elsewhere.[2]

SEM images of Au (EQCM) electrodes were obtained under high vacuum with a Thermo Scientific Apreo scanning electrons microscope with an acceleration voltage of 15 kV and 0.1 nA beam current. A Schottky type field emission gun was used as electron source and secondary electrons were used to obtain SEM images. EDX spectra were recorded with a Thermo Scientific UltraDry energy dispersive X-ray detector. Investigation of multiple positions of the electrode surface with SEM show no formation of particles.

For OLEMS measurements a custom-made gold electrode (99.99%, 0.79 cm^2 disk, MaTeck) was used. The electrode was cleaned by applying 10 V between the electrode and a graphite rod in 10% aq. H_2SO_4 for 30

seconds. The electrode was then dipped in a 6 M aq. HCl solution for 20 seconds, flame-annealed, and electrochemically polished in a 0.1 M aq. $HClO_4$ solution (200 cycles with a scan range between 0.0 and 1.75 V *vs.* RHE at 1000 mV/s). The gasses formed at the electrode were collected via a hydrophobic tip (KEL-F with a porous Teflon plug) in a close proximity to the surface of the electrode and analyzed by a Balzers Quadrupole (QMS 200) mass spectrometer. The inlet tip was cleaned by submerging the tip in 0.2 $K_2Cr_2O_7$ in 2 M H_2SO_4 for 15 minutes, followed by thoroughly rinsing of the tip with water. The QMS intensity of was baseline corrected for the decreasing signal corresponding to the pump pressure.[3] In addition, an artifact observed in the linear sweep at ca. 1.16 V was due to current limitation in the operating program. A more elaborate description of the methodology can be found in the literature.[4]
Bulk electrolysis was carried out in a two-compartment cell equipped with a magnetic stirring bar in which the working and counter electrodes were separated by a Selemion™ AHO membrane (AGC Engineering Co., Ltd). For this experiment, a GC working electrode with a larger surface area (0.79 cm^2) in a hanging meniscus configuration was used. A collection of multiple Au wires was used as counter electrode to create a larger surface area then the GC working electrode. Ag/AgCl (3 M KCl) was employed as a reference electrode. A constant potential of 1.20 V vs. NHE was applied for 5 hours.
In kinetic experiments the WE was polished between each measurement. The temperature in a custom-made electrochemical reactor was controlled by an Omega RDXL45D digital thermometer.

Synthetic protocols

2,2'-bipyridine *N*-oxide

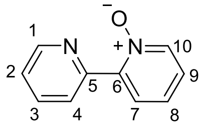

The following procedure was based on a method described in the literature.[5] 2,2'-bipyridine (3.05 g, 19.5 mmol, 1 equiv.) was dissolved in 15 mL trifluoracetic acid and cooled to 0°C. H_2O_2 (35 wt. %, 3 mL, 28.8 mmol, 1.5 equiv.) was added dropwise to the solution. The mixture was then allowed to warm up to room temperature and stirred for 4.5 hours. Afterwards, the mixture was cooled to 0°C and neutralized with 6 M NaOH. The aqueous phase was extracted four times with $CHCl_3$ (pH of the aqueous phase ≈9). The combined organic phase was dried with $MgSO_4$, filtered and concentrated *in vacuo* to yield the product as a white solid (3.08 g, 17.9 mmol, 92%). The product was stored under nitrogen atmosphere. ^{1}H NMR (400 MHz, $CDCl_3$) δ: 8.88 (dt, *J* = 8.0, 1.1 Hz, 1H, H-1), 8.71 (ddd, *J* = 4.8, 1.9, 0.9 Hz, 1H, H-4), 8.30 (dd, *J* = 6.6, 1.2 Hz, 1H, H-10), 8.16 (dd, *J* = 8.0, 2.2 Hz, 1H, H-7), 7.82 (td, *J* = 7.8, 1.8 Hz, 1H, H-2), 7.38 – 7.31 (m, 2H, H-3, H-8), 7.29 – 7.23 (m, 1H, H-9). ^{13}C NMR (101 MHz, $CDCl_3$) δ: 149.76 (C4), 149.52 (C_q), 147.47 (C_q), 140.81 (C10), 136.38 (C2), 128.00 (C7), 125.81 (C3 or C8) 125.62 (C1), 125.36 (C9), 124.40 (C3 or C8).

6-amino-2,2'-bipyridine

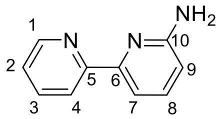

The following procedure was based on a method described in the literature.[6-7] 2,2'-bipyridine *N*-oxide (2.0 g, 11.6 mmol, 1 equiv.) was placed under nitrogen atmosphere and dissolved in 50 mL anhydrous α,α,α-trifluortoluene. The solution was cooled in an ice bath, and *tert*-butylamine (6.5 ml, 61 mmol, 5.2 equiv.) was added to the solution.

Subsequently, Ts_2O (8.87 g, 27.2 mmol, 2.3 equiv.) was added portion wise over a period of 4 hours in portion sizes of 2.5 to 3.5 g and afterwards the mixture was stirred for 1.5 hours while keeping the temperature below 0°C. Then 40 mL of trifluoracetic acid was added, and the mixture was heated at 70 °C overnight. After evaporation of the solvent (**CAUTION**: trifluoracetic acid is a corrosive acid, the solution should be concentrated in a fumehood), water and *tert*-butyl methyl ether were added to the residue. The layers were separated, and the aqueous phase was extracted twice with *tert*-butyl methyl ether, which was concentrated under reduced pressure yielding in the *p*-toluensulfonamide byproduct. The aqueous layer was basified with $NaHCO_3$ to pH 9-10 and extracted with dichloromethane (DCM). The combined organic layers were washed with saturated brine, dried with $MgSO_4$, filtered and concentrated *in vacuo*. The crude product was purified by column chromatography (DCM⟶ 3% MeOH in DCM as an eluent) resulting a light brown solid (1.10 g, 6.42 mmol, 55%). During the column chromatography *t*Bu-protected amine was isolated as well (0.31 g, 1.37 mmol). ^{1}H NMR (400 MHz, $CDCl_3$) δ: 8.66 (ddd, *J* = 4.8, 1.9, 0.9 Hz, 1H, H-1), 8.26 (dt, *J* = 8.0, 1.1 Hz, 1H, H-4), 7.77 (td, *J* = 7.8, 1.8 Hz, 1H, H-3), 7.71 (dd, *J* = 7.5, 0.8 Hz, 1H, H-7), 7.57 (t, *J* = 7.8 Hz, 1H, H-8), 7.30 – 7.22 (m, 1H, H-2), 6.54 (dd, *J* = 8.1, 0.8 Hz, 1H, H-9), 4.51 (s, 2H, NH_2). ^{13}C NMR (101 MHz, $CDCl_3$) δ: 158.13 (C10), 156.58 (C6), 154.78 (C5), 149.29 (C1), 138.75 (C8), 136.88 (C3), 123.47 (C2), 121.12 (C4), 111.79 (C7), 109.03 (C9).

***N,N*-bis(2,2'-bipyrid-6-yl)amine (HL)**

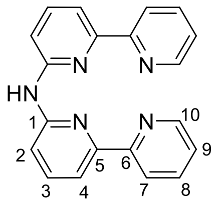

The following procedure was based on a method described in the literature.[8] 6-bromo-2,2'-bipyridine (0.50 g, 2.13 mmol, 1 equiv.) and $Pd(dba)_2$ (0.031 g, 0.054 mmol, 0.025 equiv.) were placed in a flame dried flask under nitrogen atmosphere. (S)-(-)-2,2'-bis(diphenylphosphino)-1,1'-binaphthyl (0.087 g, 0.140 mmol, 0.06 equiv.), KO*t*Bu (1.07 g, 9.5 mmol, 4.5 equiv.) and 40 mL of dry and degassed toluene were added, and the mixture was stirred for 10 minutes. Subsequently, 6-amino-2,2'-bipyridine (0.43 g, 2.49 mmol, 1.2 equiv.) was added, and the resultant mixture was heated for 4 days at 80 °C, after which it was cooled down to room temperature and quenched with 20 mL of water. The mixture was stirred vigorously for an hour and was then extracted three times with DCM. The combined organic layers were dried with $MgSO_4$, filtered and concentrated *in vacuo* resulting in a brown oil. Upon addition of cold MeOH, a brown solid precipitated from the brown oil. The solid was isolated by filtration, washed with cold MeOH and dried under vacuum at 40 °C to yield the desired product (0.39 g, 1.19 mmol, 56%). ^{1}H NMR (400 MHz, $CDCl_3$) δ: 8.70 (ddd, *J* = 4.8, 1.8, 0.9 Hz, 2H, H-10), 8.36 (dt, *J* = 8.0, 1.1 Hz, 2H, H-7), 7.98 (dd, *J* = 7.6, 0.8 Hz, 2H, H-4), 7.89 – 7.77 (m, 4H, H-3, H-8), 7.68 (dd, *J* = 8.2, 0.9 Hz, 2H, H-2), 7.51 (s, 1H, NH), 7.32 (ddd, *J* = 7.5, 4.8, 1.2 Hz, 2H, H-9). ^{13}C NMR (101 MHz, $CDCl_3$) δ: 156.25, 154.44, 153.38 (C_q), 149.27 (C10), 138.75 (C3), 136.91 (C8), 123.61 (C9), 121.03 (C7), 113.94 (C4), 111.79 (C2).

[Cu(HL)(OTf)$_2$]

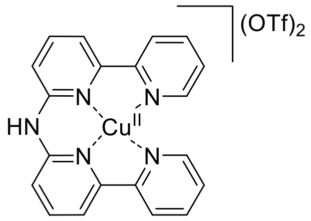

H**L** (0.25 g, 0.76 mmol, 1.05 equiv.) was dissolved in 20 mL of MeOH, to which a solution of Cu(OTf)$_2$ (0.26 g, 0,72 mmol, 1 equiv.) in 16 ml of MeOH was added. The mixture was stirred at room temperature for 2 hours and subsequently concentrated *in vacuo*. The compound was re-dissolved in MeOH and crystalized by vapor diffusion with Et$_2$O at 4°C. After 3 days, dark green crystals precipitated, which were collected by filtration, washed with Et$_2$O and dried at 40 °C under vacuum yielding the target compound (0.31 g, 0.44 mmol, 59%). Single crystals suitable for X-ray diffraction analysis were regrown by vapor diffusion in MeOH/Et$_2$O system at -20°C. MS (ESI) m/z: calc. for [Cu(H**L**)]$^{2+}$: 194.0, found 194.0; calc. for [Cu(H**L**)(MeOH)]$^{2+}$: 210.0, found 210.0; calc. for [Cu(**L**)]$^{+}$: 387.1, found 387.2; calc. for [Cu(H**L**)(OTf)]$^{+}$: 537.0, found 537.2. Elemental analysis: calculated for $C_{22}H_{15}CuF_6N_5O_6S_2$ (687.05 g/mol): C, 38.54; H, 2.23; N, 10.16%; found: C, 38.46; H, 2.20; N, 10.19%. UV-Vis (Milli-Q water): ε_{346} = 2.2·10^4 L·mol^{-1}·cm^{-1}, ε_{287} =2.7·10^4 L·mol^{-1}·cm^{-1}.

II. Single X-ray crystallography

Crystals suitable for X-ray structure determination were obtained by vapor diffusion of Et$_2$O into a methanol solution of Cu(H**L**) at –20°C. To obtain a complex with the deprotonated amine moiety, the compound was treated with an excess of NaH (60% disp. in mineral oil) in dry MeCN. Afterwards, the mixture was quenched with water, concentrated *in vacuo* and washed thoroughly with hexane. Single crystals suitable for X-ray structure determination were obtained by vapor diffusion as described above.

All reflection intensities were measured at 110(2) K using a SuperNova diffractometer (equipped with Atlas detector) with Mo *K*α radiation (λ = 0.71073 Å) under the program CrysAlisPro[i]. The same program was used to refine the cell dimensions and for data reduction. The structures were solved with the program SHELXS-2018/3 and were refined on F^2 with SHELXL-2018/3.[9-10] Numerical absorption correction based on gaussian integration over a multifaceted crystal model was applied using CrysAlisPro. The temperature of the data collection was controlled using the system Cryojet (manufactured by Oxford Instruments). The H atoms were placed at calculated positions (unless otherwise specified) using the instructions AFIX 43 or AFIX 137 with isotropic displacement parameters having values 1.2 or 1.5 U_{eq} of the attached C atoms. The H atoms attached to N or O were found from difference Fourier maps, and their coordinates were refined pseudo-freely using the DFIX instructions to keep the X–H (where X = N or O) bond distances within some acceptable ranges. The -CF$_3$ group from the less strongly coordinated TfO$^-$ anion in the structure of [Cu(H**L**)(OTf)$_2$] is disordered over two orientations, and the occupancy factor of the major component of the disorder refines to 0.52(4).[ii]

---

[i] Computer program: CrysAlisPro 1.171.39.29c, Rigaku OD, 2017

[ii] CCDC 2026076 and 2026077 contain the supplementary crystallographic data for [Cu(**L**)(MeOH)](OTf) and [Cu(H**L**)](OTf)$_2$, respectively. These data can be obtained free of charge from The Cambridge Crystallographic Data Center via https://www.ccdc.cam.ac.uk/structures/.

Table S1. Crystallographic data for [Cu(H**L**)(OTf)$_2$].

Chemical formula	$C_{22}H_{15}CuF_6N_5O_6S_2$
M (g/mol)	687.05
Crystal system, space group	Triclinic, *P*-1
Temperature (K)	110
a, *b*, *c* (Å)	9.4131 (3), 9.9425 (3), 14.4736 (4)
α, β, γ (°)	105.765 (2), 104.205 (2), 98.387 (2)
V (Å^3)	1230.54 (7)
Z	2
Radiation type	Mo *K*α
μ (mm^{-1})	1.16
Crystal size (mm)	0.39 × 0.20 × 0.15
Data collection	
Diffractometer	SuperNova, Dual, Cu at zero, Atlas
Absorption correction	*CrysAlis PRO* 1.171.39.29c[iii] Numerical absorption correction based on gaussian integration over a multifaceted crystal model Empirical absorption correction using spherical harmonics, as implemented in SCALE3 ABSPACK scaling algorithm.
T_{min}, T_{max}	0.470, 1.000
No. of measured, independent and observed [$I > 2\sigma(I)$] reflections	18749, 5635, 5297
R_{int}	0.019
$(\sin \theta/\lambda)_{max}$ (Å^{-1})	0.650
Refinement	
$R[F^2 > 2\sigma(F^2)]$, $wR(F^2)$, S	0.025, 0.065, 1.04
No. of reflections	5635
No. of parameters	419
No. of restraints	116
H-atom treatment	H atoms treated by a mixture of independent and constrained refinement
$\Delta\rho_{max}$, $\Delta\rho_{min}$ (eÅ^{-3})	0.52, -0.39

[iii] Computer program: CrysAlisPro 1.171.39.29c, Rigaku OD, 2017

Table S2. Crystallographic data for [Cu(**L**)(MeOH)](OTf).

Chemical formula	$C_{21}H_{18}CuN_5O{\cdot}CF_3O_3S$
M (g/mol)	569.01
Crystal system, space group	Triclinic, *P*-1
Temperature (K)	110
a, *b*, *c* (Å)	7.1590 (2), 12.7001 (4), 13.9768 (4)
α, β, γ (°)	111.877 (3), 103.726 (2), 96.127 (3)
V (Å^3)	1118.42 (6)
Z	2
Radiation type	Mo *K*α
μ (mm^{-1})	1.14
Crystal size (mm)	0.18 × 0.10 × 0.09
Data collection	
Diffractometer	SuperNova, Dual, Cu at zero, Atlas
Absorption correction	*CrysAlis PRO* 1.171.39.29c[iv] Numerical absorption correction based on gaussian integration over a multifaceted crystal model Empirical absorption correction using spherical harmonics, as implemented in SCALE3 ABSPACK scaling algorithm.
T_{min}, T_{max}	0.703, 1.000
No. of measured, independent and observed [$I > 2\sigma(I)$] reflections	17379, 5147, 4754
R_{int}	0.021
$(\sin \theta/\lambda)_{max}$ (Å^{-1})	0.650
Refinement	
$R[F^2 > 2\sigma(F^2)]$, $wR(F^2)$, S	0.027, 0.067, 1.07
No. of reflections	5147
No. of parameters	329
No. of restraints	1
H-atom treatment	H atoms treated by a mixture of independent and constrained refinement
$\Delta\rho_{max}$, $\Delta\rho_{min}$ (e Å^{-3})	0.43, -0.35

[iv] Computer program: CrysAlisPro 1.171.39.29c, Rigaku OD, 2017

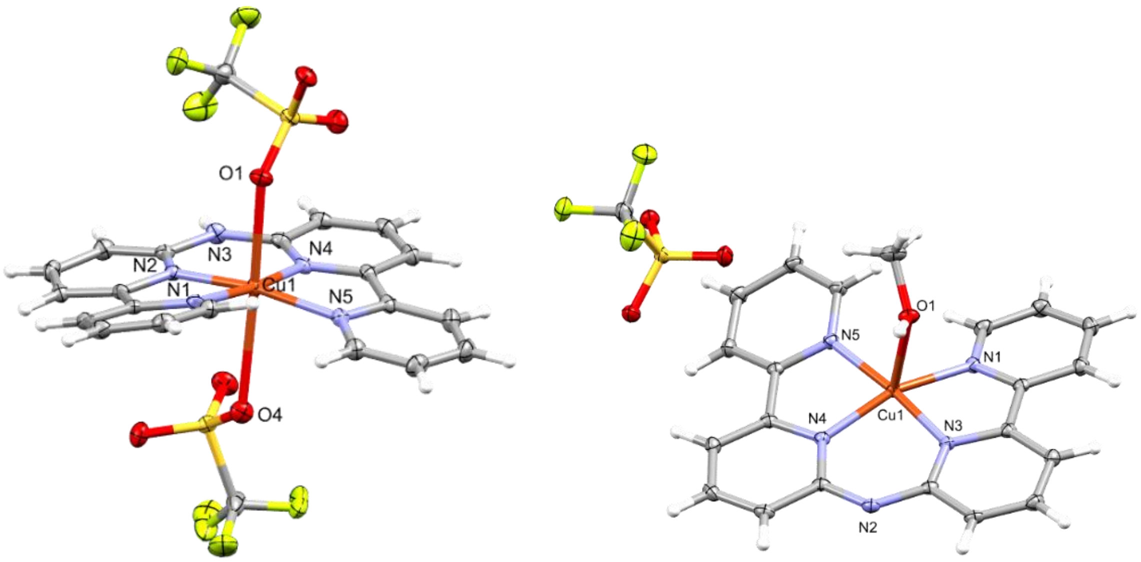

Figure S1. Crystal structures of [Cu(H**L**)(OTf)$_2$] and [Cu(**L**)(MeOH)](OTf) with thermal ellipsoids set to 50% including atom labels.

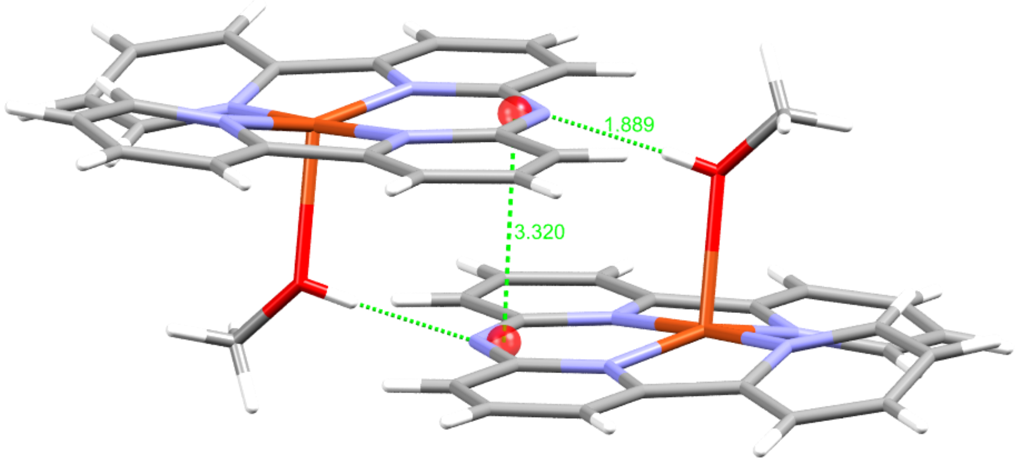

Figure S2. Crystal packing of [Cu(**L**)(MeOH)](OTf) depicting intramolecular H-bonding and a sort of π-π stacking interactions in the dimer with distances in Å. Triflate ions are omitted for clarity.

Table S3. Selected parameters for [Cu(H**L**)]$(OTf)_2$ and [Cu(**L**)(MeOH)](OTf).

	[Cu(H**L**)$(OTf)_2$]	[Cu(**L**)(MeOH)](OTf)
Cu-N1 (Å)	2.0171(13)	2.0339(14)
Cu-N2 (Å)	1.9668(13)	1.9507(14)
Cu-N4 (Å)	1.9697(13)	1.9559(14)
Cu-N5 (Å)	2.0139(14)	2.0032(14)
Cu-O1 (Å)	2.4388(12)	2.2783(12)
C10-N3 (Å)	1.376(2)	1.352(2)
C11-N3 (Å)	1.373(2)	1.354(2)
N1-Cu-N2 (°)	82.05(5)	82.47(6)
N2-Cu-N4 (°)	91.89(5)	91.42(6)
N4-Cu-N5 (°)	82.19(6)	82.65(6)
N1-Cu-N5 (°)	104.41(5)	103.11(6)
C10-N3-C11 (°)	131.19(14)	126.23(14)
N1-N2-N4-N5 (°)	11.43(7)	13.62(7)

III. UV-vis

A UV-vis spectrum was recorded directly after the preparation of a 0.3 mM aqueous solution of [Cu(H**L**)$(OTf)_2$]. The spectrum was baseline corrected for the background absorbance of water, and three absorbance bands were identified at 221, 287, and 346 nm (Figure S3). The solution was kept in a sealed cuvette for a period of 6 days, after which essentially the same spectra was obtained, suggesting that the Cu complex was stable in the aqueous solution during the corresponding period of time.

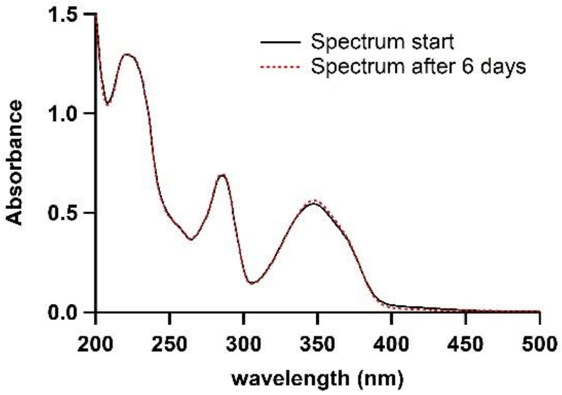

Figure S3. UV-vis spectra of 0.3 mM Cu(H**L**) in milli-Q water over a period of 6 days. Both lines overlap.

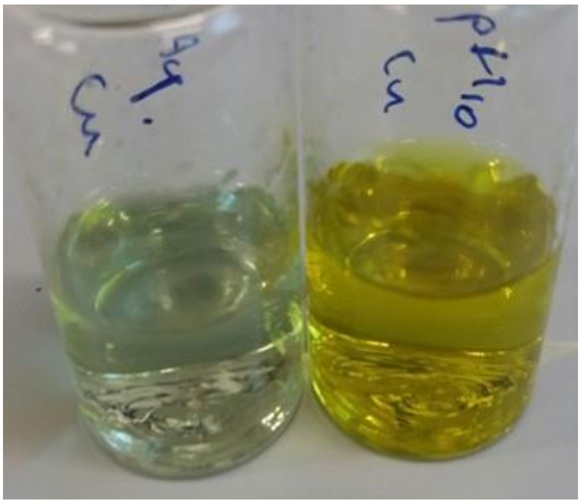

Figure S4. Solutions of Cu(H**L**) in pH neutral water (left) and Cu(**L**) in a water upon addition of NaOH (right).

The molar extinction coefficient of the Cu(H**L**) was determined. A 0.56 mM stock solution was prepared by dissolving 3.9 mg of [Cu(H**L**)(OTf)$_2$] in 10 mL of water. By dilution of the stock solution, a series of concentrations ranging from 55 µM to 5.5 µM was prepared. Subsequently, all UV-vis spectra were recorded within the range 200-500 nm (Figure S5a). The absorbance at 287 and 346 nm were plotted versus the concentration of the Cu complex resulting in linear correlation (Figure S5b). From their slopes, the following molar extinction coefficients were extracted: $2.7 \cdot 10^4$ and $2.2 \cdot 10^4$ $L \cdot mol^{-1} \cdot cm^{-1}$ for 287 and 346 nm, respectively.

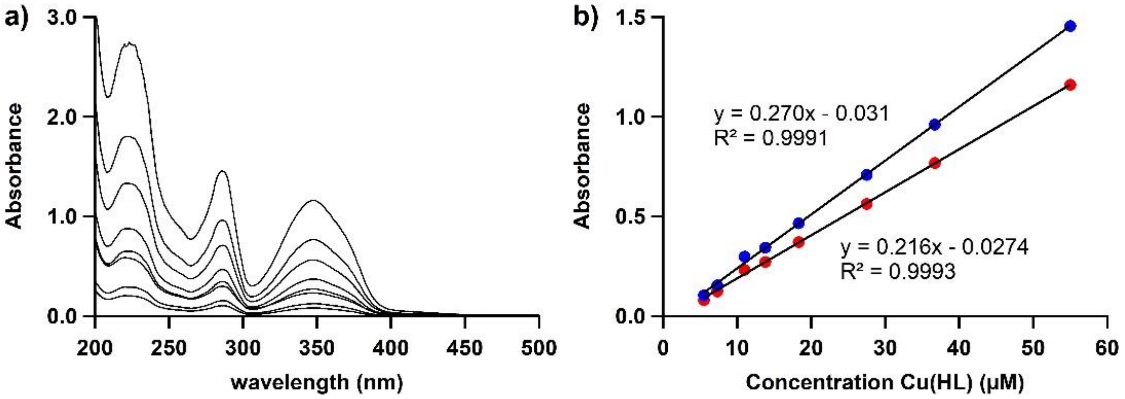

Figure S5. a) UV-vis spectra at different concentrations of Cu(H**L**) in Milli-Q water. Spectra correspond to concentrations of 55, 37, 28, 18, 14, 11, 7.3 and 5.5 µM from top to bottom. b) Linear correlation of the absorbance at 287 (black) and 346 (red) nm on the Cu(H**L**) concentration.

Subsequently, the pK_a of the Cu complex was determined by UV-Vis monitored titration with NaOH. To a 50 mL solution of 30 µM Cu(H**L**), portions of 10 to 100 µL 0.01 M NaOH were added. The combination of a respectively large volume of the Cu(H**L**) solution and small volume portions of NaOH prevented detectable dilution of the bulk solution, remaining the concentration constant and thereby the absorbance response would not diminish due to dilution. The pH was measured, and an aliquot was taken to record a UV-vis spectrum (Figure S7a), after which it was put back to the solution to maintain the total volume constant. The absorbance band appearing at 403 nm was plotted against the measured pH (Figure S7b). The equivalent point corresponds to the pK_a value of 9.5.

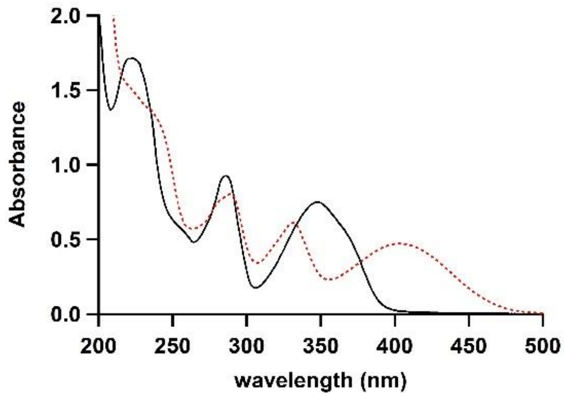

Figure S6. UV-vis spectra of a 0.4 mM Cu(H**L**) in pH 7 solution (black) and Cu(**L**) in pH 11.5 solution (red) in Milli-Q water.

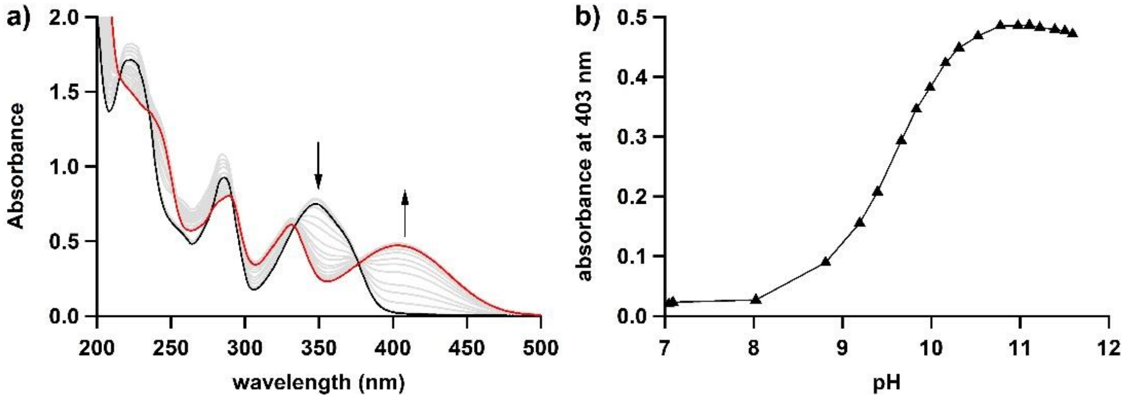

Figure S7. a) Evolution of the UV-vis spectra of Cu(H**L**) to Cu(**L**) upon titration with NaOH. b) Absorbance of Cu(**L**) at 403 nm as a function of pH.

By applying the same method and using the absorbance band with a maximum at 405 nm, the pK_a of Zn(H**L**) was determined to be 11.5 (Figure S8). Note that increase of pH above 13 induced precipitation, thereby making further titration impossible.

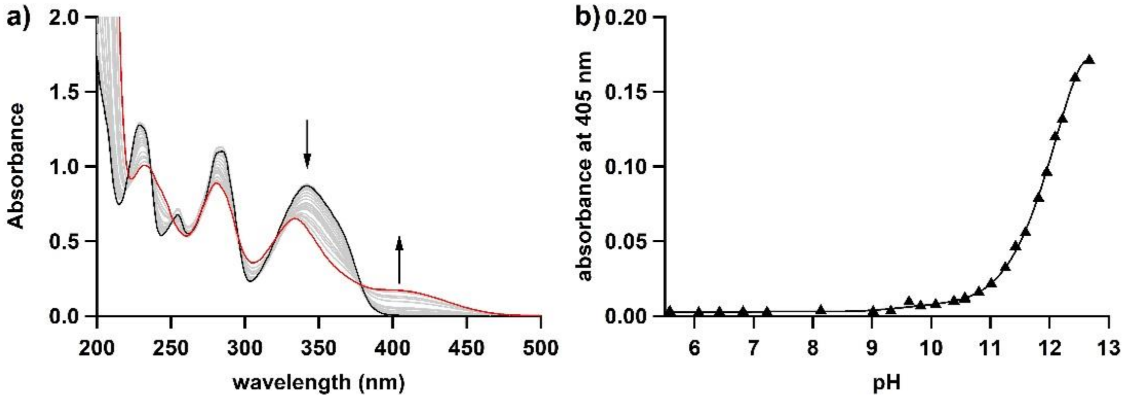

Figure S8. a) UV-vis spectra of Zn(H**L**) at different pH. b) Absorbance of Zn(**L**) at 405 nm as a function of pH.

IV. Electron paramagnetic resonance

EPR spectra were recorded in quartz glass EPR tubes. EPR spectra at 130 K were recorded under a continuous flow of nitrogen gas keeping the temperature of the sample constant. The nitrogen gas flow was cooled by passing the gas through liquid nitrogen (77 K). Simulation of the EPR parameters was carried out while considering a isotope distribution of 65% ^{63}Cu and 35% ^{65}Cu, and with gN65/gN63=1.07.

Table S4. Parameters determined by simulation of the EPR spectra. A and A^N values are given in Gauss (G).

sample	g_{iso}	g_x	g_y	g_z	A_{iso}	A_x	A_y	A_z	a_{iso}^N	a_x^N	a_y^N	a_z^N
[Cu(H**L**)(OTf)$_2$] in MeOH at 293 K	2.11				210				36			
[Cu(H**L**)(OTf)$_2$] in MeOH at 130 K[a]		2.03	2.06	2.20		13	500	20		25	20	40
[Cu(H**L**)(OTf)$_2$] in H_2O at 130 K	2.06				50				20			

[a] in presence of NaOMe

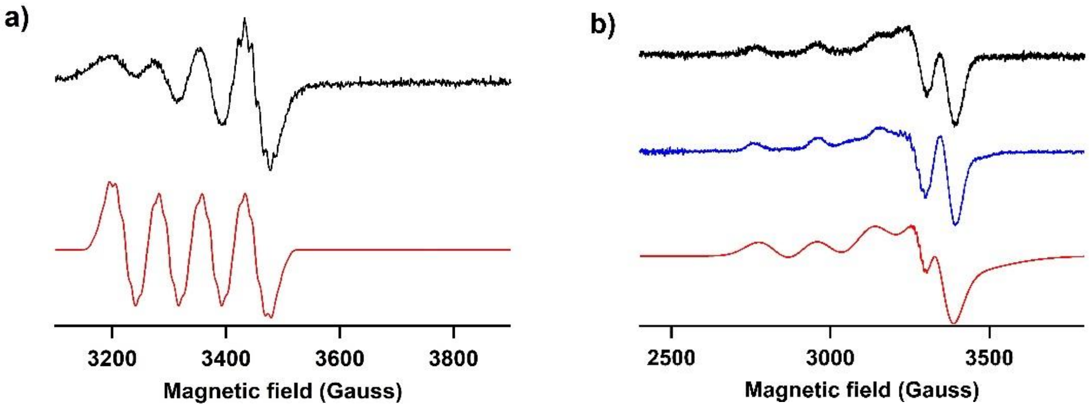

Figure S9. a) Experimental (top, black) and simulated (bottom, red) EPR spectra of a 4 mM solution of [Cu(H**L**)(OTf)$_2$] in methanol at room temperature. b) Experimental EPR spectra of a 4 mM solution of [Cu(H**L**)(OTf)$_2$] in methanol at 130 K (black) and upon addition of NaOMe to this sample (blue), and the simulated (red) EPR spectrum.

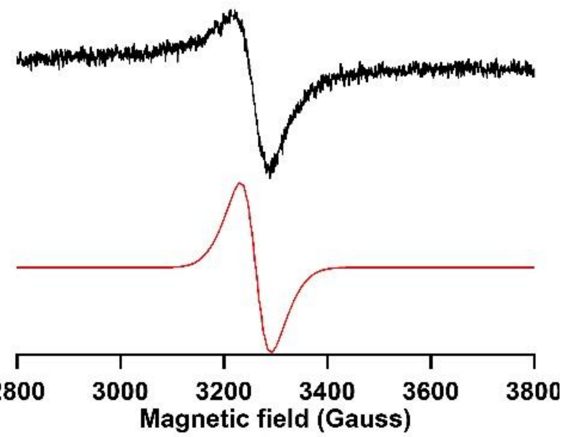

Figure S10. Experimental (top, black) and simulated (bottom, red) EPR spectra of 0.3 mM Cu(H**L**) in Milli-Q water in a frozen solution (T=130 K).

V. Cyclic voltammetry

The Cu^{I}/Cu^{II} redox couple was studied at different scan rates in a random order in order to easily distinguish between scan rate and time dependent processes. The square root of the scan rate ($v^{1/2}$) was plotted against the peak currents of the oxidative and reductive waves in the CV resulting a linear correlation, which is in line with a freely diffusive process.[11]

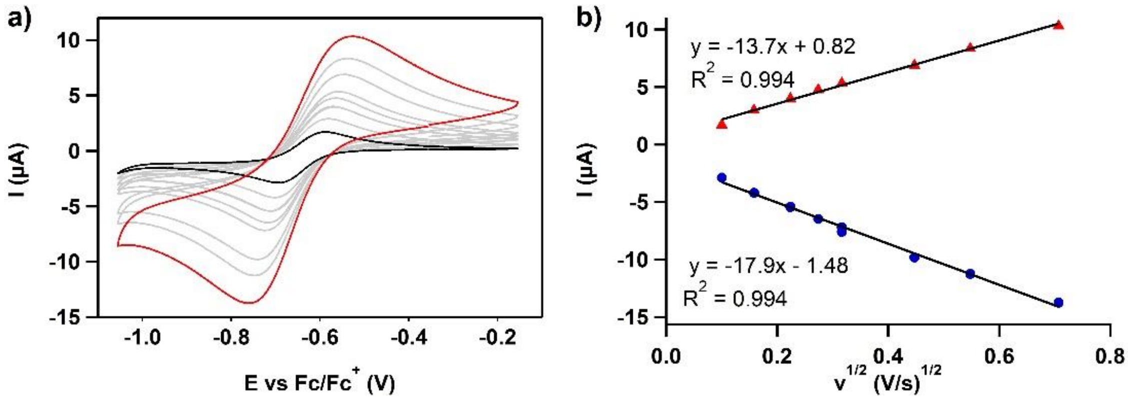

Figure S11. a) CVs of 1.0 mM Cu(H**L**) in 0.1 M NBu_4PF_6 in MeCN at varying scan rate. BDD (0.07 cm^2), Au and Ag/AgCl were used as WE, CE, RE, respectively. Reference potentials were converted to Fc/Fc^+. b) Linear correlation of the oxidative and reductive peak currents of the $Cu^{I/II}$ redox couple of Cu(H**L**) on the square root of the scan rate.

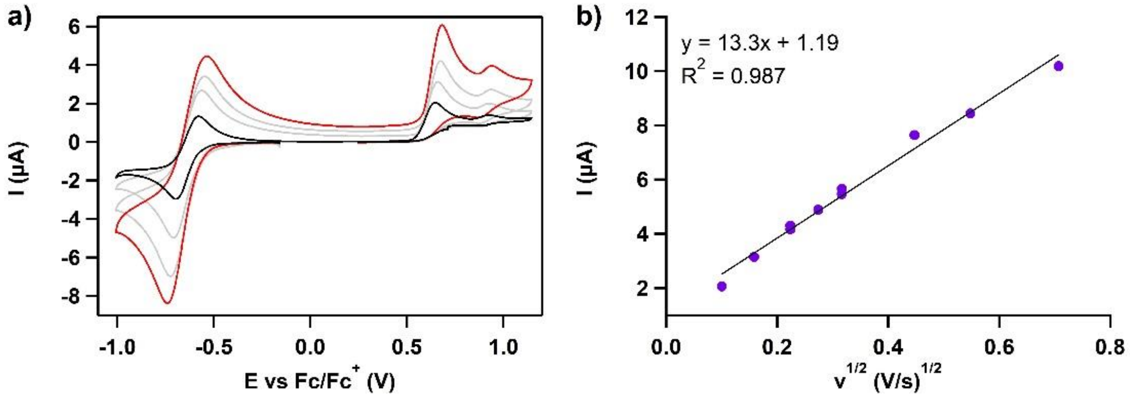

Figure S12. a) CVs of 1.0 mM Cu(H**L**) in 0.1 M NBu_4PF_6 in MeCN at varying scan rate. BDD (0.07 cm^2), Au and Ag/AgCl were used as WE, CE, RE, respectively. Reference potentials were converted to Fc/Fc^+. Not all scan rates are depicted for clarity. b) Linear correlation of the oxidative peak current of the irreversible oxidative wave of Cu(H**L**) at 0.68 V vs. Fc/Fc^+ on the square root of the scan rate.

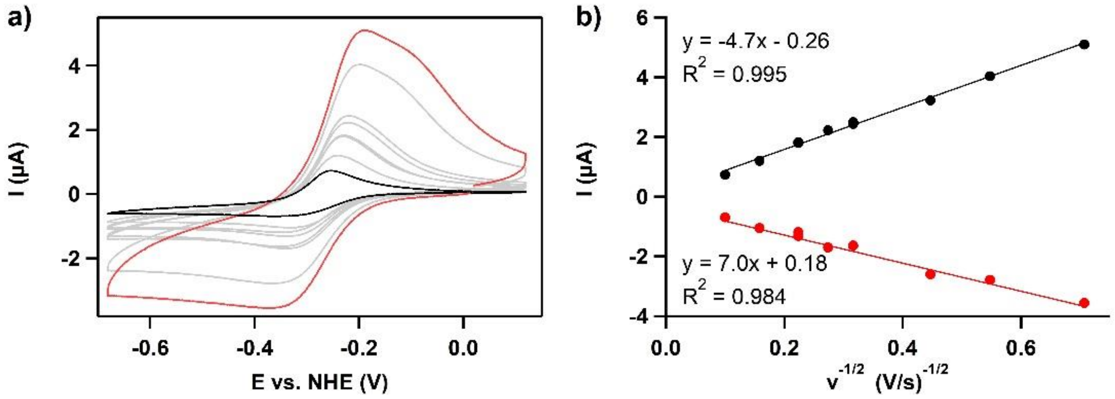

Figure S13. CVs of 0.3 mM Cu(**L**) in 100 mM pH 11.5 phosphate buffer at varying scan rate. BDD (0.07 cm^2), Au and RHE were used as WE, CE, RE, respectively. Reference potentials were converted to NHE. b) Linear correlation of the oxidative and reductive peak currents of the $Cu^{I/II}$ redox couple of Cu(**L**) on the square root of the scan rate.

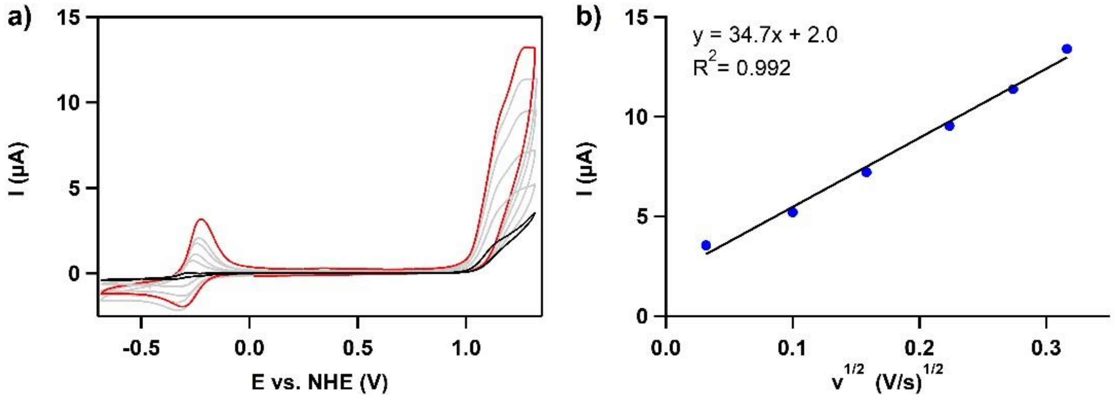

Figure S14. a) CVs of 0.3 mM Cu(**L**) in 100 mM pH 11.3 phosphate buffer at varying scan rate. BDD (0.07 cm^2), Au and RHE were used as WE, CE, RE, respectively. Reference potentials were converted to NHE. b) Linear correlation of the catalytic wave peak currents of Cu(**L**) on the square root of the scan rate.

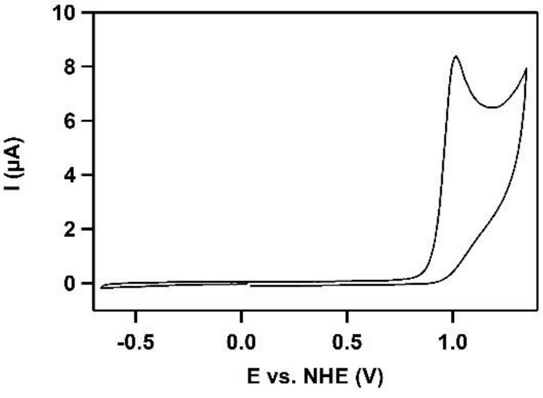

Figure S15. CV of a 0.3 mM solution of [Zn(H**L**)]$(OTf)_2$ in 100 mM pH 11.3 phosphate buffer at a scan rate of 100 mV/s. BDD (0.07 cm^2), Au and RHE were used as WE, CE, RE, respectively. Reference potentials were converted to NHE.

VI. On-Line Electrochemistry Mass Spectrometry

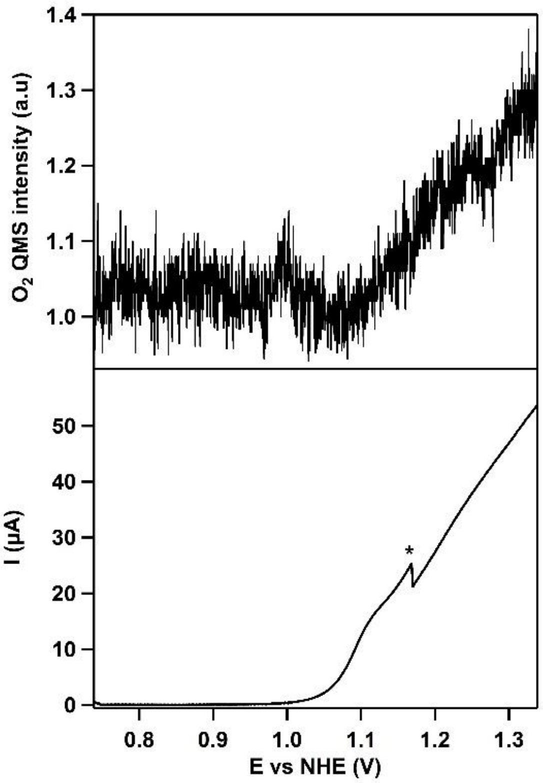

Figure S16. Top: detection of dioxygen by mass spectrometry (m/z = 32). Bottom: linear sweep of 0.4 mM Cu(**L**) in 100 mM pH 11.2 phosphate buffer at a scan rate of 1 mV/s. * = catalytic current artifact due to installation of a low current range in the software. Au, Au and RHE were used as WE, CE, RE, respectively. Reference potentials were converted to NHE.

VII. Homogeneity study

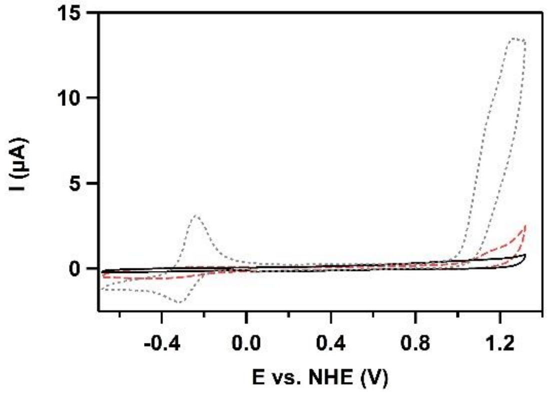

Figure S17. CVs in a blank 100 mM pH 11.3 phosphate solution at a scan rate of 100 mV/s before catalysis (solid black) and after 20 cycles in a solution containing 0.3 mM Cu(**L**) in 100 mM pH 11.3 phosphate buffer at a scan rate of 100 mV/s (red dotted). A CV in the presence of 0.3 mM Cu(**L**) in 100 mM pH 11.3 phosphate buffer is presented for comparison (grey dotted). BDD (0.07 cm^2), Au and RHE were used as WE, CE, RE, respectively. Reference potentials were converted to NHE.

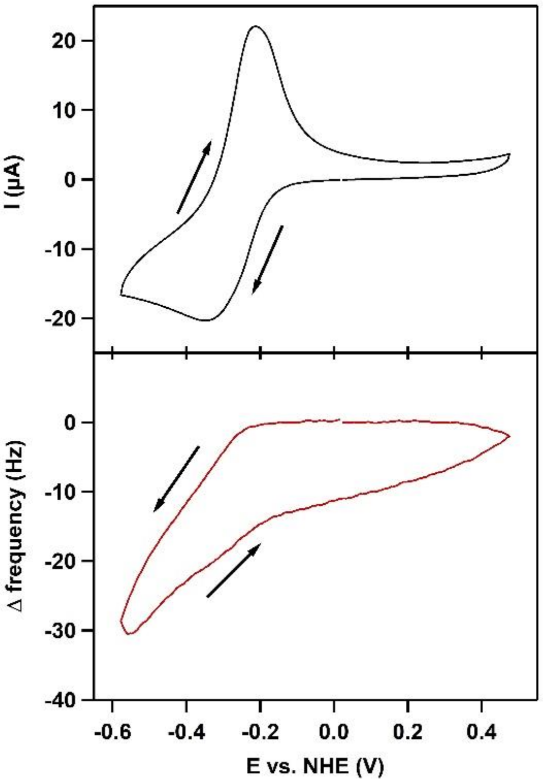

Figure S18. CV in combination with EQCM. Top: CV of 0.3 mM Cu(**L**) in a 100 mM pH 11.5 phosphate buffer at a scan rate of 100 mV/s. bottom: Δ frequency response. Au, Au and RHE were used as WE, CE and RE, respectively. Reference potentials were converted to NHE.

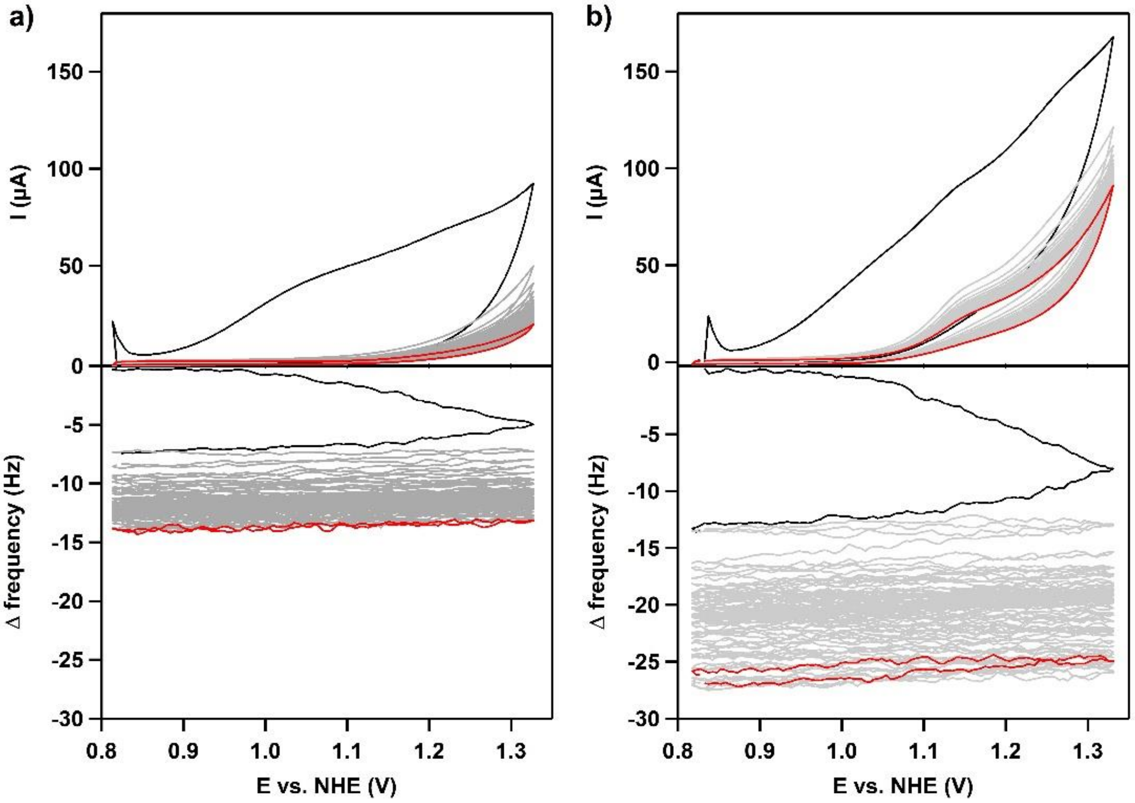

Figure S19. CVs in combination with EQCM without (a) and in the presence of 0.3 mM Cu(**L**) (b). In both graphs cycle 1 is depicted in black and cycle 50 in red. Top: CVs in a 100 mM pH 11.5 phosphate buffer at a scan rate of 100 mV/s. bottom: Δ frequency response. Au, Au and RHE were used as WE, CE and RE, respectively. Potentials were converted to NHE.

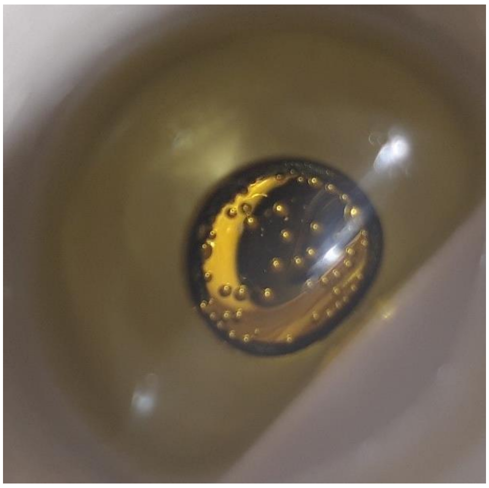

Figure S20. Formation of gas bubbles on the Au electrode surface during chronoamperometry at 1.22 V vs. NHE in the presence of Cu(**L**).

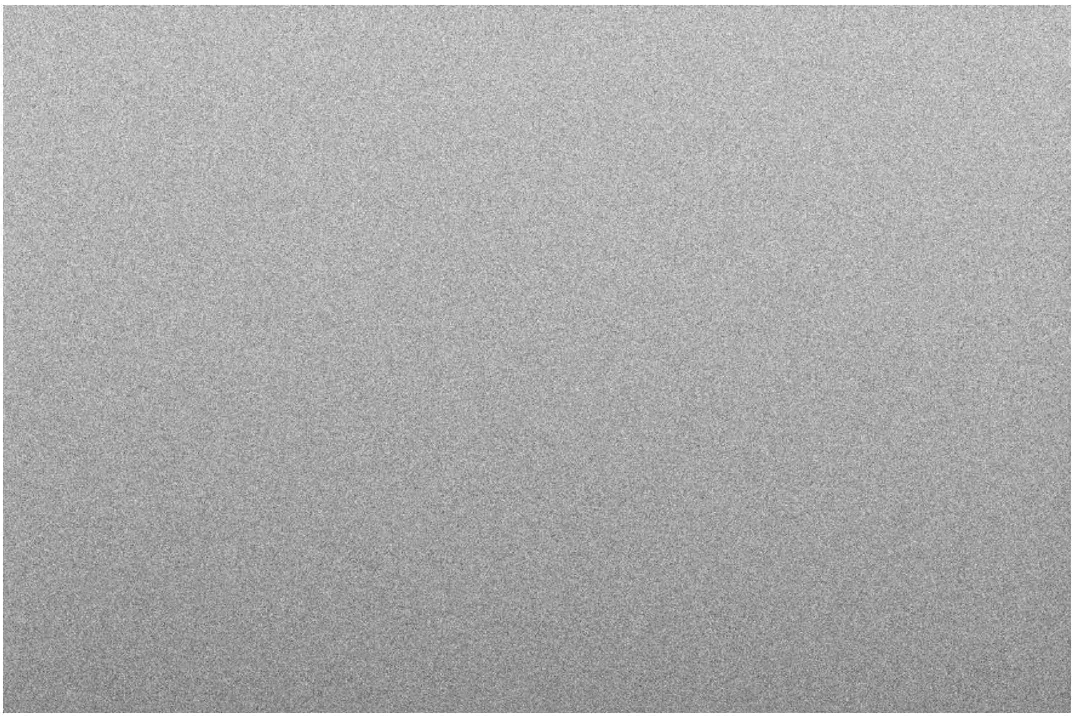

Figure S21. SEM image of Au electrode after 20 minutes of CPE at 1.22 V vs. NHE in phosphate buffer. A magnification of 2500x, a high voltage of 15.00 kV and pressure of $7.01 \cdot 10^{-6}$ mbar was applied.

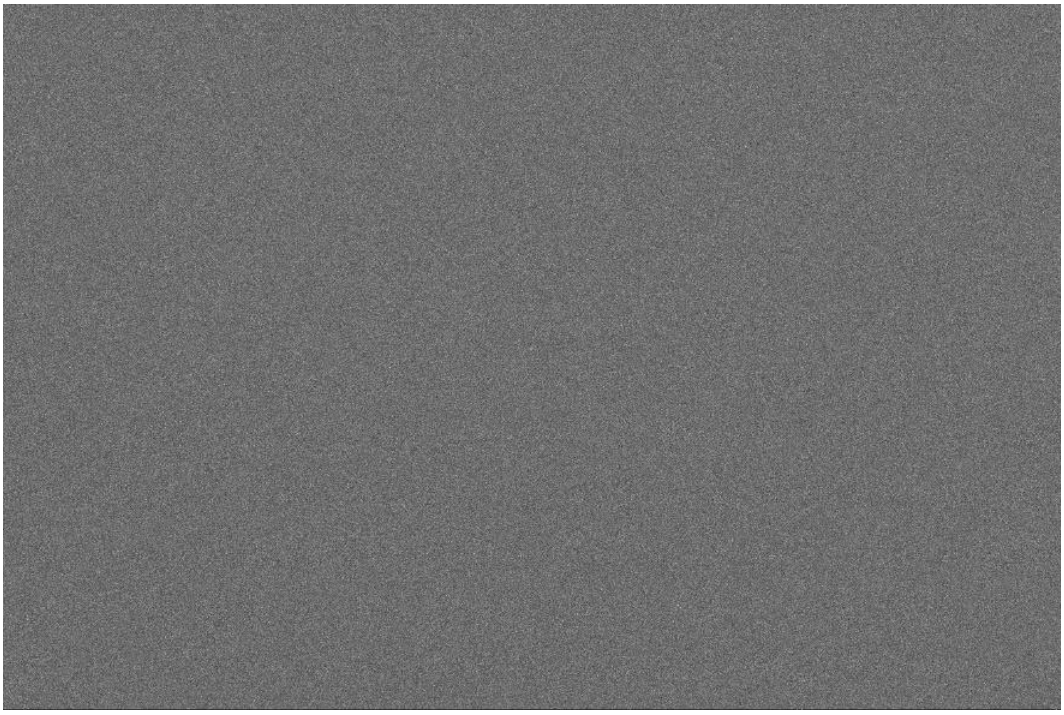

Figure S22. SEM image of the Au electrode surface after 20 minutes of CPE at 1.22 V vs. NHE in the presence of Cu(**L**). A magnification of 2500x, a high voltage of 15.00 kV and pressure of $2.32 \cdot 10^{-5}$ mbar was applied.

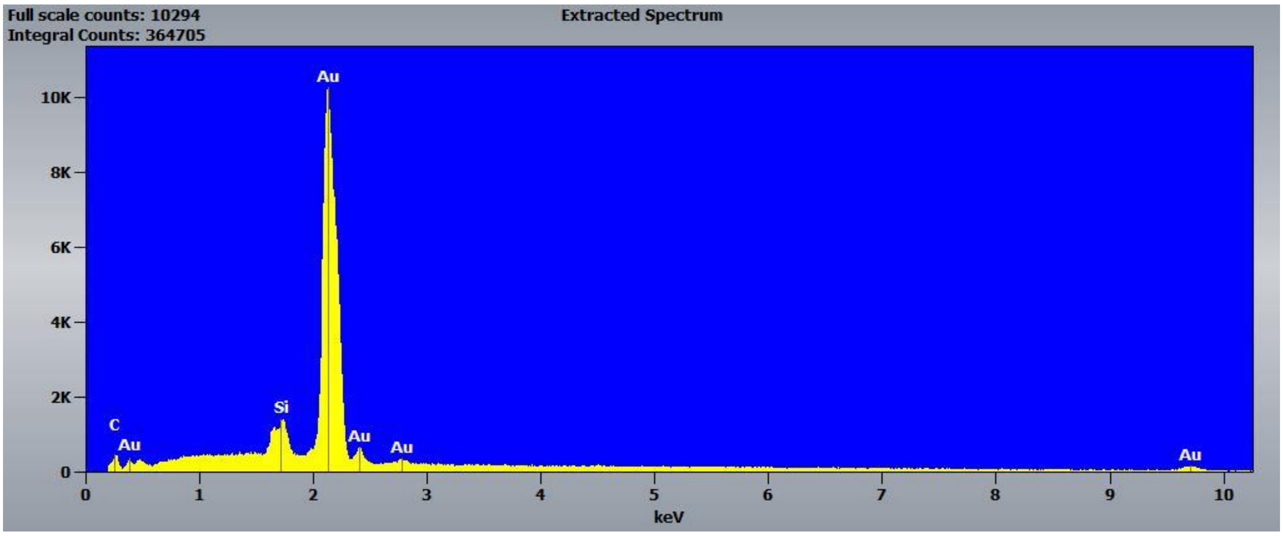

Figure S23. EDX spectra of Au electrode after 20 minutes of CPE at 1.22 V vs. NHE in phosphate buffer.

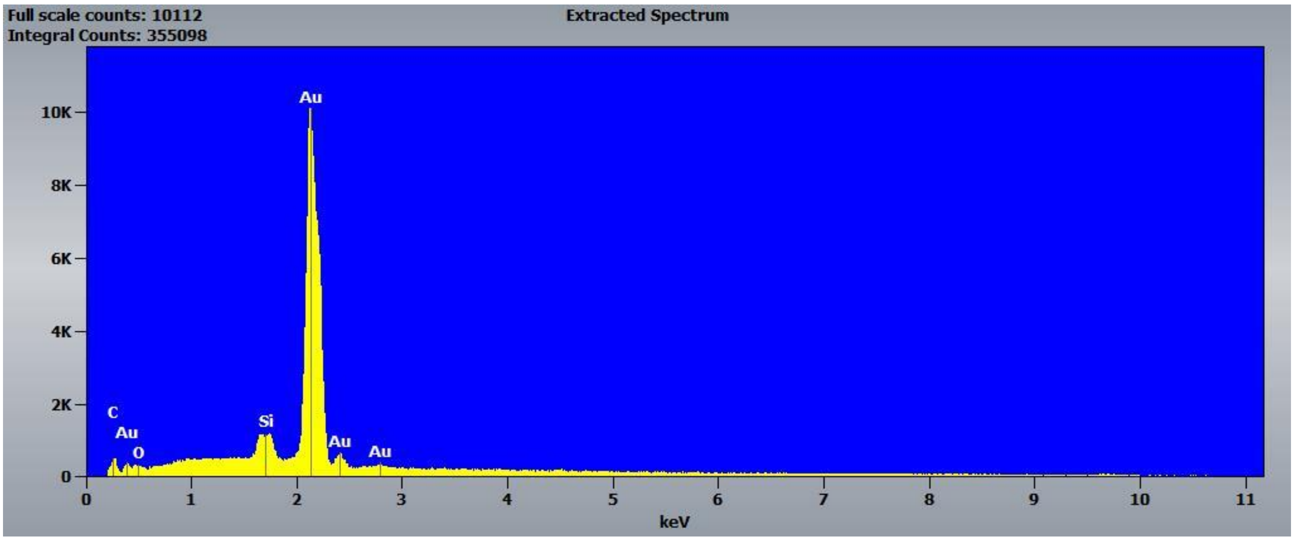

Figure S24. EDX spectra of Au electrode after 20 minutes of CPE at 1.22 V vs. NHE in the presence of Cu(**L**).

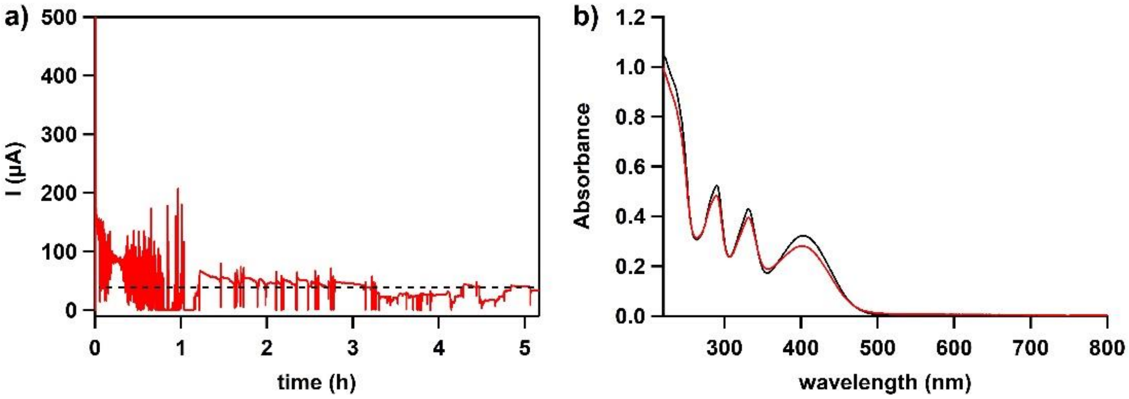

Figure S25. a) Chronoamperogram of 0.3 mM Cu(**L**) at 1.20 V vs. NHE in 100 mM pH 11.5 phosphate buffer. GC, Au and RHE were used as WE, CE and RE, respectively. Potentials were converted to (NHE). The signal noise is caused by the formation of bubbles on the electrode surface. The black dotted line shows average current. b) UV-vis spectra before (red) and after (black) 5 hours chronoamperometry at 1.20 V vs. NHE in 100 mM pH 11.5 phosphate buffer. Under these conditions sufficient current has been passed through the electrode to allow for a TON of 1.3.

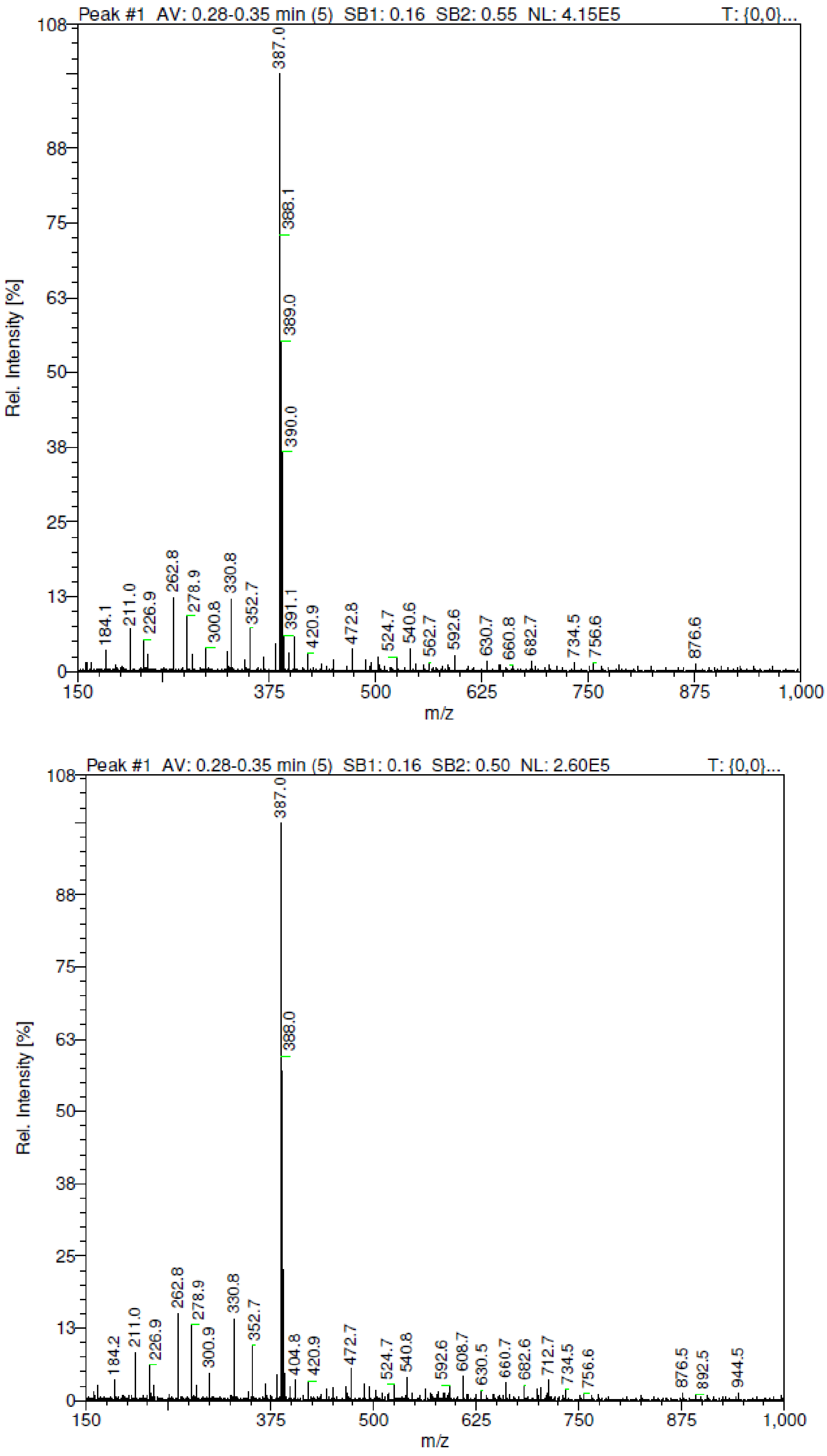

Figure S26. ESI-MS spectra of the electrolyte solution before (top) and after (bottom) 5 hours of chronoamperometry at 1.20 V vs. NHE of 0.3 mM Cu(**L**) 100 mM pH 11.5 phosphate buffer.

VIII. Kinetic Analysis

Calculation of k_{obs}

Throughout the kinetic studies, two different methods were used to calculate the reaction rate constants. In most CVs, catalytic plateau current were achieved indicating that in the kinetic regime the catalytic current was limited only by the chemical transformations involved in the actual WO catalysis. Consequently, the k_{obs} was estimated using the so-called the plateau method:[12]

$$\frac{i_{cat}}{i_p} = \frac{n}{0.4463} \cdot \sqrt{\frac{RT}{Fv} \cdot k_{obs}}$$

Here the k_{obs} is equal to TOF since the maximum catalytic activity was attained. It must be noted that in order to correctly apply this method, under current enhancement, the i_{cat}/i_p ratio must be equal to or greater than 4. After the rearrangement of the equation above, the observed rate constant was calculated as follows:

$$k_{obs} = 0.4848 \cdot v \cdot \left(\frac{i_{cat}}{i_p}\right)^2$$

For determination of the kinetic isotope effect the foot-of-the-wave analysis (FOWA) was applied.[13] The k_{obs} was extracted by plotting i_{cat}/i_p against the 1/1+exp[F($E_{½}$–E)/RT] term according to the equation written below:

$$\frac{i_{cat}}{i_p} = \frac{n \cdot 2.24 \cdot \sqrt{\frac{RT}{Fv} \cdot k_{obs}}}{1 + \exp\left[\frac{F}{RT} \cdot \left(E_{1/2} - E\right)\right]}$$

In these equations i_{cat} is the catalytic current in A, i_p is the current in absence of substrate, corresponding to the oxidative current in the Cu^{I}/Cu^{II} redox couple in A, k_{obs} is the reaction rate constant in s^{-1}, n is the number of electrons involved in the reaction (4 in the case of water oxidation), R is the universal gas constant of 8.314 $J \cdot mol^{-1} \cdot K^{-1}$, T is the temperature in K (298 K in this case), F is the Faraday constant of 96485 $C \cdot mol^{-1}$, v is the scan rate in $V \cdot s^{-1}$, E is the potential in the catalytic wave in V and $E_{1/2}$ is the potential at which half of the catalytic current is achieved in V.

Concentration dependence

CVs of varying concentrations of Cu(**L**) were obtained by replacing the catalyst solution with the corresponding phosphate buffer of 100 mM phosphate buffer (pH 11.3) to keep the phosphate concentration and ionic strength constant.

Overall baseline correction for the background current of the BDD electrode was determined by plotting the catalytic current versus the concentration resulting in an intercept corresponding to the current obtained after catalysis (see dipping test experiment). The overall background current at 1.22 V vs. NHE was subtracted from all currents at 1.22 V vs. NHE at all concentrations. To determine the kinetic order of the catalyst (x), the logarithm of the concentration ([Cu]) was plotted vs the logarithm of the corrected current (i_{cat}). The order in catalyst could be determined by the slope, which results in a slope of 1.0 indicating a first order dependence.

$$i_{cat} = [Cu]^x$$

$$\log i_{cat} = x \log[Cu]$$

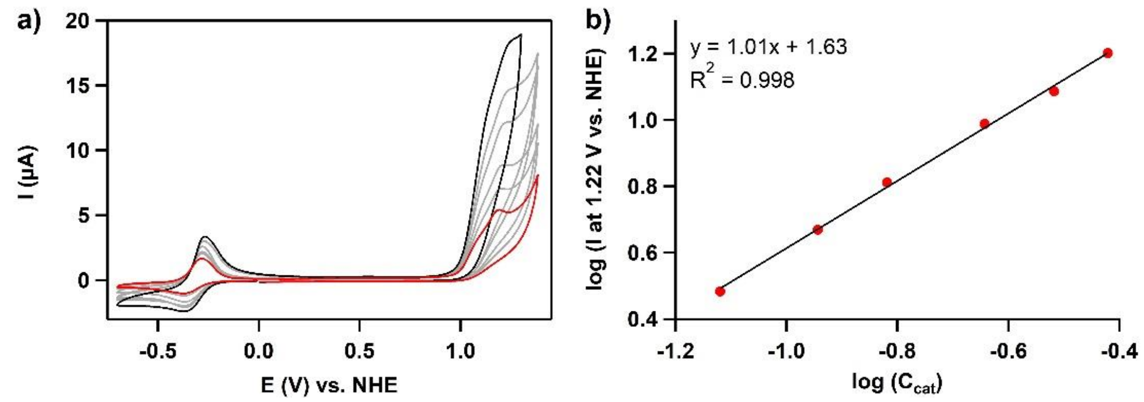

Figure 27. a) CVs at different concentrations of Cu(**L**) in 100 mM pH 11.3 phosphate buffer at a scan rate of 100 mV/s. BDD (0.07 cm^2), Au and RHE were used as WE, CE and RE, respectively. Reference potentials were converted to NHE. b) Logarithm of the concentration Cu(**L**) vs. logarithm of the current at 1.22 V vs. NHE, showing a 1st-order dependence on Cu(**L**).

As a positive control in this experiment the UV-vis spectra of every concentration was recorded. All samples were diluted 10x before the spectra were recorded (Figure S28). Plotting the absorbance at 404 nm vs the catalyst concentration results in a linear correlation, indicating the solutions were diluted correctly and the solution was stable during the experiment.

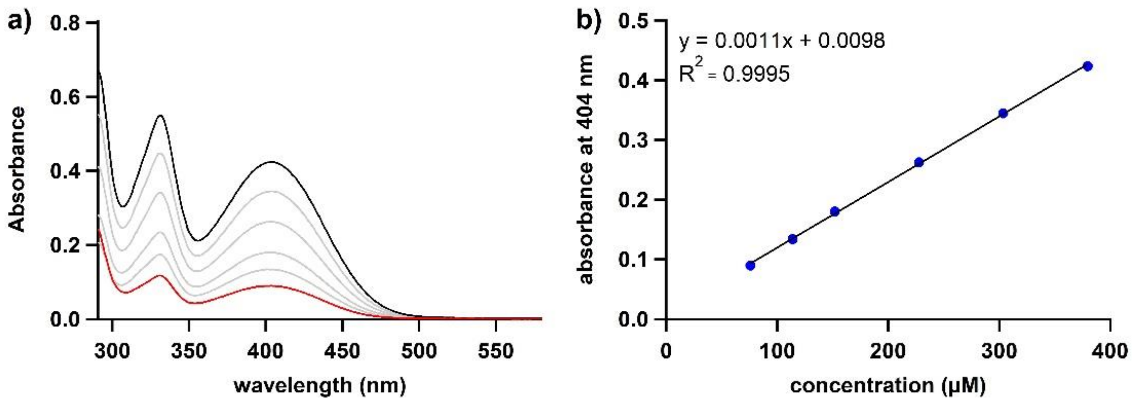

Figure S28. a) UV-vis spectra at varying concentrations of Cu(**L**) in 100 mM pH 11.3 phosphate buffer. b) Linear dependence of the absorbance at 404 nm on the Cu(**L**) concentration.

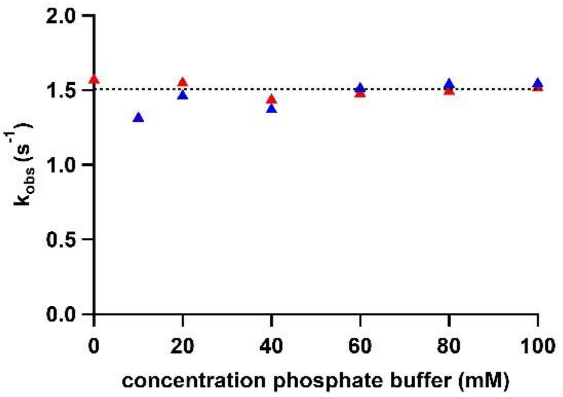

Figure S29. Plot of k_{obs} of Cu(**L**) as a function of phosphate concentration. (Blue and red data points correspond to data points obtained at pH values of 11.6 and 11.2, respectively). Data were obtained from cyclic voltammetry experiments of 0.3 mM Cu(**L**) in 100 mM electrolyte solutions (mixtures of phosphate and sulfate) at a scan rate of 100 mV/s. BDD (0.79 cm^2), Au and RHE were used as WE, CE and RE, respectively.

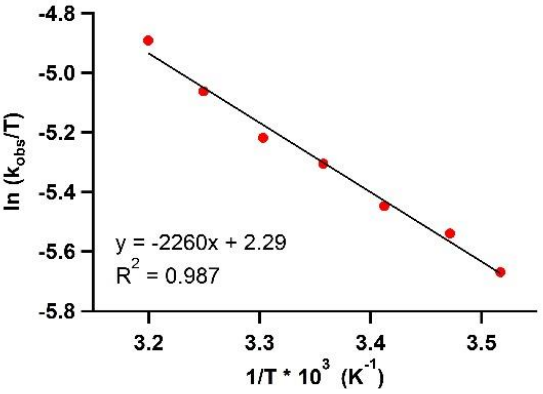

Figure S30. Eyring plot of the catalytic WO wave of Cu(**L**). Data were obtained by cyclic voltammetry experiments of 0.3 mM Cu(**L**) in 100 mM pH 11.6 phosphate buffer at a scan rate of 100 mV/s at temperatures varying between 283 K (10 °C) and 313 K (40 °C). BDD (0.07 cm^2), Au and RHE were used as WE, CE and RE, respectively.

IX. Computational study

All DFT calculations were carried out using the Gaussian16 (Revision B.01) suite of programs.[14] The geometries were fully optimized without any constrains with the PBE0 (PBE1PBE) functional[15] augmented by Grimme's dispersion correction with Becke-Johnson damping, GD3(BJ).[16] A superfine integration grid was utilized throughout the whole studies. Solvent effects were taken into account by means of a hybrid cluster-continuum approach[17] in which the first coordination sphere was described explicitly with a limited number of water molecules (1-4) and the continuum part was modeled by the polarizable conductor calculation (C-PCM) method[18] with water as a solvent (ε = 78.3553) and non-default UAKS atomic radii (as optimized for the PBE0/6-31G(d) level of theory). The Cu atom was described with Stuttgart RECP and associated basis set.[19] For all other atoms, standard full electron Pople's basis set 6-31+G(d,p) was used.[20] This basis set is denoted as BS1. All computed structures were characterized as local stationary points with no imaginary frequencies via analytical frequency calculations.

Free energy corrections were calculated at the standard state (298.15 K, 1 atm). Grimme-type quasi-harmonic correction[21] (cut-off value 100 cm^{-1}) was performed as implemented in the GoodVibes script.[22] Zero-point vibrational energy (ZPPVE) scaling factor 0.9771 was employed (PBE0/6-31+G(d,p) in order to "mitigate" systematic errors (e.g. poor description of electron-electron correlation and neglect of anharmonicity) in the calculated harmonic vibrational frequencies.[23] An additional correction term of +1.9 kcal/mol (at 298 K) was added to account for the standard state concentration of 1 M for all optimized structures except water, for which the standard state corresponds to 55.6 M (+4.3 kcal/mol).

Additional single-point calculations based on PBE0/BS1 optimized geometries were performed with the PW6B95D3 functional[24] and a larger basis set combination denoted as BS2. This includes the full electron def2-QZVP basis set for Cu obtained from the Basis Set Exchange repository (BSE)[v] and Pople's 6-311+G(2d,p) basis set[25] for the remaining atoms. This computational protocol was benchmarked against various redox couples (Table B5) and found to provide the most satisfactory results compared to other functionals. The PW6B95D3/BS2 single-point energies modified by scaled Gibbs free energy correction from the PBE0/BS1 calculations were used to describe the reaction energies throughout the study.

For water, the experimentally established hydration energy (–6.3 kcal/mol) was added to gas-phased single point energy.[26] Given the difficulties associated with accurate determination of the hydration energy of the hydroxide, the following "computational" OH^- anion was devised in order to determine the free energy of the latter:

$$G_{aq}(OH^-) = G(H_2O) - G(H^+) - \Delta G^{\circ}_{eq} + \Delta G_f\,,$$

where $G(H_2O)$ – free energy of water defined as mentioned above, $G(H^+)$ – free energy of the proton in aqueous solution (–11.72 eV, pH=0),[27] ΔG_f – free energy which corresponds to ionic product of water ($1 \cdot 10^{-14}$ at 298K), ΔG_f – extra term of +2.2 kcal/mol to account for the energy required to form the hydroxide from water taking into account the difference in pK_a values between water (14.0) and hydrophosphate (12.37) presented in abundance in the buffered solution at pH 11.6:

[v] https://www.basissetexchange.org/

$$H_2O + PO_4^{3-} \rightleftharpoons OH^- + HPO_4^{2-}$$

14.0 **12.37**

All theoretical redox potentials were calculated relative to the normal hydrogen electrode (NHE) at pH 11.6 using the following generic equation:

$$E^{\circ}_{NHE}(red|ox) = -\frac{\Delta G^{\circ}_{red|ox}}{n_e F} - E^{\circ}_{abs}(SHE) - E^{\circ}_{SHE|NHE} - 2.303RT\frac{n_p}{n_e} \times pH,$$

where $E^{\circ}_{abs}(SHE)$ – absolute hydrogen potential (+4.281V),[28] $E^{\circ}_{SHE|NHE}$ - SHE-to-NHE correction factor (+0.006 V),[29] n_p and n_e – number of transferred protons and electrons, respectively.

The O-H bond dissociated free energy (BDFE) used to construct the thermodynamic cycle (see Figure S1) was calculated using the Bordwell equation:[30]

$$BDFE(O - H) = 1.37pK_a + 23.06E^{\circ}_{NHE} + 57.6\ kcal/mol$$

The pK_a values were calculated as:

$$pK_a = \frac{\Delta G^{\circ}}{2.303RT} \pm \log[H_2O]^n$$

where n stands for number of explicit molecules of water either added or left from the cluster upon deprotonation.

Localized orbital bonding (LOBA)[31] and Reduced density gradient (RDG)[32] analysis were performed with MultiWFN program[33] using the PW6B95D3/BS2-based wavefunctions. ETS-NOCV energy decomposition analysis[34-35] was carried out as implemented in the ADF 2019.3 program.[36]

The barrier of the inner-sphere electron transfer was estimated with the assistance of Marcus equation:[37]

$$\Delta G^{\neq} = \frac{(\lambda_i + \Delta G^{\circ})^2}{4\lambda_i},$$

Where ΔG° – the total Gibbs free energy change for the electron transfer and λ_i – reorganization energy which, in turn, was approximated via the corresponding excitation energy. ωB97xD and CAM-B3LYP functionals and corrected linear response[38] and state-specific[39] algorithms were used.

Molecular modeling and geometry visualization were performed using the ChemCraft program.[vi]

[vi] Zhurko, G. A. ChemCraft 1.8, http://www.chemcraftprog.com

Table S5. DFT benchmarking – computed redox potentials for Cu(H**L**) system.

Functional	Cu(I/II)	Cu^{II}(**L**)/Cu^{II}(**L**$^\bullet$)	Cu(**L**$^\bullet$)(–O)/Cu(**L**$^\bullet$)(–O$^\bullet$)
PBE0-D3BJ	-0.647	0.995	1.124
revPBE0-NL	-	0.913	1.008
TPSSh-D3BJ	-0.384	0.834	1.017
TPSS0-D3BJ	-0.644	0.989	1.095
PW6B95D3	**-0.366**	**1.016**	**1.179**
SCAN0-D3BJ	-	0.733	1.722
ωB97X-V	-0.555	1.090	1.196
ωB97X-D3BJ	-0.411	1.223	1.316
ωB97M-V	-0.633	0.978	1.087
ωB97M-D3BJ	-0.552	1.087	1.199
Experimental	-0.3	1.04	1.16

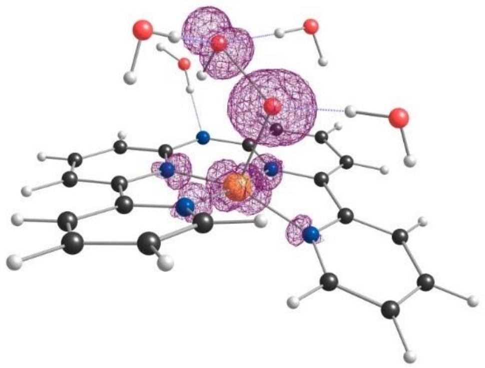

Figure S31. Spin density distribution in [Cu(**L**)(O···OH)] x $4H_2O$ spin density (surface isovalue 0.01 a.u.) with unpaired electron density on the oxygen atoms and Cu^{II}.

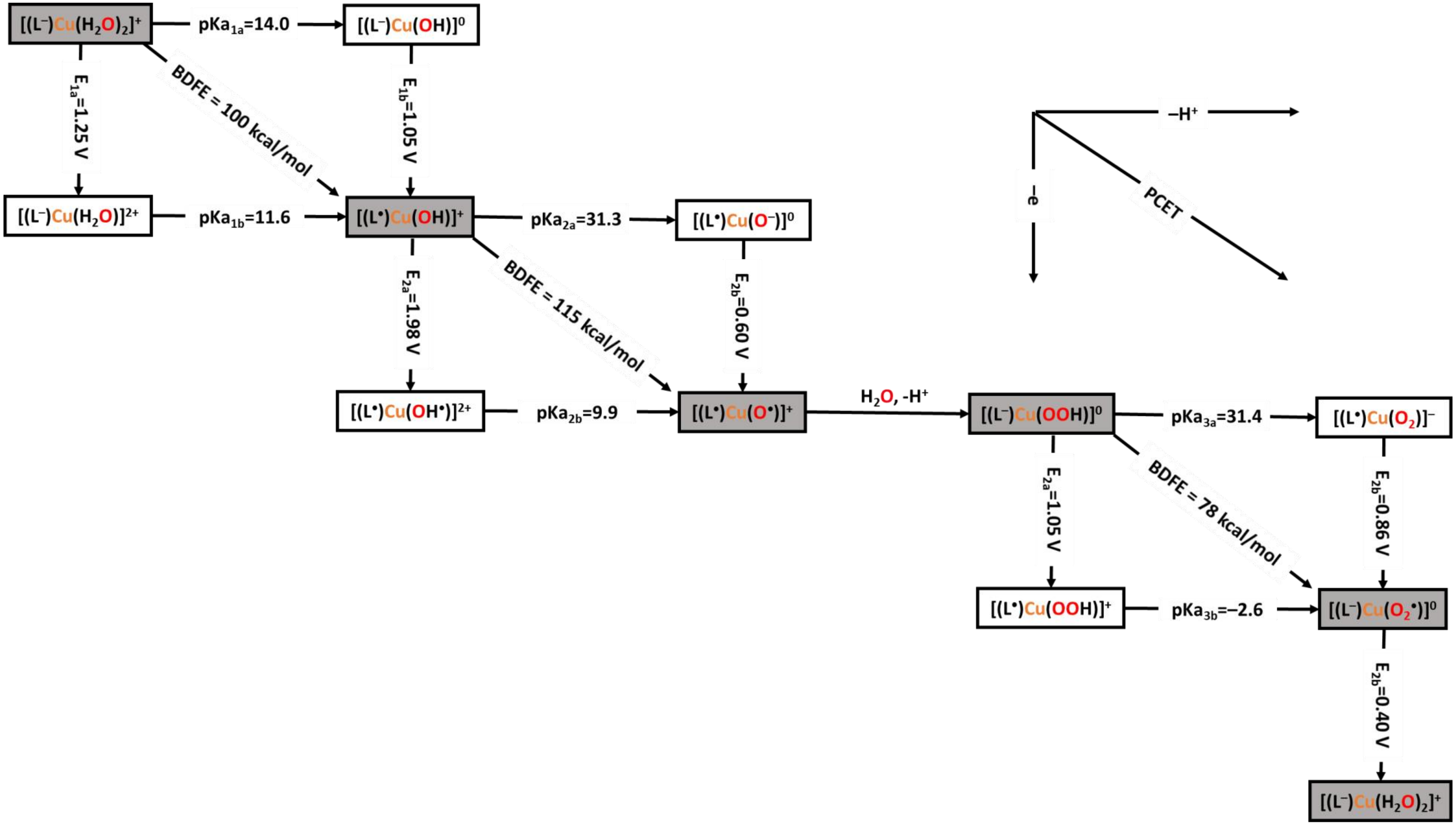

Figure S32. Bordwell thermodynamic square calculated for proposed WO mechanism.

X. NMR spectra

1H NMR — [2,2'-bipyridine]-1-oxide — 400 MHz CDCl3

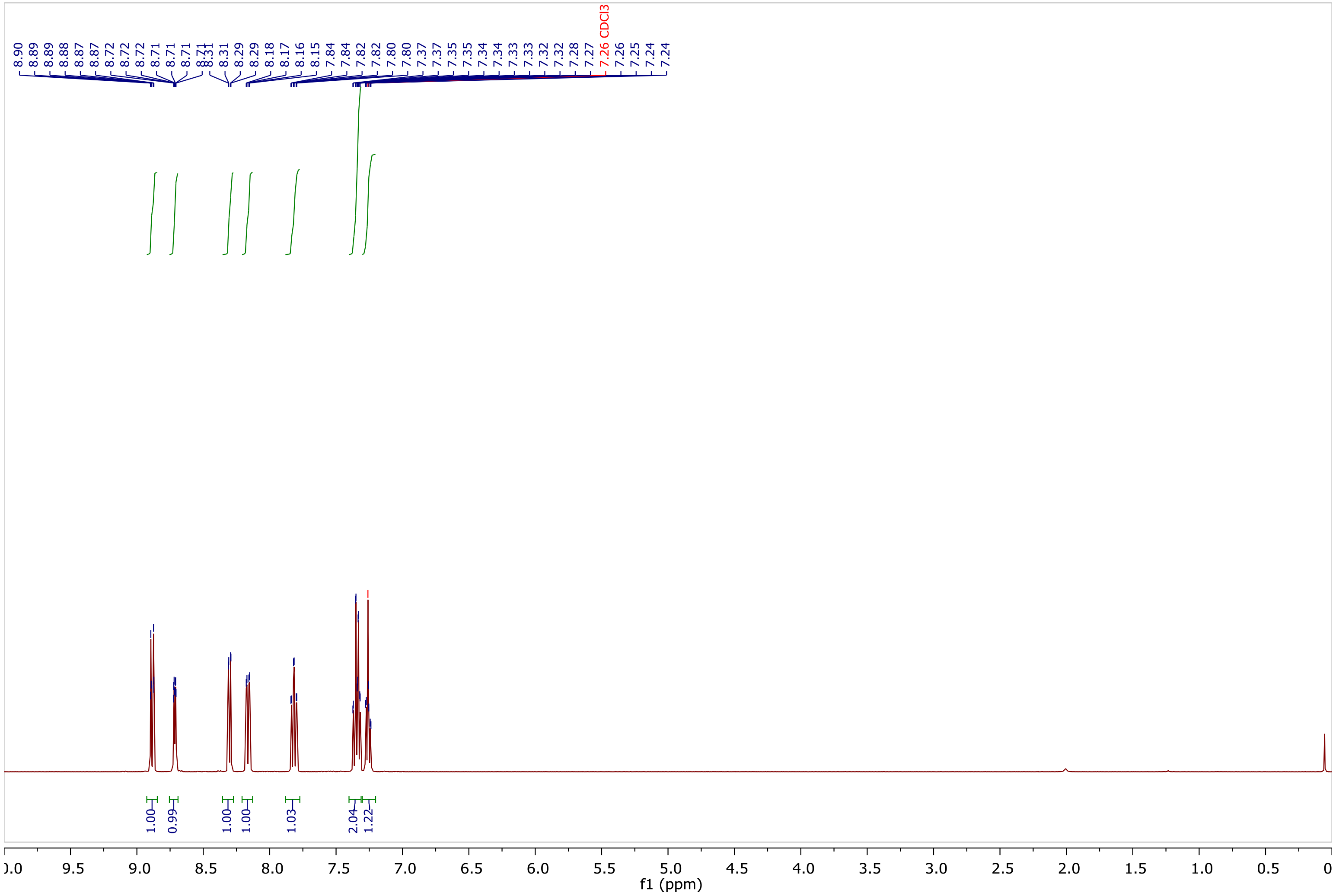

13C APT NMR — [2,2'-bipyridine]-1-oxide — 400 MHz CDCl3

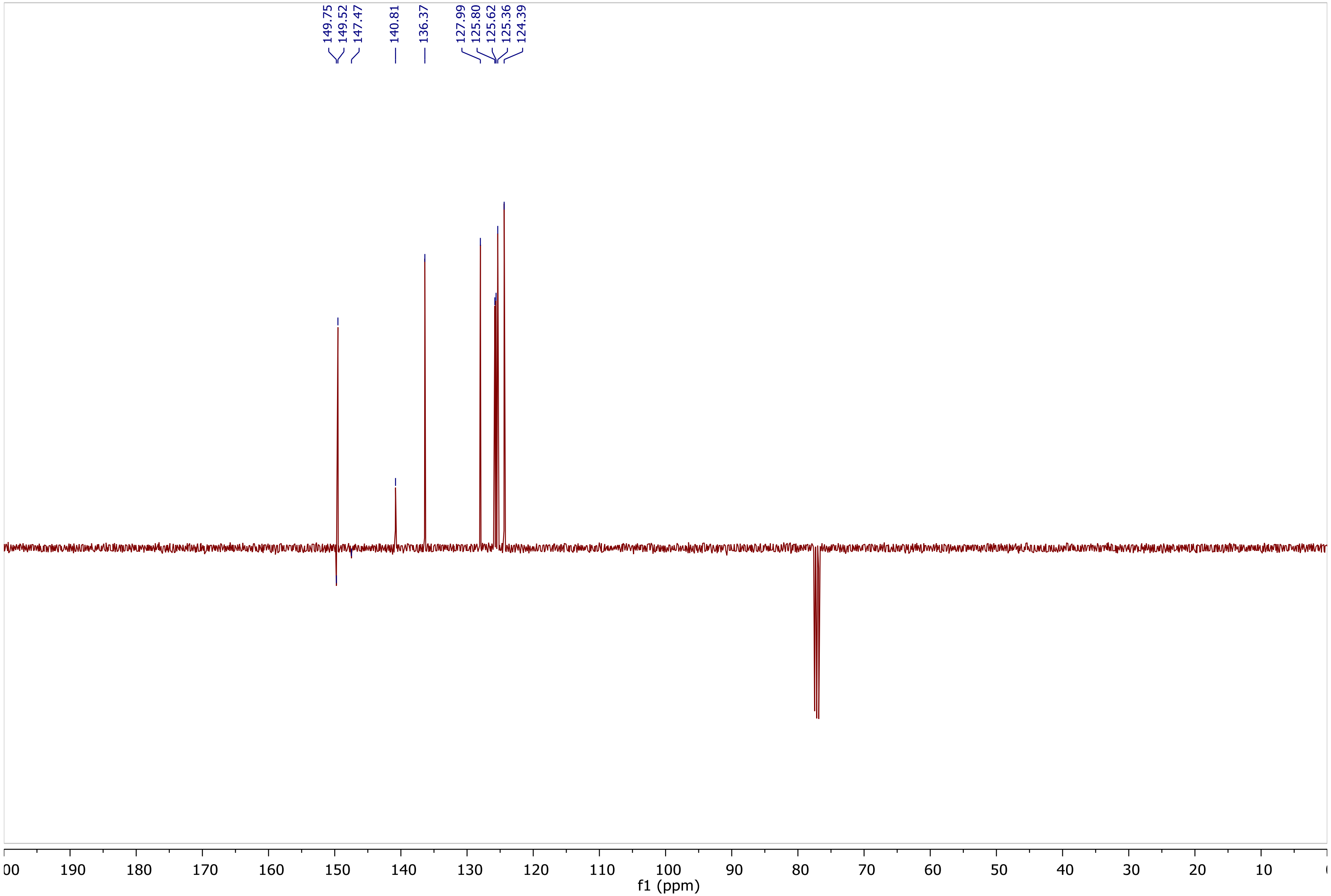

1H COSY NMR — [2,2'-bipyridine]-1-oxide — 400 MHz CDCl3

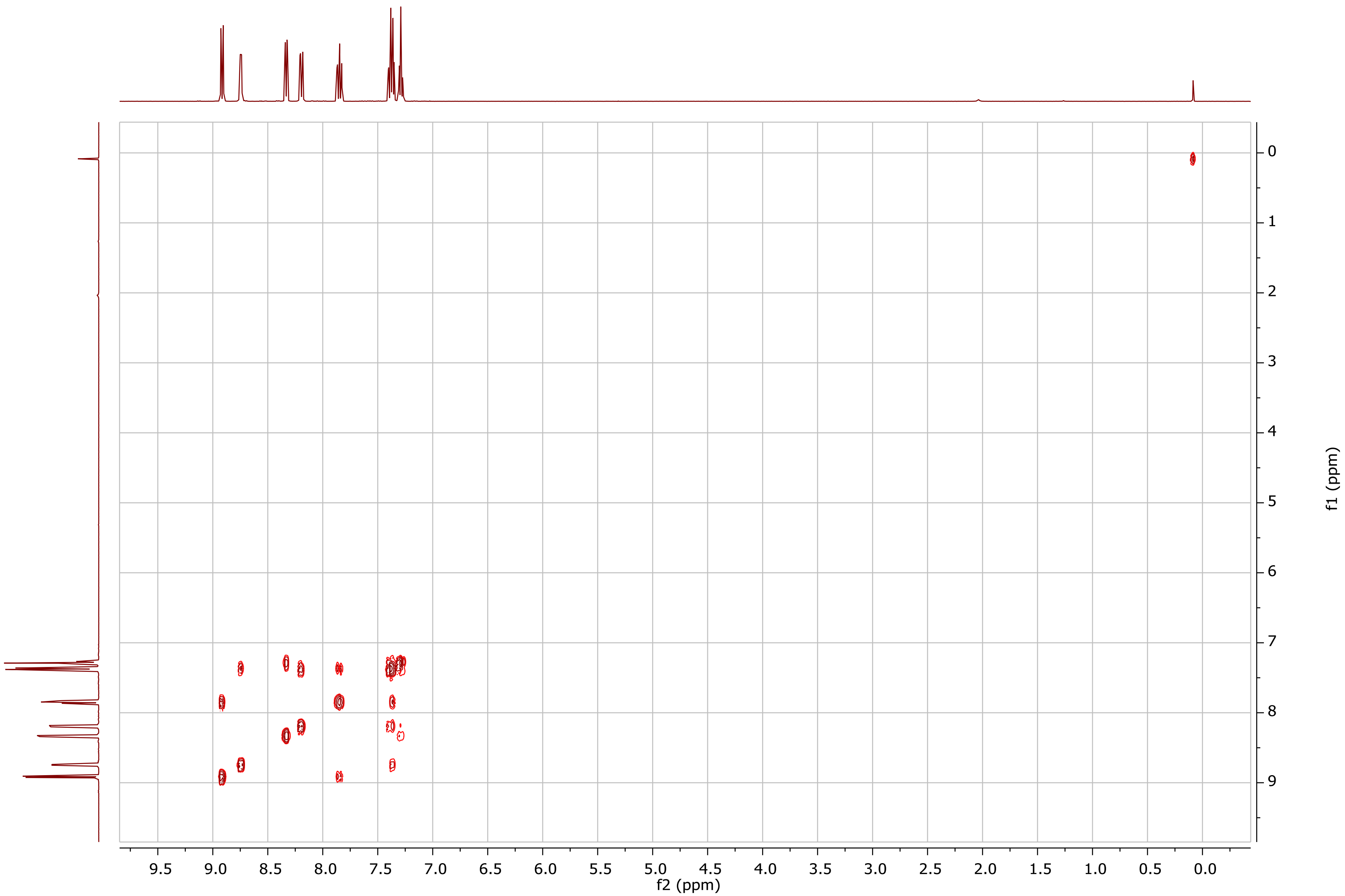

1H13C HSQC NMR — [2,2'-bipyridine]-1-oxide — 400 MHz CDCl3

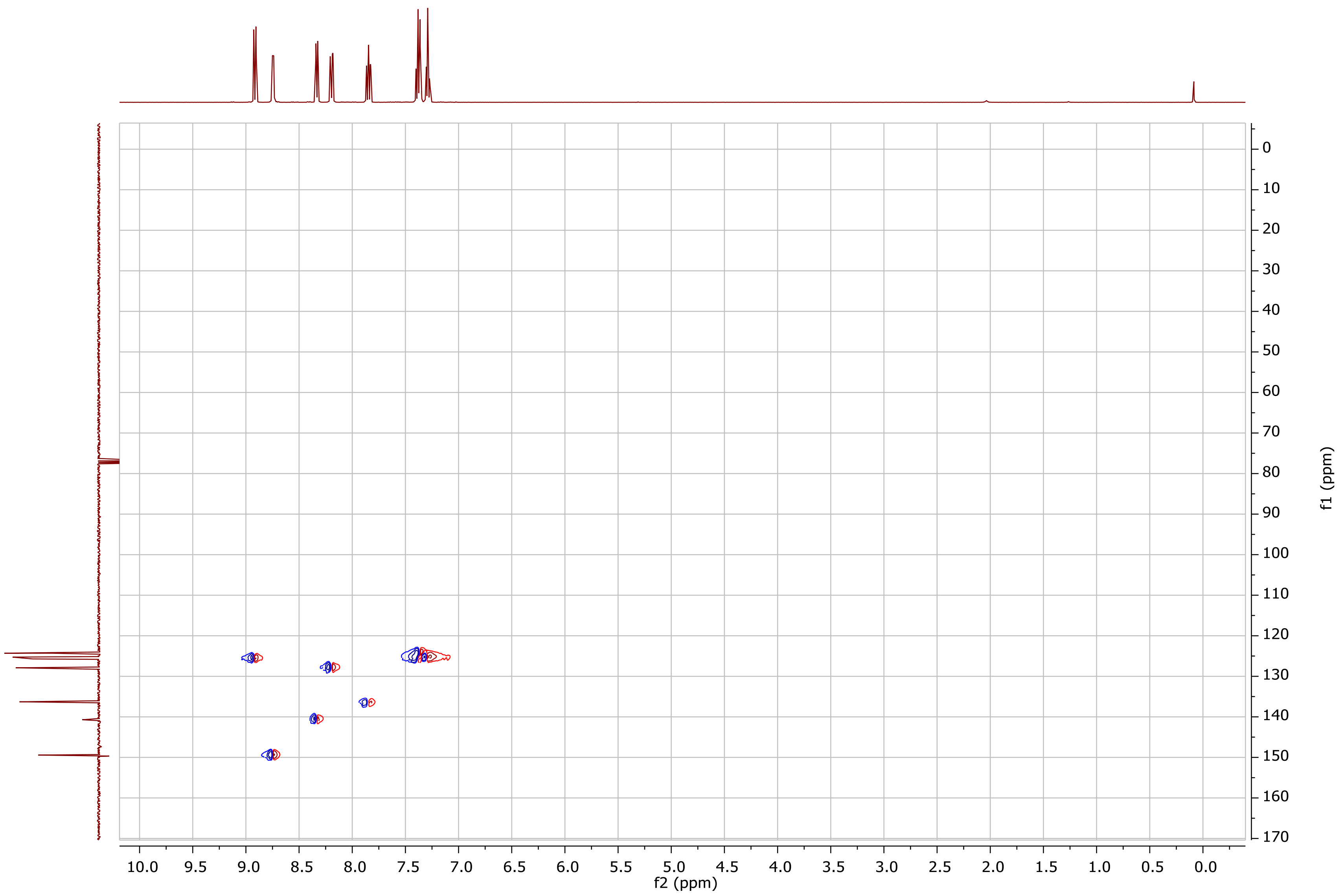

1H13C HMBC — [2,2'-bipyridine]-1-oxide — 400 MHz CDCl3

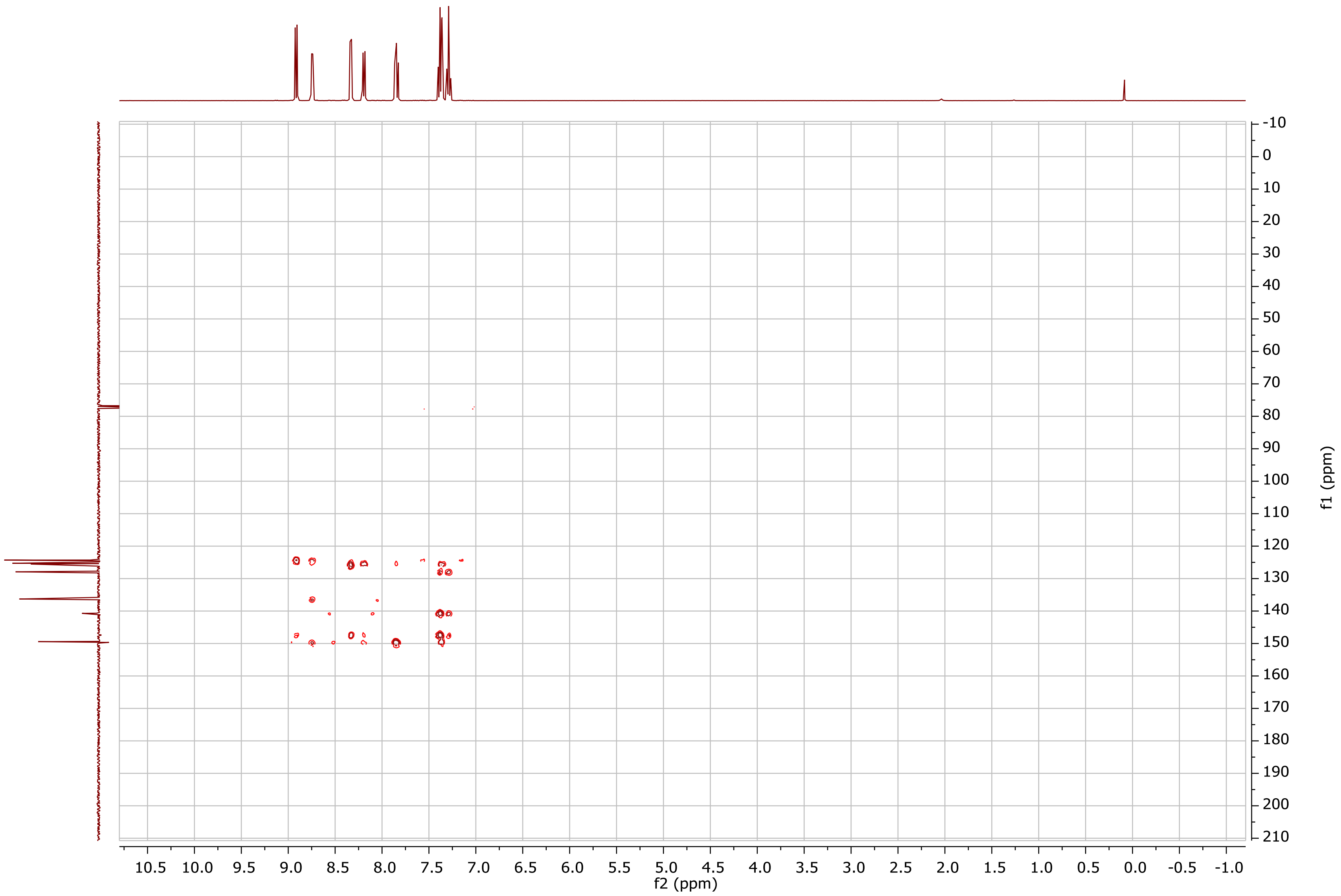

1H NMR — [2,2'-bipyridin]-6-amine — 400 MHz CDCl3

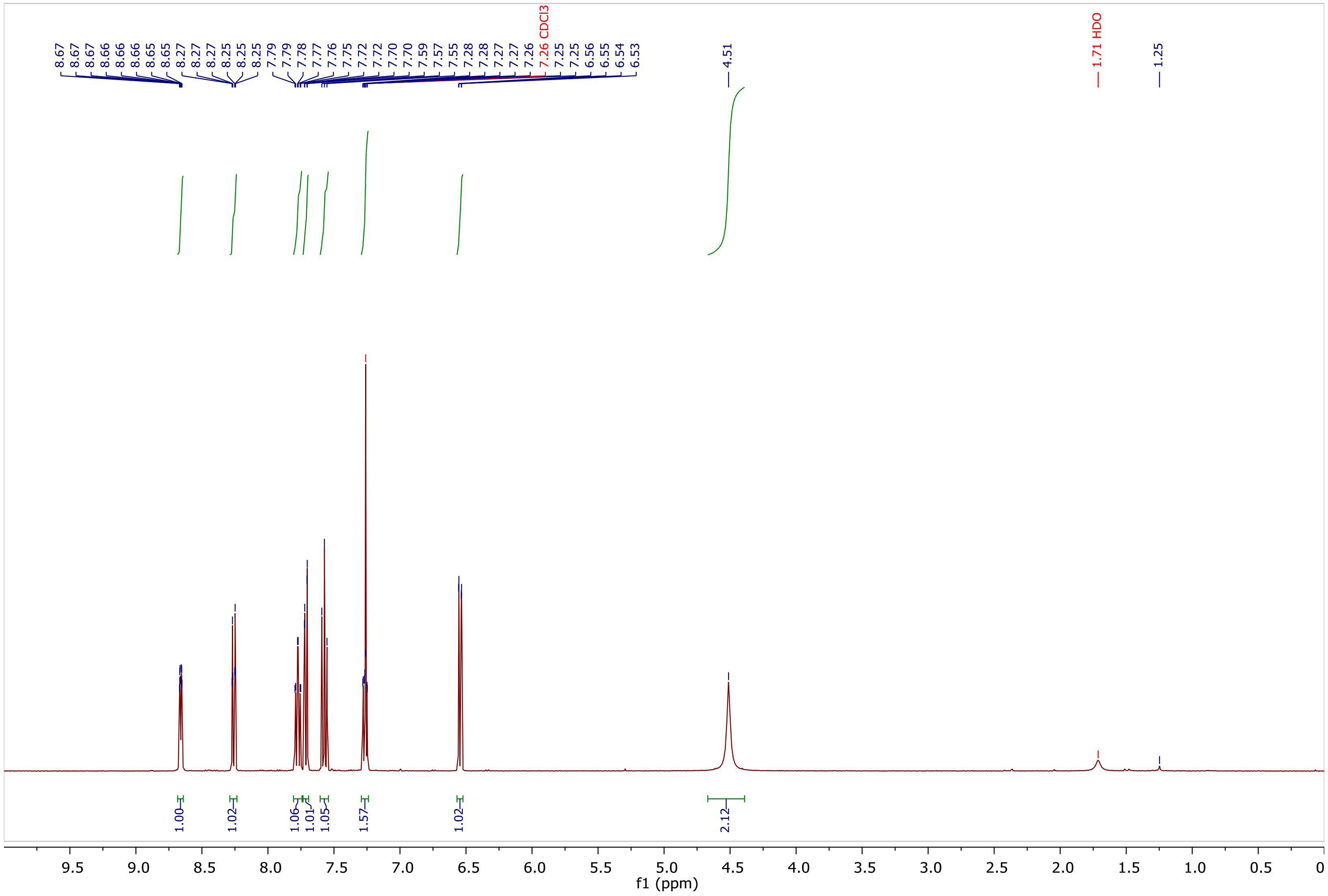

13C APT NMR — [2,2'-bipyridin]-6-amine — 400 MHz CDCl3

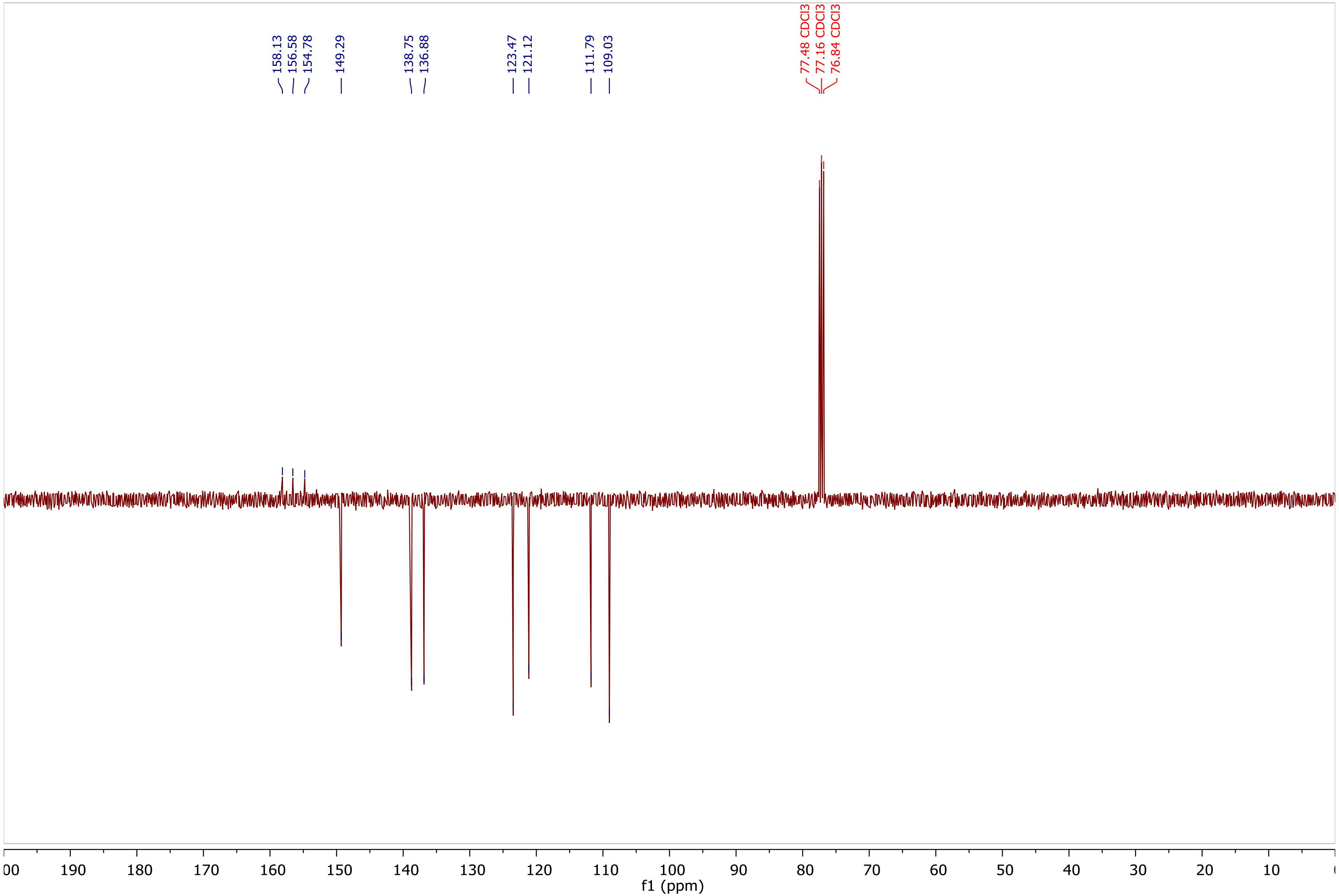

1H COSY NMR — [2,2'-bipyridin]-6-amine — 400 MHz CDCl3

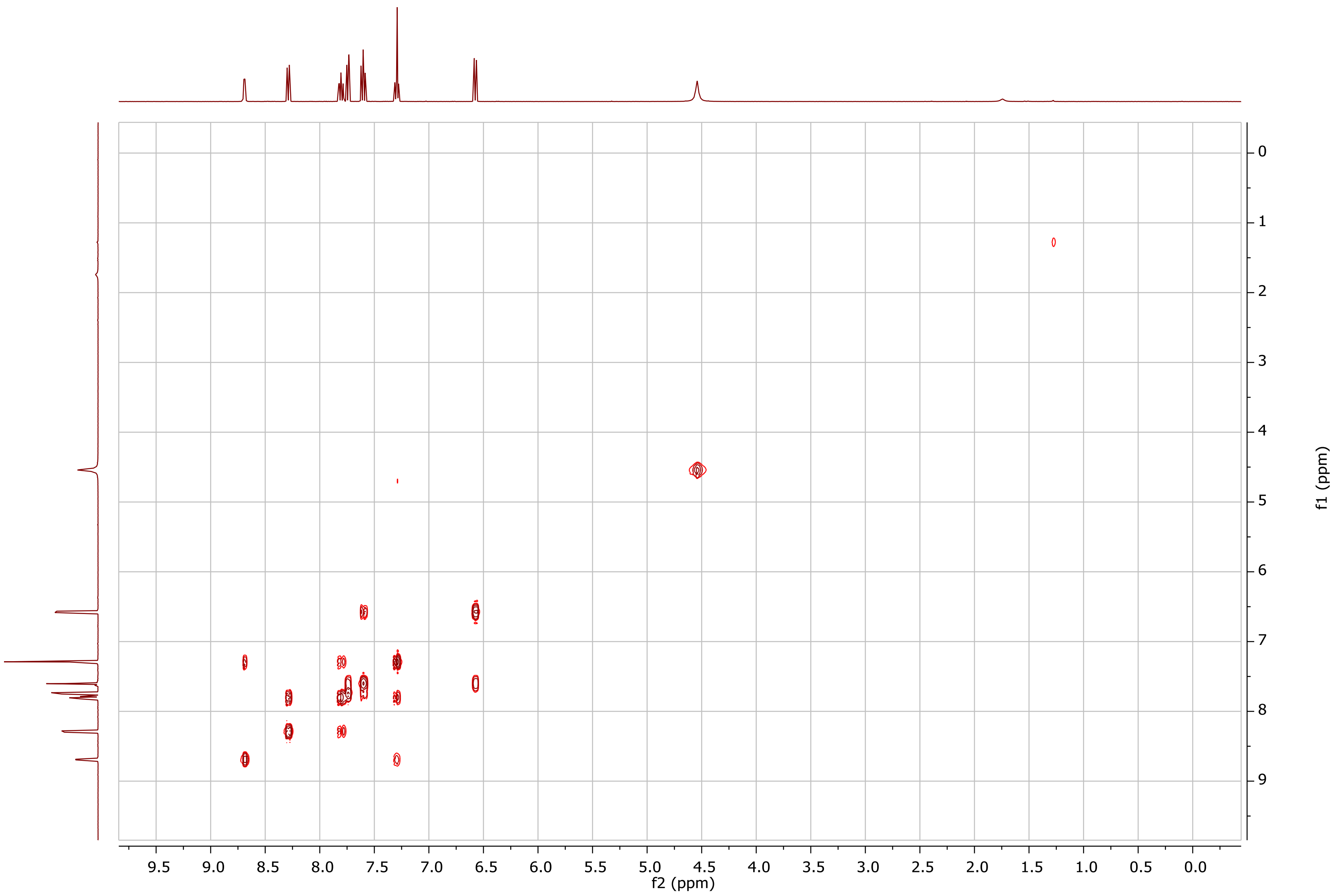

1H13C HSQC NMR — [2,2'-bipyridin]-6-amine — 400 MHz CDCl3

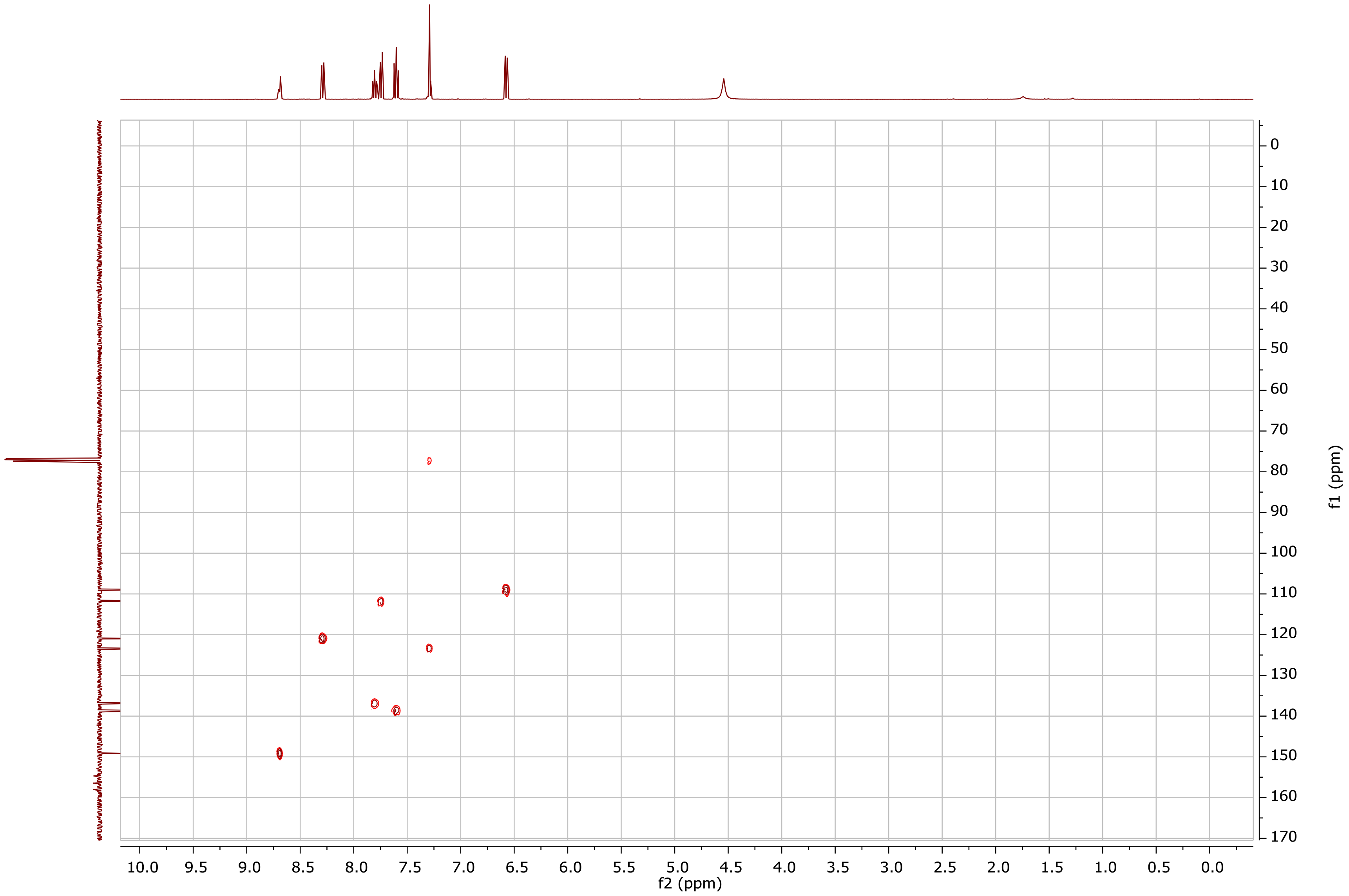

1H13C HMBC NMR — [2,2'-bipyridin]-6-amine — 400 MHz CDCl3

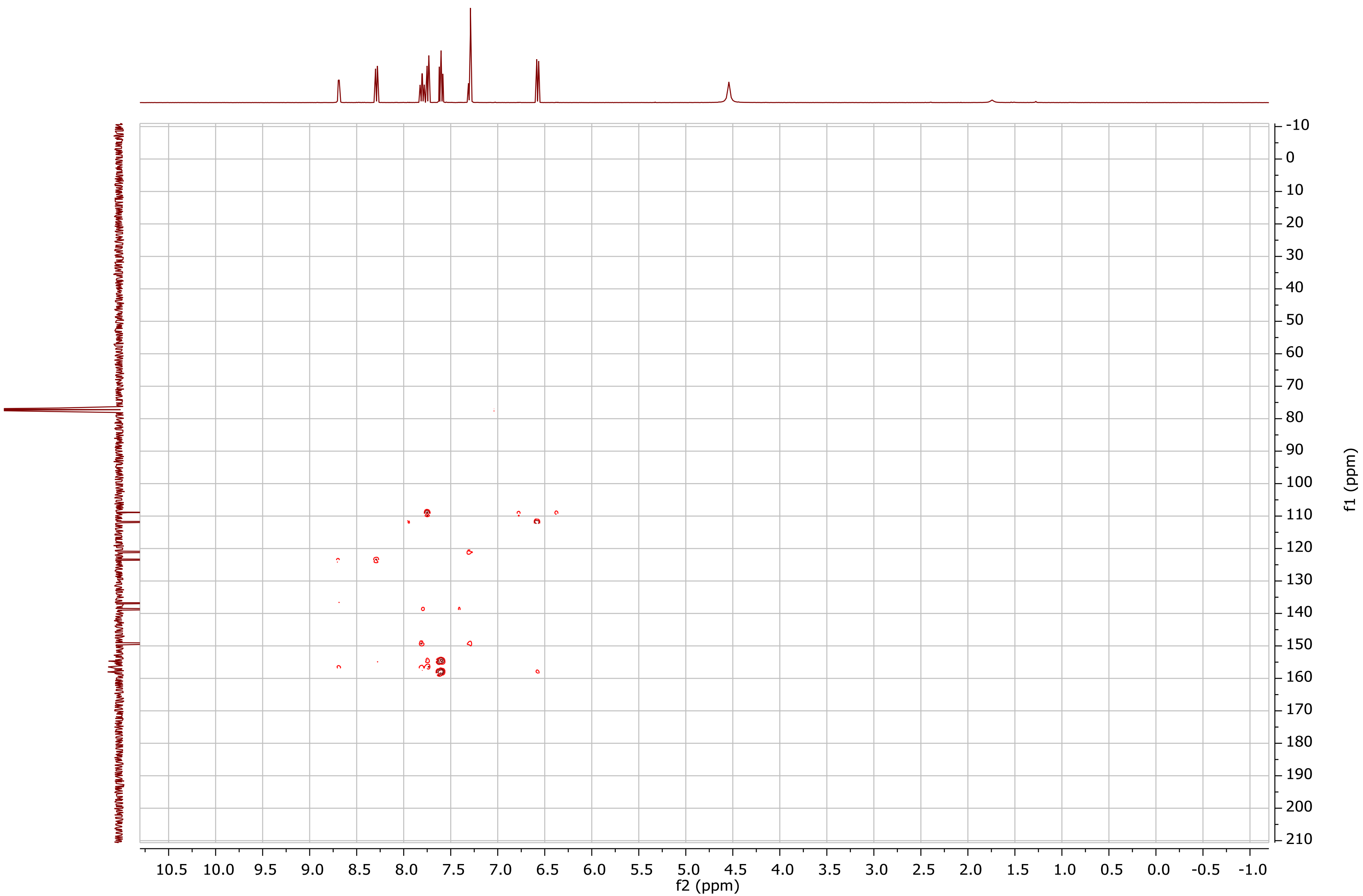

1H NMR — N,N-bis(2,2′-bipyrid-6-yl)amine) — 400 MHz CDCl3

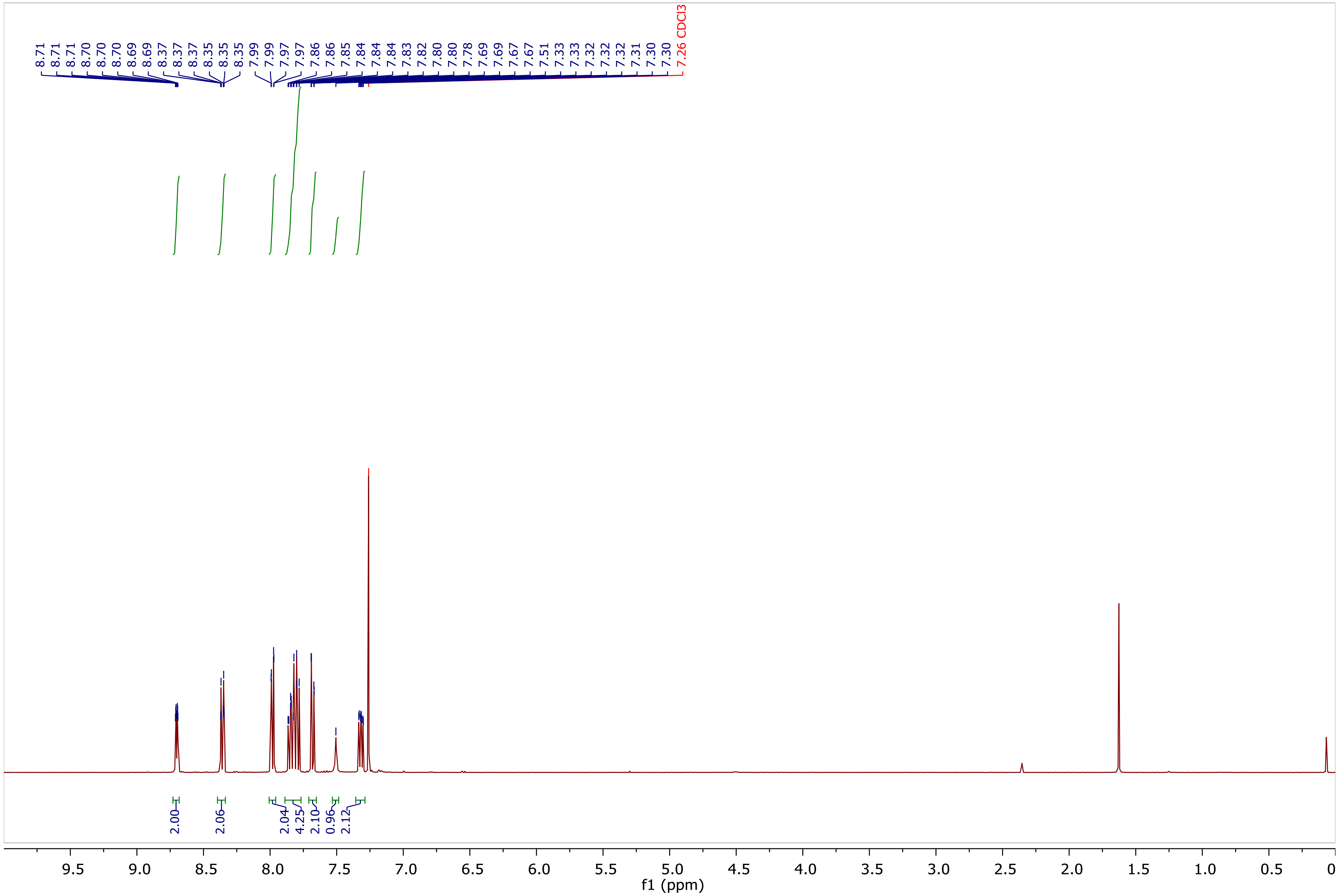

13C APT NMR — N,N-bis(2,2′-bipyrid-6-yl)amine) — 400 MHz CDCl3

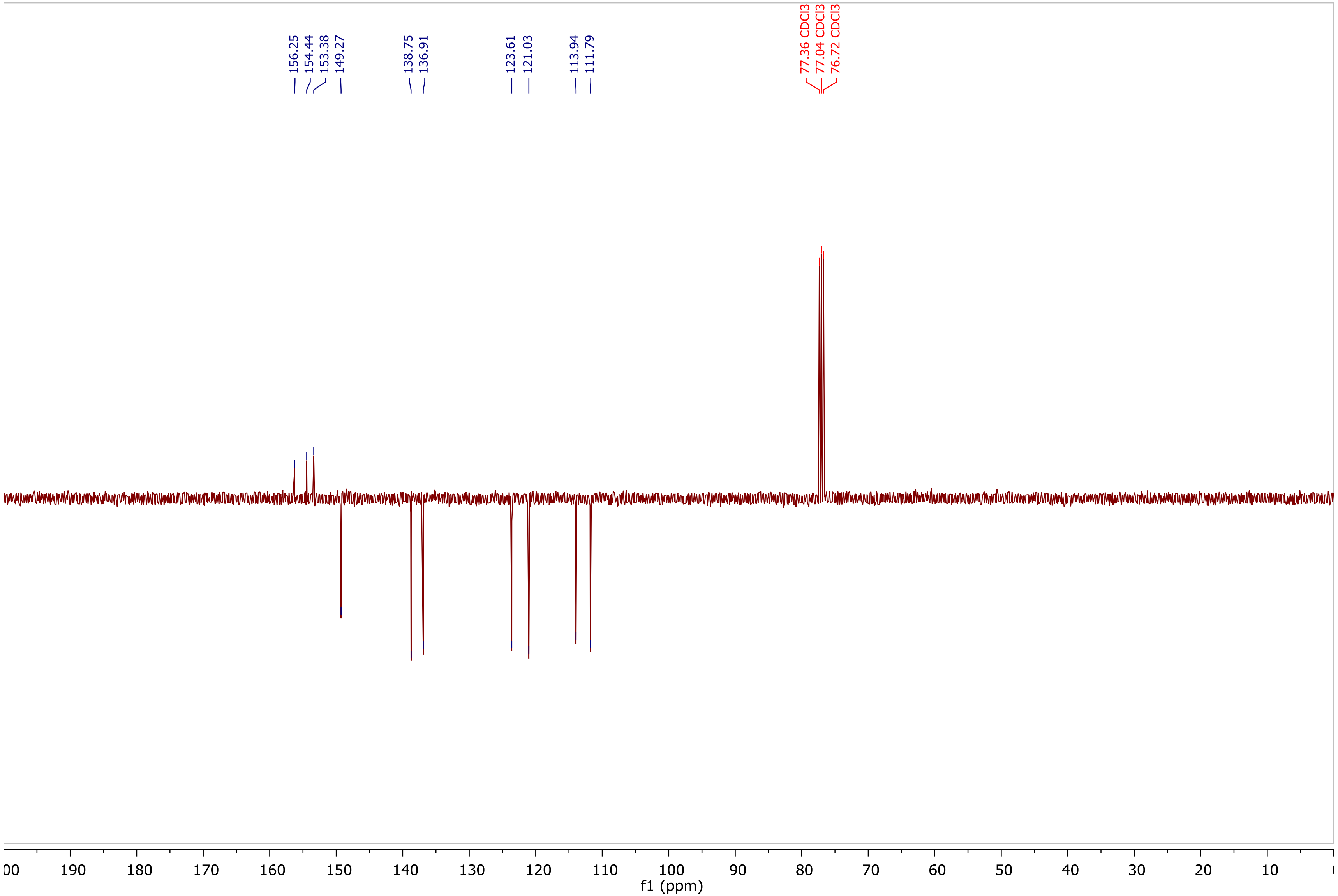

1H COSY NMR — N,N-bis(2,2'-bipyrid-6-yl)amine) — 400 MHz CDCl3

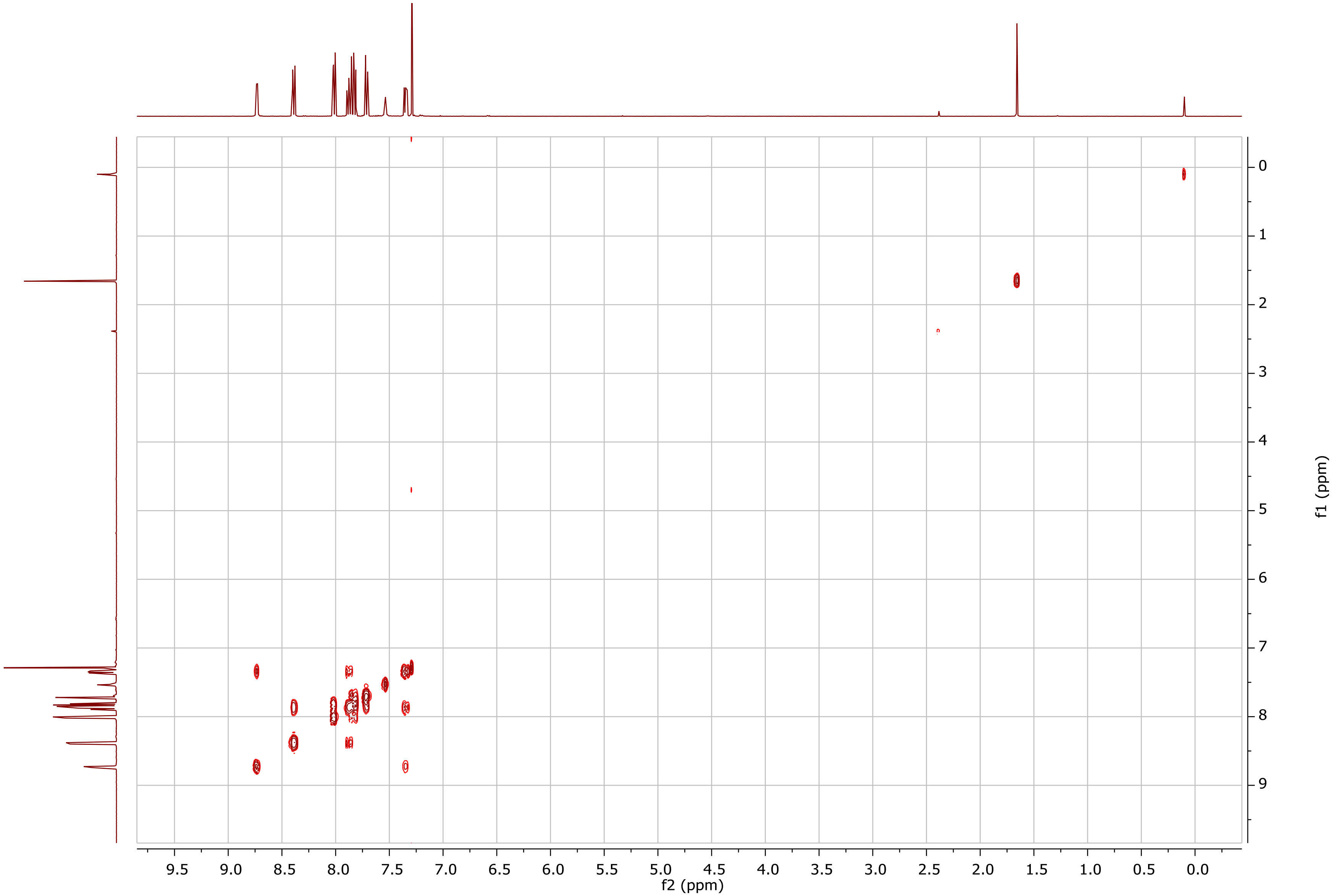

1H13C HSQC NMR — N,N-bis(2,2′-bipyrid-6-yl)amine) — 400 MHz CDCl3

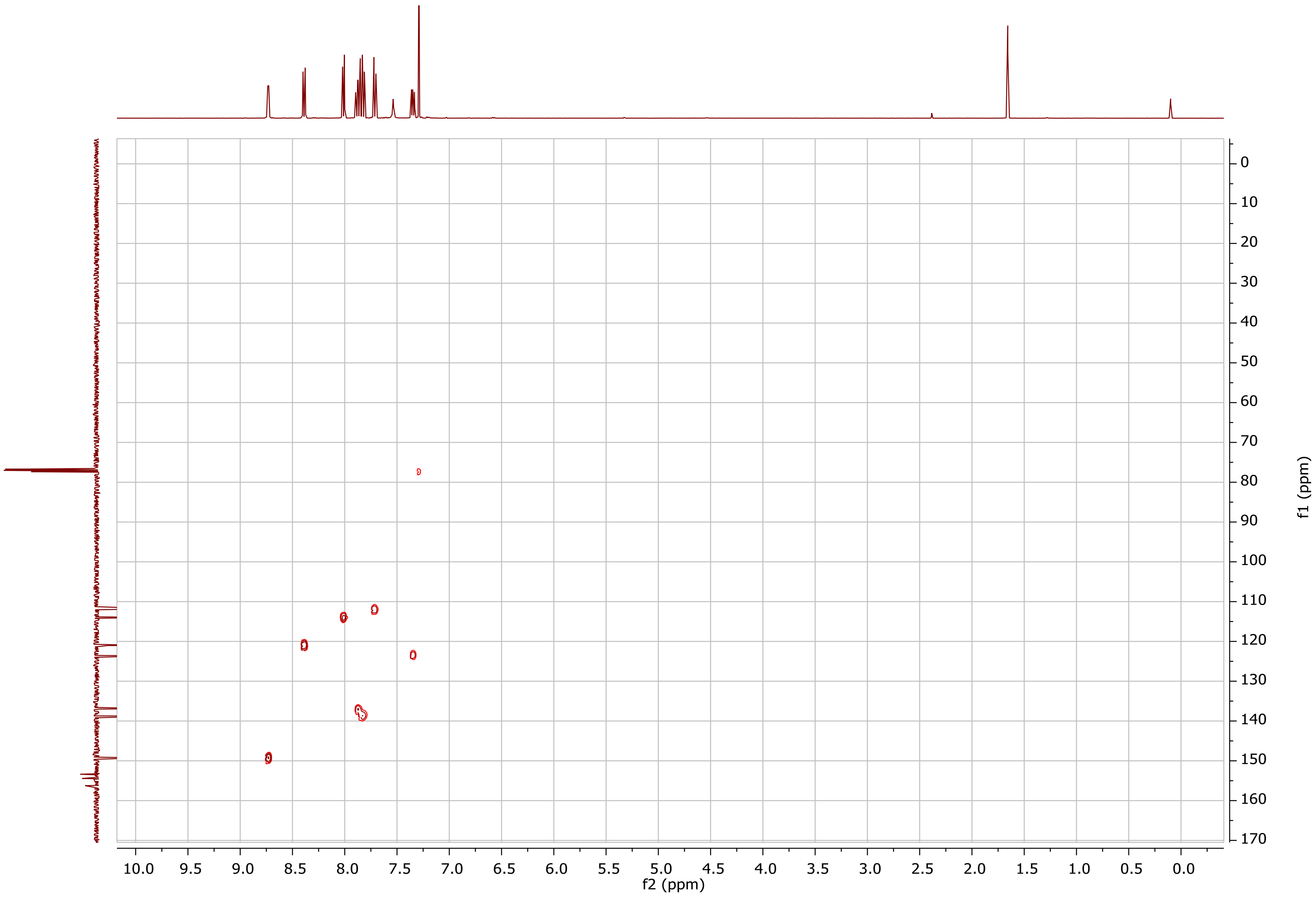

1H13C HMBC NMR — N,N-bis(2,2’-bipyrid-6-yl)amine) — 400 MHz CDCl3

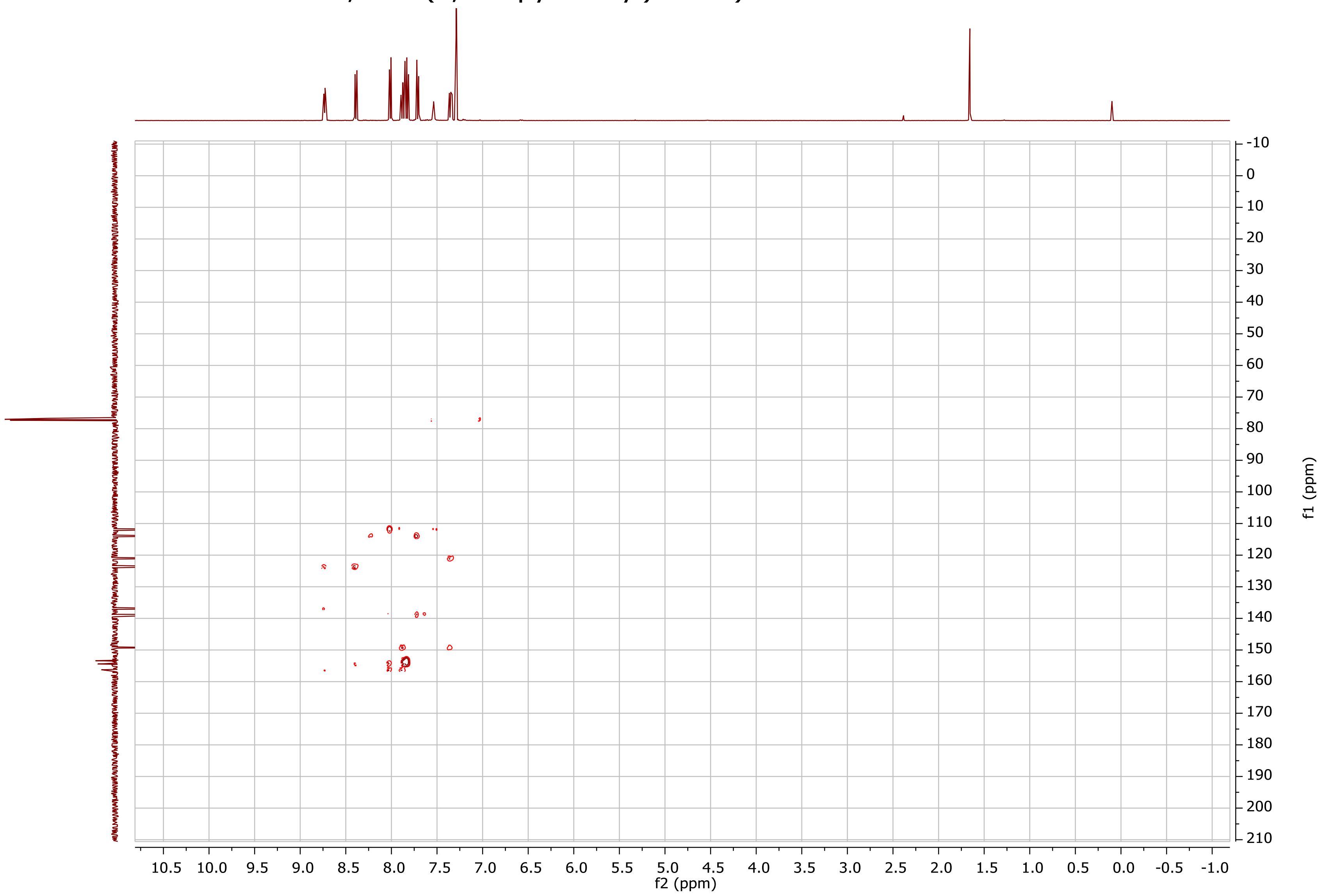

XI. References

1. den Boer, D.; Siberie, Q.; Siegler, M. A.; Ferber, T. H.; Moritz, D. C.; Hofmann, J. P.; Hetterscheid, D. G. H., On the Homogeneity of a Cobalt-Based Water Oxidation Catalyst. *ACS Catal.* **2022,** *12*, 4597-4607.
2. van der Ham, C. J. M.; Işık, F.; Verhoeven, T. W. G. M.; Niemantsverdriet, J. W.; Hetterscheid, D. G. H., Activation pathways taking place at molecular copper precatalysts for the oxygen evolution reaction. *Catal. Today* **2017,** *290*, 33-38.
3. Kottrup, K. G.; D'Agostini, S.; van Langevelde, P. H.; Siegler, M. A.; Hetterscheid, D. G. H., Catalytic Activity of an Iron-Based Water Oxidation Catalyst: Substrate Effects of Graphitic Electrodes. *ACS Catal.* **2018,** *8*, 1052-1061.
4. Wonders, A. H.; Housmans, T. H. M.; Rosca, V.; Koper, M. T. M., On-line mass spectrometry system for measurements at single-crystal electrodes in hanging meniscus configuration. *J. Appl. Electrochem.* **2006,** *36*, 1215-1221.
5. Joachim Demnitz, F. W.; D'Heni, M. B., A HIGH YIELDING PREPARATION OF 2,2′-BIPYRIDINE-1-OXIDE. *Org. Prep. Proced. Int.* **1998,** *30*, 467-469.
6. Yin, J.; Xiang, B.; Huffman, M. A.; Raab, C. E.; Davies, I. W., A General and Efficient 2-Amination of Pyridines and Quinolines. *J. Org. Chem.* **2007,** *72*, 4554-4557.
7. Smith, A. J.; Kalkman, E. D.; Gilbert, Z. W.; Tonks, I. A., ZnCl2 Capture Promotes Ethylene Polymerization by a Salicylaldiminato Ni Complex Bearing a Pendent 2,2′-Bipyridine Group. *Organometallics* **2016,** *35*, 2429-2432.
8. Zheng, S.; Reintjens, N. R. M.; Siegler, M. A.; Roubeau, O.; Bouwman, E.; Rudavskyi, A.; Havenith, R. W. A.; Bonnet, S., Stabilization of the Low-Spin State in a Mononuclear Iron(II) Complex and High-Temperature Cooperative Spin Crossover Mediated by Hydrogen Bonding. *Chem. -Eur. J.* **2016,** *22*, 331-339.
9. Sheldrick, G. M., SHELXT–Integrated space-group and crystal-structure determination. *Acta Crystallogr. A* **2015,** *71*, 3-8.
10. Sheldrick, G. M., Crystal structure refinement with SHELXL. *Acta Crystallogr. C Struct. Chem.* **2015,** *71*, 3-8.
11. Elgrishi, N.; Rountree, K. J.; McCarthy, B. D.; Rountree, E. S.; Eisenhart, T. T.; Dempsey, J. L., A Practical Beginner's Guide to Cyclic Voltammetry. *J. Chem. Educ.* **2018,** *95*, 197-206.
12. Bullock, R. M.; Appel, A. M.; Helm, M. L., Production of hydrogen by electrocatalysis: making the H–H bond by combining protons and hydrides. *Chem. Commun.* **2014,** *50*, 3125-3143.
13. Costentin, C.; Drouet, S.; Robert, M.; Savéant, J.-M., Turnover Numbers, Turnover Frequencies, and Overpotential in Molecular Catalysis of Electrochemical Reactions. Cyclic Voltammetry and Preparative-Scale Electrolysis. *J. Am. Chem. Soc.* **2012,** *134*, 11235-11242.
14. Frisch, M. J.; Trucks, G. W.; Schlegel, H. B.; Scuseria, G. E.; Robb, M. A.; Cheeseman, J. R.; Scalmani, G.; Barone, V.; Petersson, G. A.; Nakatsuji, H.; Li, X.; Caricato, M.; Marenich, A. V.; Bloino, J.; Janesko, B. G.; Gomperts, R.; Mennucci, B.; Hratchian, H. P.; Ortiz, J. V.; Izmaylov, A. F.; Sonnenberg, J. L.; Williams; Ding, F.; Lipparini, F.; Egidi, F.; Goings, J.; Peng, B.; Petrone, A.; Henderson, T.; Ranasinghe, D.; Zakrzewski, V. G.; Gao, J.; Rega, N.; Zheng, G.; Liang, W.; Hada, M.; Ehara, M.; Toyota, K.; Fukuda, R.; Hasegawa, J.; Ishida, M.; Nakajima, T.; Honda, Y.; Kitao, O.; Nakai, H.; Vreven, T.; Throssell, K.; Montgomery Jr., J. A.; Peralta, J. E.; Ogliaro, F.; Bearpark, M. J.; Heyd, J. J.; Brothers, E. N.; Kudin, K. N.; Staroverov, V. N.; Keith, T. A.; Kobayashi, R.; Normand, J.; Raghavachari, K.; Rendell, A. P.; Burant, J. C.; Iyengar, S. S.; Tomasi, J.; Cossi, M.; Millam, J. M.; Klene, M.; Adamo, C.; Cammi, R.; Ochterski, J. W.; Martin, R. L.; Morokuma, K.; Farkas, O.; Foresman, J. B.; Fox, D. J. *Gaussian 16 Rev. C.01*, Wallingford, CT, 2016.
15. Adamo, C.; Barone, V., Toward reliable density functional methods without adjustable parameters: The PBE0 model. *J. Chem. Phys* **1999,** *110*, 6158-6170.

16. Grimme, S.; Ehrlich, S.; Goerigk, L., Effect of the damping function in dispersion corrected density functional theory. *J. Comput. Chem.* **2011,** *32*, 1456-1465.
17. Pliego, J. R.; Riveros, J. M., The Cluster−Continuum Model for the Calculation of the Solvation Free Energy of Ionic Species. *J. Phys. Chem. A* **2001,** *105*, 7241-7247.
18. Barone, V.; Cossi, M., Quantum Calculation of Molecular Energies and Energy Gradients in Solution by a Conductor Solvent Model. *J. Phys. Chem. A* **1998,** *102*, 1995-2001.
19. Andrae, D.; Häußermann, U.; Dolg, M.; Stoll, H.; Preuß, H., Energy-adjustedab initio pseudopotentials for the second and third row transition elements. *Theor. Chim. Acta* **1990,** *77*, 123-141.
20. Hariharan, P. C.; Pople, J. A., The influence of polarization functions on molecular orbital hydrogenation energies. *Theor. Chim. Acta* **1973,** *28*, 213-222.
21. Grimme, S., Supramolecular Binding Thermodynamics by Dispersion-Corrected Density Functional Theory. *Chem. -Eur. J.* **2012,** *18*, 9955-9964.
22. Luchini, G.; Alegre-Requena, J.; Funes-Ardoiz, I.; Paton, R., GoodVibes: automated thermochemistry for heterogeneous computational chemistry data [version 1; peer review: 2 approved with reservations]. *F1000Research* **2020,** *9*.
23. Merrick, J. P.; Moran, D.; Radom, L., An evaluation of harmonic vibrational frequency scale factors. *J. Phys. Chem. A* **2007,** *111*, 11683-700.
24. Zhao, Y.; González-García, N.; Truhlar, D. G., Benchmark Database of Barrier Heights for Heavy Atom Transfer, Nucleophilic Substitution, Association, and Unimolecular Reactions and Its Use to Test Theoretical Methods. *J. Phys. Chem. A* **2005,** *109*, 2012-2018.
25. Krishnan, R.; Binkley, J. S.; Seeger, R.; Pople, J. A., Self-consistent molecular orbital methods. XX. A basis set for correlated wave functions. *J. Chem. Phys* **1980,** *72*, 650-654.
26. Camaioni, D. M.; Schwerdtfeger, C. A., Comment on "Accurate Experimental Values for the Free Energies of Hydration of H+, OH-, and H3O+". *J. Phys. Chem. A* **2005,** *109*, 10795-10797.
27. Marenich, A. V.; Majumdar, A.; Lenz, M.; Cramer, C. J.; Truhlar, D. G., Construction of Pourbaix Diagrams for Ruthenium-Based Water-Oxidation Catalysts by Density Functional Theory. *Angew. Chem. Int. Ed.* **2012,** *51*, 12810-12814.
28. Truhlar, D. G.; Cramer, C. J.; Lewis, A.; Bumpus, J. A., Molecular Modeling of Environmentally Important Processes: Reduction Potentials. *J. Chem. Educ.* **2004,** *81*, 596.
29. Isse, A. A.; Gennaro, A., Absolute Potential of the Standard Hydrogen Electrode and the Problem of Interconversion of Potentials in Different Solvents. *J. Phys. Chem. B* **2010,** *114*, 7894-7899.
30. Bordwell, F. G., Equilibrium acidities in dimethyl sulfoxide solution. *Acc. Chem. Res.* **1988,** *21*, 456-463.
31. Thom, A. J. W.; Sundstrom, E. J.; Head-Gordon, M., LOBA: a localized orbital bonding analysis to calculate oxidation states, with application to a model water oxidation catalyst. *PCCP* **2009,** *11*, 11297-11304.
32. Johnson, E. R.; Keinan, S.; Mori-Sánchez, P.; Contreras-García, J.; Cohen, A. J.; Yang, W., Revealing Noncovalent Interactions. *J. Am. Chem. Soc.* **2010,** *132*, 6498-6506.
33. Lu, T.; Chen, F., Multiwfn: A multifunctional wavefunction analyzer. *J. Comput. Chem.* **2012,** *33*, 580-592.
34. Mitoraj, M. P.; Michalak, A.; Ziegler, T., A Combined Charge and Energy Decomposition Scheme for Bond Analysis. *J. Chem. Theory Comput.* **2009,** *5*, 962-975.
35. Mitoraj, M.; Michalak, A., Natural orbitals for chemical valence as descriptors of chemical bonding in transition metal complexes. *J. Mol. Model.* **2007,** *13*, 347-355.
36. te Velde, G.; Bickelhaupt, F. M.; Baerends, E. J.; Fonseca Guerra, C.; van Gisbergen, S. J. A.; Snijders, J. G.; Ziegler, T., Chemistry with ADF. *J. Comput. Chem.* **2001,** *22*, 931-967.
37. Marcus, R. A., On the Theory of Oxidation-Reduction Reactions Involving Electron Transfer. I. *J. Chem. Phys* **1956,** *24*, 966-978.

38. Caricato, M.; Mennucci, B.; Tomasi, J.; Ingrosso, F.; Cammi, R.; Corni, S.; Scalmani, G., Formation and relaxation of excited states in solution: A new time dependent polarizable continuum model based on time dependent density functional theory. *J. Chem. Phys* **2006,** *124*, 124520.
39. Improta, R.; Barone, V.; Scalmani, G.; Frisch, M. J., A state-specific polarizable continuum model time dependent density functional theory method for excited state calculations in solution. *J. Chem. Phys* **2006,** *125*, 054103.
